# A bench-top Dark-Root device built with LEGO^®^ bricks enables a non-invasive plant root development analysis in soil conditions mirroring nature

**DOI:** 10.3389/fpls.2023.1166511

**Published:** 2023-05-31

**Authors:** Georgi Dermendjiev, Madeleine Schnurer, Ethan Stewart, Thomas Nägele, Giada Marino, Dario Leister, Alexandra Thür, Stefan Plott, Jakub Jeż, Verena Ibl

**Affiliations:** ^1^Department of Functional and Evolutionary Ecology, Molecular Systems Biology (MoSys), University of Vienna, Vienna, Austria; ^2^Plant Sciences Facility, Vienna Biocenter Core Facilities (VBCF), Vienna, Austria; ^3^Faculty of Biology, Plant Evolutionary Cell Biology Ludwig-Maximilians-Universität München, Planegg-Martinsried, Germany

**Keywords:** D-Root, root system architecture, root tracking, #asnearaspossibletonature, Lego®, open-source, crops, barley

## Abstract

Roots are the hidden parts of plants, anchoring their above-ground counterparts in the soil. They are responsible for water and nutrient uptake and for interacting with biotic and abiotic factors in the soil. The root system architecture (RSA) and its plasticity are crucial for resource acquisition and consequently correlate with plant performance while being highly dependent on the surrounding environment, such as soil properties and therefore environmental conditions. Thus, especially for crop plants and regarding agricultural challenges, it is essential to perform molecular and phenotypic analyses of the root system under conditions as near as possible to nature (#asnearaspossibletonature). To prevent root illumination during experimental procedures, which would heavily affect root development, Dark-Root (D-Root) devices (DRDs) have been developed. In this article, we describe the construction and different applications of a sustainable, affordable, flexible, and easy to assemble open-hardware bench-top LEGO® DRD, the DRD-BIBLOX (Brick Black Box). The DRD-BIBLOX consists of one or more 3D-printed rhizoboxes, which can be filled with soil while still providing root visibility. The rhizoboxes sit in a scaffold of secondhand LEGO® bricks, which allows root development in the dark and non-invasive root tracking with an infrared (IR) camera and an IR light-emitting diode (LED) cluster. Proteomic analyses confirmed significant effects of root illumination on barley root and shoot proteomes. Additionally, we confirmed the significant effect of root illumination on barley root and shoot phenotypes. Our data therefore reinforces the importance of the application of field conditions in the lab and the value of our novel device, the DRD-BIBLOX. We further provide a DRD-BIBLOX application spectrum, spanning from investigating a variety of plant species and soil conditions and simulating different environmental conditions and stresses, to proteomic and phenotypic analyses, including early root tracking in the dark.

## 1 Introduction

Plants are sessile organisms; their roots provide anchorage and support for the shoot and are key factors regarding the uptake and translocation of water, nutrients, and the interaction with microbiota (Hodge, 2010; de la Fuente Cantó et al., 2020). Therefore, roots are an essential factor to consider when it comes to plant productivity (Lynch, 1995), as they are important for gravitropic response (Žádníková et al., 2015), serve as storage organs, and interact with the rhizosphere (Zhu et al., 2011). The root system architecture (RSA) describes the spatial configuration of plant roots in the soil (Smith and De Smet, 2012). It includes the embryonic primary root length and the number, angle, and length of lateral roots, adventitious roots, and root hairs (Orman-Ligeza et al., 2013). The RSA differs between dicots and monocots. The taproot system as observed in *Arabidopsis* and tomato consists of the primary root, lateral roots, root hairs, and adventitious roots (Smith and De Smet, 2012). In contrast, monocots, covering barley, maize, and wheat, have a more complex and fibrous root system. Here, the embryonic primary root, including the root hairs, occur along with many types of branched roots, including a massive number of adventitious roots, seminal roots, root–shoot junction crown roots, and lateral roots. The bio-physicochemical properties of the soil dynamically affect the response of the RSA, which depends on the plant genotype and soil conditions.

With regard to crops, the influence of the RSA on resource acquisition efficiency, plant adaptation to environmental changes, and soil–root interactions have been widely studied, since the RSA heavily affects crop productivity (de Dorlodot et al., 2007). The transition from germination to subsequent seedling development is initiated by protrusion of the radicle through the coleorhiza, forming the primary root (Weitbrecht et al., 2011). Concomitant with the formation of the coleoptile, seminal roots and crown roots are formed, constituting the majority of the monocot root system. Seminal roots emerge from the primordia in the embryo of the seed, whereas crown roots are post-embryonically formed and emerge from below-ground surface stem nodes (Smith and De Smet, 2012). Interestingly, the angle of growth and the angle between the first appearing seminal roots at the seedling stage are prototypical of the mature RSA in wheat (Oyanagi et al., 1993; Manschadi et al., 2008) and are subsequently considered as representative trait for mature RSA (Richard et al., 2015).

Root phenotyping is especially important for the identification of root traits and finally for crop yield improvement (reviewed in Tracy et al., 2020). Additionally, huge effort is put into studying the RSA of the conventional dicot model plant *Arabidopsis*, where *in vitro* studies on agar plates (Xiao and Zhang, 2020) and *in situ* studies in rhizotrons have been performed (Rellán-Álvarez et al., 2015; Ogura et al., 2019; LaRue et al., 2022). Image acquisition setups via cameras or scanners often expose roots to light, which heavily affects root development (Cabrera et al., 2022). Recent studies in *Arabidopsis* emphasize the negative effect of light on root development, and scientists consequently shift to implementing DRDs in their approaches (Silva-Navas et al., 2015; Silva-Navas et al., 2016; Garcia-Gonzalez et al., 2021). Thus, to get meaningful results that are applicable to the field, it is indispensable to analyze the root architecture in the dark, in soil conditions mirroring field conditions, especially for crop root phenotyping.

Rhizoboxes have been used for two-dimensional (2D) root visualization since the 1980s (Marschner and Römheld, 1983; Youssef and Chino, 1987; Fitter et al., 1988). In 2D approaches, compared to a possible three-dimensional (3D) root development in pots, roots are forced to grow in 2D along a (glass) slide (Nagel et al., 2015; Bodner et al., 2017), due to angled rhizoboxes and gravitropism. Additionally, since phenotypes in shoots and roots are expressed differently depending on the soil conditions, including water content and temperature, whole-plant phenotyping is emphasized, where roots and shoots are measured simultaneously (reviewed in Tracy et al., 2020). Thus, rhizoboxes are an optimal way to analyze root growth development in parallel to shoot development without affecting the root:shoot ratio (Mašková and Klimeš, 2020).

Currently, the setup of lab experiments is challenging. The coronavirus disease 2019 (COVID-19) pandemic showed us the dependency on an efficient laboratory supply pipeline, since the scientific output was impacted due to lack of lab supplies (Heo et al., 2022). Additionally, a great effort is made by scientists to reduce the anthropogenic climate change, by making research more sustainable.

Inspired by these different challenges, we established a non-invasive, sustainable bench-top DRD that enables whole-plant molecular analysis and phenotyping in conditions as near as possible to nature. We used predominantly secondhand materials (LEGO® bricks), materials 3D-printed in our lab, already available resources, or we bought locally to reduce our CO_2_ footprint. We chose LEGO® bricks to build a dark housing for the rhizoboxes that are flexible in size, resistant to environmental parameters, and easily transportable. Originally used as toy, LEGO® bricks have already inspired a variety of teachers and scientists to translate knowledge and to use these bricks for scientific applications (Lin et al., 2018; Mäntylä and Ihalainen, 2021; Montes et al., 2021). In plant science, LEGO® bricks have recently been used for building small-scale engineered environments for plant roots (Lind et al., 2014).

The LEGO® brick DRD-BIBLOX, short BIBLOX, can house between 1 and 14 in-house made rhizoboxes in a small setup. We show that the BIBLOX can be used for a wide application range, including whole-plant proteomic analysis and root phenotyping of crops grown in different soil compositions mirroring natural field conditions. We also include stress applications and the analysis of the root growth and morphology over real time. The BIBLOX is especially applicable for the analysis of molecular-biology-related investigations using reverse and forward genetic approaches and for analyses of the RSA in response to distinct environmental factors including different substrate compositions.

## 2 Material and methods

### 2.1 Monitoring environmental parameters

Soil temperature, soil water content, light intensity, air temperature, and air humidity were measured in a barley field (9-ha) in Lower Austria in the years 2021 and 2022. For the measurements in the year 2021, a TensioMark® sensor (ecoTech Umwelt-Messsystem GmbH, Bonn, Germany) was used to measure the soil moisture (pF-value), the soil temperature, and the photosynthetically active radiation (PAR). Three sensors run by one data logger were placed within the 9-ha field, with 60 m between measuring points. The sensors were installed at a soil depth of −30 cm. The data were recorded using the data logger “envilog Maxi” (ecoTech Umwelt-Messsystem GmbH, Bonn, Germany). The air temperature was measured with three EasyLog data loggers (Lascar Electronics, Wiltshire, United Kingdom), each positioned in a weather house mounted on a wooden pole, in 80 cm height. The environmental parameters measured in the year 2021 are published on our homepage www.celbics.com and are available in **Supplementary Table S1**. In the year 2022, we used three TekBox-TBSST04-3 (TR) temperature sensors (Umweltanalytische Produkte GmbH, Cottbus, Germany) measuring soil moisture in soil depths of −20, −30, and −50 cm and three PR2/4 SDI-12 Delta-T profile sensors (Umweltanalytische Produkte GmbH, Cottbus, Germany) accumulating the data in −10, −20, −30, and −40 cm of soil depth, respectively (**Supplementary Figure S1A**). The data were recorded and saved by one solar powered data logger (yDoc ML-317, Firmware version 4.3 build 8) (Umweltanalytische Produkte GmbH, Cottbus, Germany). Three quantum sensors (Apogee model SQ-421) were used to measure the PAR in the field. Measurements were performed every 15 min and saved to an SD card. After the end of the field trial, the data were exported into. csv format via the Software “ydocTerminal” version 3.13. Air temperature was measured with three EasyLog data loggers (Lascar electronics, Wiltshire, United Kingdom), each positioned in a weather house mounted on a wooden pole, in 80 cm height (**Supplementary Figure S1A**). The data were imported into “RStudio” version 2022.12.0 + 353 (RStudio Team, 2020) with “R core” version 4.2.2 (R Core Team, 2022). Rows with missing values were filtered out and soil moisture sensor 3, which delivered only very few data points (probably due to voltage drop in the cable) and one of the soil temperature sensors at −20 cm depth, which got damaged during the setup and sent incorrect data, were filtered out as well. Air temperature and humidity data were imported from the three sensors and merged. Data were subset for the first 16 days, the wanted sensor (and depth for soil moisture and temperature), and transformed into long format using the “melt” function from the R-package “reshape2” (Wickham, 2007). Plots were created using the R-package “ggplot2” (Wickham, 2016) with the color palette “Set2” from the R package “RColorBrewer” version 1.3-3 (Neuwirth, 2022). PAR and soil temperature data points aligned almost perfectly for the replicates and were therefore only drawn using the “geom_line” function. Data points of the soil moisture and air temperature showed greater variability; therefore, smoothened means of the data points were plotted using the function “geom_smooth” with the parameters: method = “loess” and a span of 0.1 for the soil moisture and 0.01 for air temperature (**Supplementary Data 1**).

### 2.2 Construction of the BIBLOX

#### 2.2.1 Materials for DRD-BIBLOX

Materials for DRD-BIBLOX were as follows:

.) 3D-CAD software Fusion 360 (Autodesk Inc., San Rafael, CA, USA)
.) 3D printer Ultimaker S5
.) Polylactic acid (PLA) filament for 3D printing
.) Rhizoboxes, 200 mm × 150 mm × 30 mm (height × width × depth)
.) Studio 2.0 BrickLink Studio software
.) LEGO® DRD-BIBLOX (composed of around 830 black LEGO® bricks and 77 plates (see Excel file and studio 2.0 file))
.) Infra-red (IR) LED Cluster_880 nm 5 mm T-1 3/4 (Kingbright, BL0106-15-29)
.) Glass, regular quality, 13.7 mm × 19.4 mm × 2 mm (height × width × depth)
.) Plant growth chamber (Conviron)
.) Raspberry Pi3 B+ single board computer
.) Pi3 Camera (Electreeks® Raspberry Pi camera module with an automatic infrared cut filter—full HD
.) 75.5° standard tripod
.) Foam rubber, 1.7 mm thickness
.) ImageJ Software (https://imagej.nih.gov)
.) The R Project for data analysis
.) Dark tape

#### 2.2.2 Rhizobox setup and design

**Figure 1:**
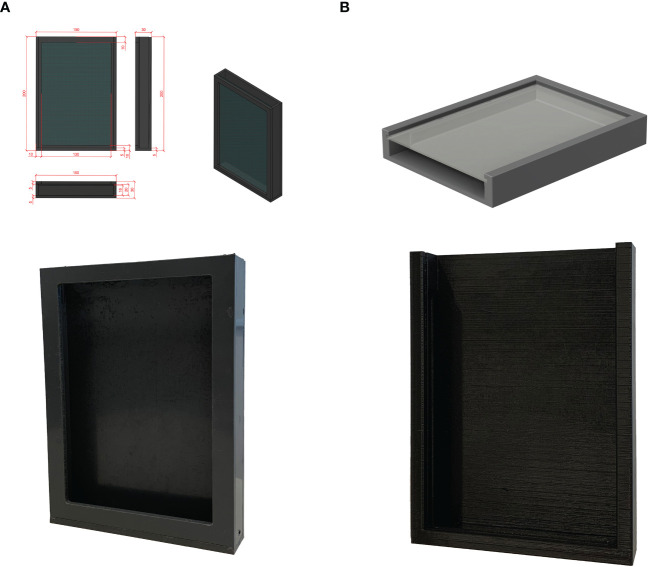
Blueprint and image of rhizoboxes constructed with PVC sheets **(A)** and 3D-printed version **(B)**.

Initial rhizoboxes were constructed from 5 mm polyvinyl chloride (PVC) sheet and bonded with silicon-based glue in house (**Figure 1A**). The glass was positioned loosely at the front side and was later stabilized by the added soil. Additionally, 3D-printed versions were designed using the 3D-CAD software Fusion 360. The 3D models are presented in **Supplementary Figure S2**, and the corresponding file is available in **Supplementary Data 2**. The .stl file is freely accessible at https://www.thingiverse.com/thing:5973755. Rhizoboxes were printed using an Ultimaker S5 3D with PLA filament (**Figure 1B**). Dimensions of both rhizobox versions are 200 mm × 150 mm × 30 mm (height × width × depth). The glass was positioned into the rail of the 3D-printed rhizobox.

#### 2.2.3 Setup of the BIBLOX

We used the freely available software Studio 2.0 BrickLink Studio (https://www.bricklink.com/r3/studio/download.page#xlink) for computer-aided design of the BIBLOX (**Supplementary Data 3**). The quantity and ID number of the used bricks is available in **Supplementary Table S2**. Black secondhand LEGO® bricks were used to construct the base of the BIBLOX. Special LEGO® bricks and plates, which were not available pre-owned, were bought locally. Only a few rare bricks were bought online from LEGO® Systems A/S (https://www.lego.com/en-gb). The construction of a single BIBLOX took approximately 60 min. The rhizobox was positioned at an angle of 60° within the BIBLOX against rails attached to the inner brick walls. To protect the roots completely from light, dark tape was used to close the gap between rhizoboxes in sampling approaches and to cover holes for cables.

#### 2.2.4 Image acquisition

Once the BIBLOX was constructed, we installed the Pi3 camera and the IR LED Cluster within the box. The Pi3 camera was mounted on a 75.5° standard tripod and connected to the Raspberry Pi3 B^+^ computer placed outside the box. The computer was connected to a LAN via en ethernet cable (Wi-Fi would also be possible). The camera was set to capture images every 4 min. The IR LED Cluster source was controlled by a relay (Raspberry Pi Power Relay Board Expansion Board Module Three Channel (3-ch)) installed on the Raspberry Pi3 computer, which switched the light source on for only 1 s during image acquisition. In this way, we limited root light exposure to a minimum. To reduce reflections during imaging, foam rubber was positioned inside the BIBLOX. Additionally, a dark tape was used to diminish the incoming light from the cable hole.

The costs for one BIBLOX including the setup for non-invasive root tracking run at approximately 520€.

### 2.3 Plant materials and growth condition

The spring barley (*Hordeum vulgare* L.) wild-type variety Golden Promise (GP) and the facultative variety BCC93 (kindly provided by Kerstin Neumann, IPK Gatersleben) were grown in the plant growth chamber (PSI) at 14 °C/12 °C, and maize and wheat (Bobwhite) at 16 °C/14 °C, 12 h day/12 h night cycle with light intensities of 130–220 µmol m^−2^ s^−1^ and 70% humidity. To track early root growth, GP and BCC93 were grown as near as possible to nature, according to Dermendjiev et al. (2021), and the measured data of the field experiments, at 14 °C/12 °C, 12 h day/12 h night cycle with light intensities of 130–220 µmol m^−2^ s^−1^ and 70% humidity, in a plant growth chamber (Conviron Adaptis A1000). Tomato was grown in the glass house at 26 °C/19 °C, 12 h day/12 h night cycle with light intensity between 200–250 µmol m^−2^ s^−1^ and between 50% and 60% humidity.

### 2.4 Soil compositions

Within this project, we used five different soils. (I) For the growth of tomato, maize, and wheat, we used sieved (3 mm mesh) Cocopeat supplied with H_2_O. (II) To enable a high root highlighting for the imaging, we used sieved (3 mm mesh) Cocopeat that was mixed with activated charcoal 2:1, supplied with H_2_O (henceforth “Cocopeat_black”). Peat substrate (Gramoflor) was supplied with H_2_O. (III) We used naturally grown bio-organic field soil and (IV) naturally grown conventional field soil (V) (that were obtained from fields in Lower Austria). The bio-organic field soil and conventional field soil was analyzed by the Austrian Agency for Health and Food Safety GmbH, in short AGES (Vienna, Austria). Bio-organic field soil shows a higher pH value (pH = 6.3) and less mineral nitrogen (0.3 mg/100 g) compared to conventional field soil (pH = 5.5; mineral nitrogen, 0.5 mg/100 g) (**Supplementary Data 4**). For salt stress analysis, we mixed activated charcoal and Cocopeat with water containing 20 g NaCl/l H_2_O (electric conductivity (EC) = 30 EC). Soils were adjusted to a pF-value 2–3 to enable a water content mirroring soil environmental parameters as near as possible to nature and were added to the rhizoboxes, respectively. The pF-value was constant for 16 days; thus, for experiments shown in this manuscript, plants were not additionally watered.

### 2.5 Sampling for proteomic analyses

For proteomic analyses, root and shoot material of the barley trait GP was used. Barley grains were germinated in rhizoboxes filled with soil (Cocopeat with activated charcoal). Seven grains were sowed per rhizobox. For control conditions, rhizoboxes were inserted into a BIBLOX setup in a climate chamber; therefore, roots would develop in the dark. For root illumination conditions, stand-alone rhizoboxes were put directly in a climate chamber without covering the roots in the rhizobox (**Supplementary Data 3**). All rhizoboxes were installed at an angle of 60° allowing root growth along the glass front of the boxes. Conditions in climate chambers (Conviron Adaptis A1000, Controlled Environment limited) were set to 12 h day/12 h night cycles of 14 °C/12 °C, with a light intensity of 130–220 µmol m^−2^ s^−1^ and 70% humidity. No difference in the soil temperature was measured between the stand-alone rhizoboxes and the rhizoboxes covered by the BIBLOX.

Seedlings were harvested 8 and 16 days after sowing (DAS), respectively. Root (R) and shoot (S) material was separately harvested, cleaned from soil, and immediately frozen in liquid nitrogen (LN_2_). Shoots and roots of plants with light grown roots (LGRs) were harvested under light conditions (S_LGR + LGR). For plants of dark grown roots (DGRs), the shoot was harvested in light, and the root was harvested in a completely dark room with dimmed red light to prevent root illumination (S_DGR + DGR). Combined root or shoot material of 14 plants for 8 DAS and seven plants for 16 DAS would count as one biological replicate, respectively. Three to four biological replicates were taken per category (8 DAS_S_DGR, 8 DAS_S_LGR, 8 DAS_DGR, 8 DAS_LGR, 16 DAS_S_DGR, 16 DAS_S_LGR, 16 DAS_DGR, and 16 DAS_LGR).

### 2.6 Protein extraction and digestion

Material was homogenized to powder, using LN_2_ and mortar and pestle. Proteins were extracted using a sucrose SDS buffer [100 mM Tris–HCl pH 8.0, 30% (w/v) sucrose, 0.5% (v/v) 2-mercaptoethanol, 10 mM EDTA, 2% (w/v) SDS, Protease Inhibitor (Roche, Cat. No. 05 892 791 001)] by adding 1 ml of buffer to 350 mg of sample. Samples were resuspended completely. A total of 750 µl ROTI®Phenol [Roth, Cat. No. 0038.3] was added to the samples for protein extraction. Samples were vortexed for 1 min and incubated for 5 min followed by centrifugation at 20,000×*g* for 5 min at room temperature (RT). After phase separation, the phenol phase was carefully transferred to a new tube. The phenol fractions were counter-extracted with 750 µl of sucrose SDS buffer, vortexed for 1 min and incubated for 5 min, and then centrifuged at 20,000×*g* for 5 min at RT. The phenol phase was carefully transferred to a new reaction tube. Proteins were precipitated by adding 2.5 volumes of ammonium acetate in methanol [0.1 M]. After 16 h incubation at −20 °C, proteins were pelleted by centrifugation at 4 °C for 5 min at 5,000×*g*. Supernatants were discarded, and the protein pellets were washed with ice-cold ammonium acetate in methanol [0.1 M] and 70% methanol, respectively, followed each by centrifugation at 4 °C for 2 min at 18,000×*g*. The supernatant was removed, and protein pellets were air dried for 60 min and subsequently resuspended in 50 µl urea buffer [8 M urea, 100 mM ammonium bicarbonate, 5 mM dithiothreitol (DTT), Protease Inhibitor] while incubated at 37 °C for 20 min for better solubility. Next, samples were centrifuged at RT at 20,000×*g* for 10 min.

Protein concentration was measured via Bradford assay using a Quick Start™ Bradford 1× Dye Reagent (Biorad, Cat. No. 5000205) prior to protein content normalization. Bovine serum albumin (BSA) dilution series (0–10 mg/ml) in the according buffer was used as standard to calculate sample protein concentration. A total of 2 µl of sample or standard was pipetted into 1.5-ml tubes (in triplicates). 1 ml of Bio-Rad Quick Start™ Bradford 1× Dye Reagent was added. Tubes were vortexed and incubated in the dark for 10 min. A total of 200 µl of the solution was transferred into a 96-well plate. The absorbance of standards and samples was measured at 595 nm wavelength using a Thermo Scientific Multiskan Spectrum. BSA standard curve and calculation of protein concentration were done using Microsoft Excel. Cystein residues were reduced by incubating 200 μg protein per sample for 45 min at 30 °C while shaking at 700 rpm. Cysteine residues were alkylated with 55 mM Iodoacetamide (IAA) while shaking at 700 rpm, in the dark, at RT, for 60 min. Increased DTT [10 mM] concentration and sample incubation at RT and shaking at 700 rpm for 15 min stopped the alkylation process.

Furthermore, the urea concentration was diluted to 2 M with 100 mM ammonium bicarbonate/10% acetonitrile (ACN). CaCl_2_ was added to a final concentration of 2 mM. Trypsin digestion was performed at 37 °C rotating for 14–16 h using Poroszyme™ Immobilized Trypsin Cartridge (ThermoScientific Cat. No. 8-0087-40-0994) at a ratio of 1:20 v:w.

Peptides were desalted using C18 solid-phase extraction columns (Bond Elut SPEC C18, 96 round-well plate, 15 mg, 1 ml, Agilent Technologies, Santa Clara, USA) and a water-jet (vacuum) pump. Plates were activated with 2×400 µl methanol passing the columns by gravity for 2 min and then aspirated via the pump. Columns were equilibrated with 4×400 µl of ultrapure H_2_O, passing the column by gravity for 2 min and then aspirated via the pump. Subsequently, samples were pipetted into column and peptides and salt bound to it while gravity flow for 5 min, followed by aspiration via the pump. Samples were desalted with 5×400 µl of ultrapure H_2_O passing the column by gravity for 2 min and then aspirated via the pump, last aspiration to total dryness. Purified peptides were recovered with 2×200 µl of methanol, passing the column by gravity for 5 min and then total aspirated *via* the pump. Peptides were transferred into new tubes and dried in a SCANVAC CoolSafe Vacuum Concentrator for 5 h at RT. The peptides were resuspended in 0.1% formic acid (FA) in acetonitrile. The final peptide concentration was measured spectrophotometrically via a NanoDrop device (Thermo Scientific).

### 2.7 LC-MS/MS analysis

Liquid chromatography and tandem mass spectrometry analysis was performed on a nano-LC-system (Ultimate 3000 RSLC; Thermo Fisher Scientific) coupled to an Impact II high-resolution quadrupole time-of-flight (Bruker) using a Captive Spray nanoelectrospray ionization source (Bruker Daltonics). The nano-LC system was equipped with an Acclaim Pepmap nanotrap column (C18, 100 Å, 100 µm, 2 cm; Thermo Fisher Scientific) and an Acclaim Pepmap RSLC analytical column (C18, 100 Å, 75 µm × 50 cm; Thermo Fisher Scientific). The peptide mixture was fractionated by applying a linear gradient of 5%–30% solvent B [0.1% FA in acetonitrile] at a flowrate of 250 nl min^−1^ over a period of 60 min, followed by a linear increase of 30%–45% solvent B within 15 min. The column temperature was set to 50 °C. MS1 spectra were acquired at 3 Hz with a mass range from *m*/*z* 200 to 2,000, with the Top-18 most intense peaks selected for MS/MS analysis using an intensity-dependent spectra acquisition time between 4 and 16 Hz. Dynamic exclusion duration was 0.5 min.

### 2.8 Data analysis and visualization

Proteomics MS raw files were processed using the MaxQuant software (version 2.0.30; Cox and Mann, 2008). Peak lists were compared against the barley reference proteome (*Hordeum vulgare* subsp. *vulgare* (domesticated barley), cv. Morex, UniProt, UP000011116, version March 2022) using the built‐in Andromeda search engine (Cox et al., 2011). Enzyme specificity was set to trypsin, allowing up to two missed cleavages. Cysteine carbamidomethylation was set as static modification, and N-terminal acetylation and methionine oxidation as variable modifications. During the search, sequences of 248 common contaminant proteins and decoy sequences were automatically added. A false discovery rate (FDR) of 1% was set at peptide and protein level. Proteins were quantified across samples using the label-free quantification (LFQ) algorithm (Cox et al., 2014), and the match-between-runs option was enabled.

Uncharacterized proteins were manually identified by using the UniProt BLAST application.

Data were analyzed and visualized using Microsoft Excel (version 2211 Build 16.0.15831.20098 for Microsoft 365 MSO) and RStudio (version 2022.02.2 for Windows).

Proteins of which LFQ values were not detected for any of the measured sample groups and proteins where three out of four or two out of three values of biological replicates of one sample group were missing were dismissed. For missing third (for three replicates) or forth (for four replicates) values, an average value was calculated from the other two of three values of the biological replicates (**Supplementary Table S3**).

The T.TEST function (heteroscedastic, with two-tailed distribution; Microsoft Excel) was used to find significant differences regarding LFQ values between mean values of different sample groups (**Supplementary Table S3**). Prior to principal component analysis (PCA), data were logarithmically normalized using log_10_(x+1). PCAs, loading plots, and contribution plots of all data, and subgroups were calculated and visualized using RStudio (**Supplementary Table S4**, **Supplementary Data 5**). Proteins that significantly differed in their abundance when comparing plants (shoots and roots) of LGRs and DGRs were classified regarding subcellular localization and molecular function using Microsoft Excel. Subcellular localization categories were “Cytoplasm,” “Cytosol,” “Nucleus,” “Mitochondria,” “integral component of membrane,” “Ribosome,” “Chloroplast,” “Extracellular,” “Plasma membrane,” “Cytoskeleton,” “Endoplasmic reticulum,” “Golgi apparatus,” “Peroxisome,” “Vacuole,” “Cell wall,” “Apoplast,” and “Plasmodesmata.” Molecular function categories included among others “RNA binding,” “ATP binding,” “Metal ion binding,” “Oxidoreductase activity,” “Defense response activity,” “Cytoskeleton,” and “Actin filament binding” (**Supplementary Table S3**).

### 2.9 Image analysis

To enhance the contrast between the roots and substrate for further semi-automatic image analyses, we manually traced the roots in every 10th image from each experiment (derived from between three and five biological replicates) with an Apple Pencil on an iPad (**Supplementary Figures S3A, B**), since our group works paperless and has been using those tools as digital lab notebooks. From the traced images, a binary image of the root system was made using color thresholding. Binary images were skeletonized, and a network graph was constructed using the sknw package (Xiaolong, 2020). From the network graph, the longest root and total root system length were calculated using the network-x package (Hagberg et al., 2008). Primary root angle was calculated by fitting a line through the x and y coordinates of the primary root skeleton pixels. The convex hull area and bounding box width of the root system were calculated from the binary images using OpenCV (Bradski, 2000) (**Supplementary Figure S3C**).

The root growth angle (RGA) and the seminal root growth angle (SRGA) were measured with the angle tool provided in ImageJ (Schneider et al., 2012) by drawing lines from the grain to the maximum distance of the seminal root to the horizontal level of the grain and between the first two seminal roots (**Supplementary Figure S3D**). Data were analyzed and visualized using GraphPad Prism (version 9.0 for Mac, GraphPad Software, San Diego, CA, USA, http://www.graphpad.com/).

## 3 Results

### 3.1 Monitoring the environmental parameters enables lab experiments as near as possible to nature

Recently, we have successfully set up conditions to follow the germination in the lab at parameters as near as possible to nature (Dermendjiev et al., 2021). We monitored the soil temperature and moisture, air temperature, and PAR in a field of an organic spring barley farmer in lower Austria between the period of sowing and harvesting barley within 2021 and 2022, respectively. The recorded data of 2021 (**Supplementary Table S1**), which are publicly available on our group homepage (www.celbics.com), show a pF-value in − 30 cm depth for the first 16 days between 2 and 2.5. Additionally, the soil temperature was between 6 °C and 13 °C in soil depth of −30 cm. In 2022, the soil temperature was between 7.3 °C and 18.2 °C in −20 cm soil depth resulting in a mean temperature of 12 °C (**Supplementary Figures S1A, B**). The soil water content was between 14% and 20% (v/v). Thus, the parameters measured in year 2022 were consistent with the environmental parameters measured in the year 2021. Subsequently, the temperature for barley germination in the lab condition was set to the temperature of 14 °C/12 °C, considering that barley grains are sown at -3 cm below the soil surface and that air temperature was measured between 0 °C and 25 °C within these first 16 days. The soil moisture was set for the germination to a pF-value between 2-3 during the first 16 days of germination.

### 3.2 The construction of rhizoboxes for non-invasive *in situ* early root tracking: from in-house made PVC rhizoboxes to a 3D printed version

Rhizoboxes were constructed from PVC for the purpose of using them as DRD and as stand-alone devices. To reduce the CO_2_ footprint and increase flexibility, subsequently, rhizoboxes were 3D printed with an in-house 3D printer using polylactic acid (PLA), a thermoplastic polymer that is manufactured from renewable and biodegradable plant-based materials (Henton et al., 2005; Bhatia et al., 2007) (**Figure 1**). Additionally, PLA is able to withstand plant growth conditions. All rhizoboxes are reusable. This 3D-printed version enables high flexibility in terms of construction size and production timepoint. Additionally, using 3D printers is a first small step for more sustainable research in the lab.

### 3.3 The diverse applications of the BIBLOX for non-invasive *in situ* early root development analysis

Applying the previously measured natural environmental parameters enables us to perform experiments in controlled lab conditions with settings as near as possible to nature.

#### 3.3.1 BIBLOX as system for Dark-Root growth analysis of several crop plants and different soil conditions

##### 3.3.1.1 BIBLOX as DRD that enables root growth in the dark and shoot growth in the light

**Figure 2:**
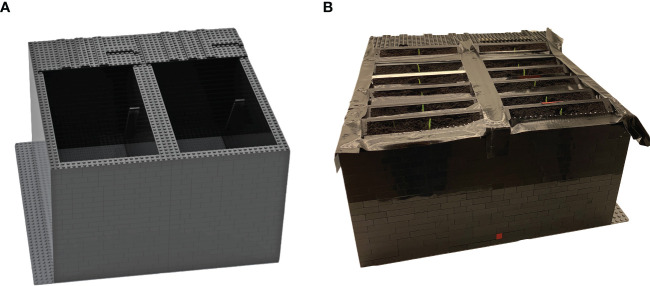
The BIBLOX for sampling plants of DGRs. The Studio 2.0 BrickLink Studio software was used to virtually design the BIBLOX for 12–14 rhizoboxes **(A)** that was finally constructed with black LEGO® bricks **(B)**. Note the dark tape to close the gap between several rhizoboxes to diminish the income of light. PVC- and 3D-printed rhizoboxes were used.

**Figure 3:**
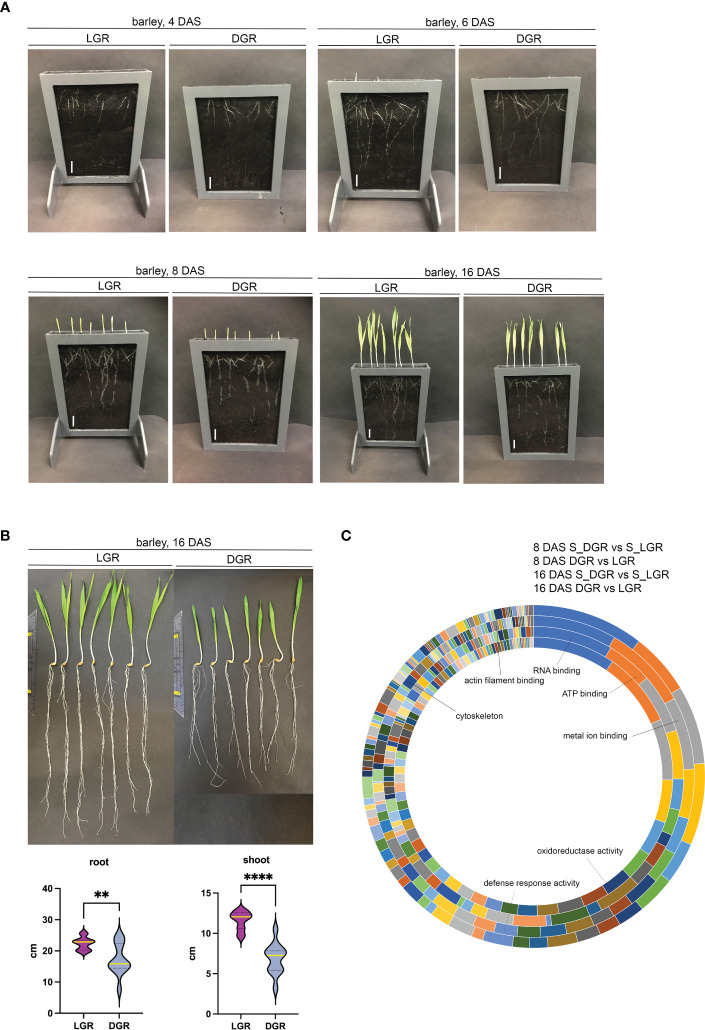
Barley growth and proteomic analysis of roots and shoots of DGR and LGR plants. **(A)** Pictures of barley (GP) seedlings in rhizoboxes grown in Cocopeat_black were taken at 4, 6, 8, and 16 DAS, respectively. Seven grains were sown per rhizobox (here out of PVC). Scale = 0.5 cm. **(B)** Root and shoot length of plants grown for 16 DAS. Plants were harvested from the rhizobox and washed. Light grown roots (LGR), dark grown roots (DGR). All shoots were in light. Violin plots show the root and shoot size of roots grown in the light (n = 12) and dark (n = 12). ** represents ≤0.005, **** represents ≤0.0001. Yellow line represents the median. **(C)** Functional classification of significantly up- or downregulated proteins in the root and shoot upon root illumination. Indicated proteins are involved in RNA, ATP, and metal ion binding, in the oxidoreductase and defense activity, and cytoskeleton-related proteins. Annotation from outer to inner circle: 8 DAS_S_DGR vs. S_LGR; 8 DAS_DGR vs. LGR; 16 DAS_S_DGR vs. S_LGR; 16 DAS_DGR vs. LGR. DAS, days after sowing; S_DGR, shoot of dark grown root; S_LGR, shoot of light grown root; LGR, light grown roots; DGR, dark grown roots. Cocopeat_black refers to Cocopeat including activated charcoal.

Root illumination heavily affects root development: recent data show that cytoskeleton proteins (Dyachok et al., 2011; Du et al., 2020; Halat et al., 2020; Cabrera et al., 2022) and proteins involved in the reactive oxygen species (ROS) pathway are affected by light (Yokawa et al., 2011). Thus, our demands on the DRD were to provide an easily constructible, flexible in size, resistant to environmental parameters, and sustainable bench-top system for phenotypical and molecular analyses of root and shoot material. Furthermore, it should easily be transferred to and placed in growth chambers and glass houses where appropriate environmental parameters are set (**Figure 2A**). To test the performance of the BIBLOX as a suitable DRD, experiments were set up, aiming to show differences in whole plant phenotype and molecular structure when comparing plant tissues of DGRs and LGRs. The gathered data would, on the one hand, reinforce the existing data on the huge effect of root light exposure on the whole plant and, additionally, the applicability of our device as DRD would be tested. Thus, we built a BIBLOX setup that covers 12 rhizoboxes (**Figure 2B**). We performed phenotypic and proteomic analysis of root and shoot material of plants of LGRs compared to plants of DGRs. For this approach, rhizoboxes were filled with soil, and seven barley grains per rhizobox were put for germination. The BIBLOX with rhizoboxes was put into the growth chamber for up to 16 days. Assessment of the incoming light to the BIBLOX showed a 92%–95% reduction in light intensity measured at root level (< 10 µmol m ^2^ s^−1^ in the BIBLOX compared to 130–220 µmol m^−2^ s^−1^ in the Conviron). Additionally, stand-alone rhizoboxes, were positioned at an angle of 60°, without coverage and mounted on a skeleton of LEGO® (**Supplementary Data 3**) were installed in the climate chamber, allowing root illumination of developing plants. Pictures were taken at 4, 6, 8, and 16 DAS. A delay in barley root and shoot development in plants of DGRs compared to plants of LGRs could be observed (**Figure 3A**). After 16 days, plants were removed from the rhizoboxes, the roots were washed, and images were taken to assess the final root and shoot growth size (**Figure 3B**). DGRs were slightly smaller compared to LGRs (**Figures 3A, B**). Interestingly, regarding phenotypes, the shoot was much more affected, since the shoot length of the plants of DRGs were significantly reduced compared to the plants of LGRs (**Figure 3B**).

For proteomic sampling, plant roots were kept continuously in the dark or light for 8 and 16 days. Finally, 8 and 16 DAS roots and shoots were harvested according to their growth light settings. This was followed by protein extraction and digestion and subsequent proteomic analysis. For the root and shoot material, in total, we identified 2,158 proteins; 1,236 of them showed significant changes in their abundance in root and shoot following root illumination. Out of all significantly different regulated proteins upon root light exposure, in 8 DAS roots, approximately 50% (68% for 16 DAS) were downregulated and 50% (32% for 16 DAS) were upregulated compared to dark conditions. While in 8 DAS shoots of plants of LGRs (S_LGR), approximately 47% (38% for 16 DAS) of all significantly differently regulated proteins were downregulated, and 53% (62% for 16 DAS) were upregulated compared to S_DGRs. PCAs of 8 DAS or 16 DAS only and of all data combined showed a clear separation between sample groups and clustering of biological replicates within sample groups (**Supplementary Figures S4**-**S6**). Principal component 1 (PC1) separates the proteins regarding root and shoot specificity. PC2 separates the proteins according to root illumination (**Supplementary Figures S4**, **S5**). The PCA of 8 DAS data shows that proteins of LGRs and the corresponding shoots are clearly separated from proteins of DGRs. Additionally, at 8 DAS, proteins of roots and shoots are distinctively separated, too. At 16 DAS, proteins of roots and shoots are clearly separated as well. However, proteins of LGR plants show only specific separation in roots but not in the corresponding shoots (**Supplementary Figures S4**, **S5**). According to the PCA plots, the effect of root illumination on the tissue-specific protein abundance at 16 DAS appears stronger in the roots compared to the effect in shoots (**Supplementary Figures S4**-**S6**). The subcellular classification of significantly up- or downregulated proteins in root and shoot upon root illumination showed a broad range of protein localizations (**Supplementary Table S3**). Furthermore, a classification of molecular functions of those proteins showed them being highly involved in RNA binding, ATP binding, metal ion binding, and in oxidoreductase activities, defense response activities, the cytoskeleton, and actin filament binding (**Figure 3C**). Additionally, we found differently regulated protein levels of, for example, ROS-associated proteins, auxin-pathway-associated proteins, defense-response-associated proteins, and cytoskeleton-related proteins upon root light exposure.

These data confirm already published effects of light on roots, e.g., on cytoskeleton proteins (Dyachok et al., 2011; Du et al., 2020; Halat et al., 2020; Cabrera et al., 2022) and on ROS (Yokawa et al., 2011).

##### 3.3.1.2 BIBLOX enables root growth analysis of distinct crops under natural soil conditions and allows stress application

**Figure 4:**
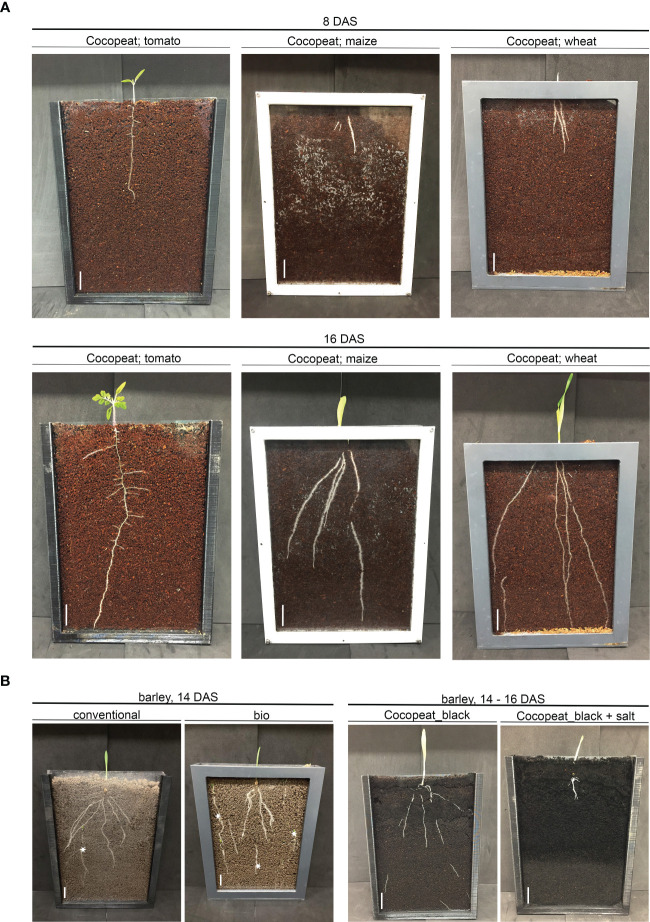
The BIBLOX enables the root growth analysis of distinct crops and the application of nature environmental conditions. **(A)** Plant growth of tomato, maize, and wheat, in Cocopeat. n = 8–13. Representative pictures were taken at 8 and 16 DAS. **(B)** Barley (GP) was sown in conventional field soil and in bio-organic field soil. n = 3. A representative picture was made at 14 DAS. Note the appearance of weeds in the natural field soil, indicated with *. Barley (GP) was sown in Cocopeat_black + salt (30 EC) n = 3. A representative picture was made at 14–16 DAS. Scale = 0.5 cm. Cocopeat_black refers to Cocopeat including activated charcoal. 3D-printed and PVC rhizoboxes were used.

Aiming to establish a DRD with a broad application spectrum, we further evaluated the application of a BIBLOX for growth analysis for additional crops. Tomato, maize, and wheat were grown in Cocopeat under appropriate settings. The RSAs of the used crops could be clearly observed at 8 and 16 DAS (**Figure 4A**).

Since non-natural soil conditions alter the root development, the next step with respect to accurate controlled lab experiments was the application of natural soil conditions. Subsequently, we applied naturally grown bio-organic field soil and conventional field soil and analyzed the root development of barley (GP) grown in the BIBLOX. RSA of 14 DAS was clearly different from roots grown in bio-organic field soil compared to conventional field soil (**Figure 4B**). Additionally, barley (GP) was exposed to salt stress (30 EC) during germination and early root development, which corresponds to salt-tolerant conditions that barley, as salt-tolerant plant, is able to handle (**Figure 4B**). These data show the diverse application possibilities of the BIBLOX to study the RSA of different crops and different soil conditions.

#### 3.3.2 BIBLOX as a system for uninterrupted root growth and morphology analysis over time

**Figure 5:**
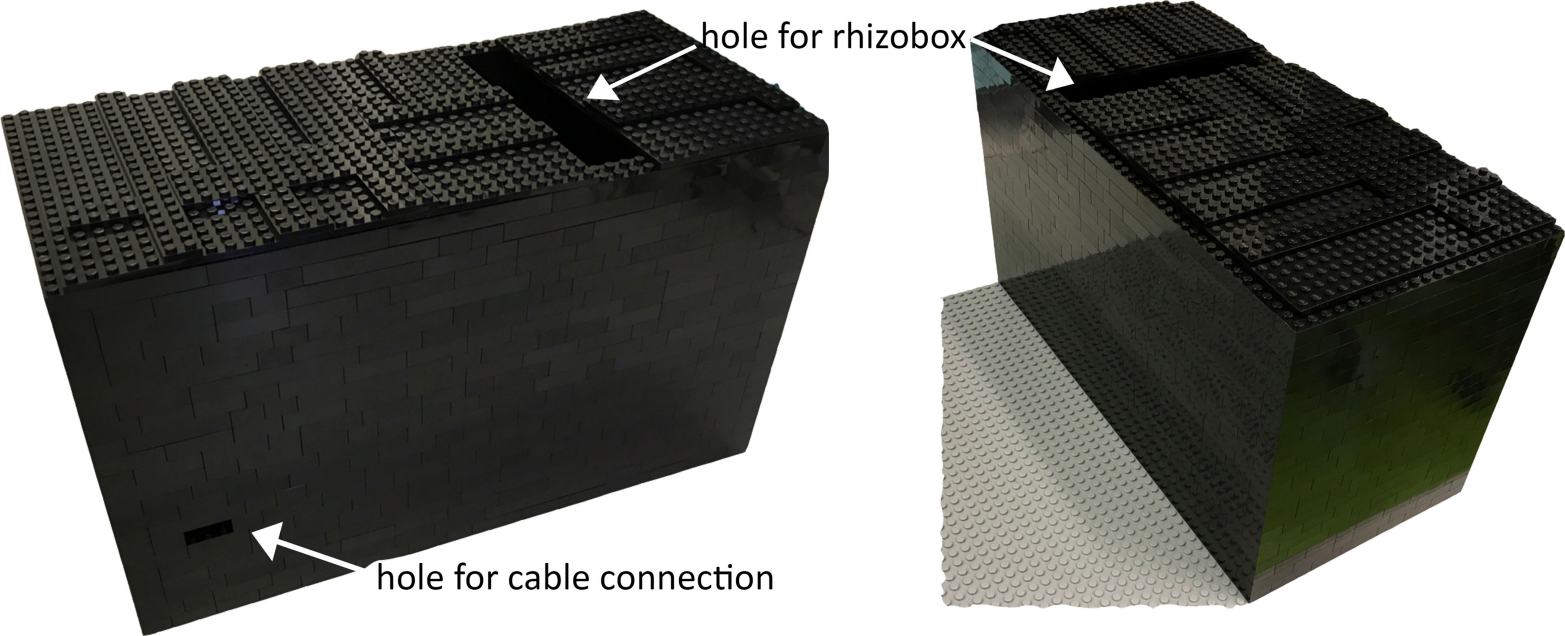
Construction of the BIBLOX. The finished BIBLOX (here for a single tracking experiment) is in total 22–23 rows high and includes place for the light source, the camera, and one rhizobox. Note the hole for inserting the rhizobox and one hole for the cables from the power supply and the Raspberry Pi3 system.

**Figure 6:**
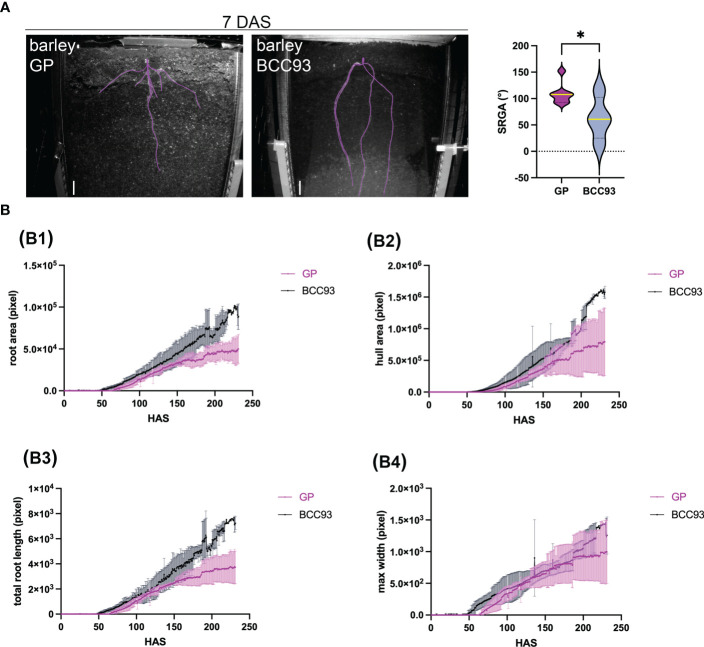
Analysis of root growth and morphology of different barley traits over time. **(A)** Analysis of the seminal root growth angle (SRGA) at 7 DAS. The Violin plot shows a significant difference of the SRGA between the barley traits GP and BCC93 (n = 4–7). * represents ≤0.05. **(B)** Root traits measured over time (hours after sowing, HAS), convex hull area (B1), total root length (B2), maximum root system width (B3), and primary root angle (B4) for three to six biological replicates. Plants were grown in Cocopeat_black, which refers to Cocopeat including activated charcoal. Scale = 0.5 cm. 3D printed rhizoboxes were used.

To avoid root illumination during root development, we set up a non-invasive root tracking method that enables an uninterrupted root growth. We built the BIBLOX, which covers one rhizobox, a light source, and the camera for early *in situ* root tracking (**Figures 5**, **6**; **Supplementary Figure S7**). We used approximately 830 black LEGO® bricks for the base, approximately 80 plates, one base plate, and eight special pieces for the two holders of the rhizobox (**Supplementary Datas 2**, **3**). In total, the BIBLOX includes 23 rows of LEGO® bricks (**Figure 5**; **Supplementary Data 3**), where the holders were placed from the 6th to the 16th row. For the independent biological replicates, we used one BIBLOX for the image analysis. However, upscaling the system is possible with up to three parallel BIBLOXes per shelf within our Conviron plant growth chamber (**Supplementary Figure S7**; **Supplementary Data 3**). Of course, downscaling for smaller rhizoboxes, e.g., for *Arabidopsis*, would also be possible.

Four to seven biological replicates were analyzed. The semi-automated root phenotyping method allowed us to analyze the following parameters over time: root area, convex hull area, total length, and the maximum root system width (**Figure 6** and **Supplementary Figures S3A–C**). Additionally, our system allowed us to analyze the angle from the seminal roots (SRGA) described by Oyanagi et al. (1993) and Manschadi et al. (2008) using ImageJ (**Supplementary Figure S3D**). The comparison of the root morphology of the spring barley trait GP and the facultative trait BCC93 shows a difference in the SRGA at 7 DAS (**Figure 6A**; **Supplementary Videos 1**, **2**). GP shows a broader angle (mean, 109.3°; n = 7) compared to BCC93 (mean, 62.7°; n = 4). This result underlines the robustness of our system, since the SRGA of BCC93 was previously measured with 68.66° (Jia et al., 2019).

The root tracking shows that the first root could be observed between 64 and 70 h (3 DAS), and the analyzed parameters resulted in highly reproducible results during early root development until 150 h (approximately 6 DAS) (**Supplementary Figure S3B**; **Figure 6B**; **Supplementary Videos 1**, **2**). We could observe a difference in the root and hull area and the total root length of the RSA analysis between GP and BCC93 (**Figure 6B**, B1-B3), but no difference in the max root width (**Figures 6B**, B4).

## 4 Discussion

Plant performance is strongly affected by environmental conditions. Subsequently, results of controlled conditions of the lab—”pampered inside, pestered outside”—are often not suitable to translate back to field conditions (Poorter et al., 2016): “Besides phenotypically differences between lab- and field-grown plants, the shoot and root environment and the effects of plant density must be considered.” Thus, the transfer of environmental conditions to controlled lab conditions will obviously improve the knowledge translation gained under lab conditions back to nature.

In nature, roots are growing below the surface in soil. Thus, our first step was the recording of environmental parameters in the field to transfer these parameters to controlled lab conditions. Since our research focus is on germination, early root, and grain development of spring barley, we have been measuring soil water content, soil moisture, soil temperature, and air temperature in natural fields where spring barley is sown. As we have recorded data for 3 years (Dermendjiev et al., 2021 and 2021, 2022), our data are quite robust and allowed us to set and perform germination and early root development experiments at environmental conditions as near as possible to nature. This includes considering parameters as air and soil temperature and moisture and light intensity.

Roots develop hidden underground in the dark and are only illuminated by the light that penetrates the first 10 mm of the soil (Tester and Morris, 1987). Subsequently, experimental conditions in the lab, where roots are often exposure to light, interfere with the natural root growth development and should be avoided. Within the past decade, RSA traits have been assessed in the lab non-invasively by 2D and 3D imaging techniques (Heeraman et al., 1997; Tracy et al., 2010; Zhu et al., 2011). 3D imaging techniques such as X-ray computed tomography and magnetic resonance imaging have been used to overcome the low spatial resolution often associated with 2D imaging. Whole-plant phenotyping is enabled by phenotyping platforms that allow simultaneous measuring of roots and shoots (Nagel et al., 2012; Jansen et al., 2014). However, high costs of 3D systems (Zhu et al., 2011) and phenotyping platforms remain.

Rhizoboxes are efficient tools for 2D RSA analysis. They enable root development analysis under natural environmental conditions considering parameters like the substrate (e.g., soil), the temperature and moisture gradient in the soil, the nutrient availability, and the microbiome. Since the first rhizobox, invented in 2008, the construction of rhizoboxes has been optimized, and their flexible construction allows the RSA analysis of many different plants, from crops to *Prunus* spp. seedlings (Lind et al., 2014; Figueroa-Bustos et al., 2018; Schmidt et al., 2018; Jia et al., 2019; Lesmes-Vesga et al., 2022).

We showed that our BIBLOX can be used as an effective DRD for proteomic analysis, since our proteomic data confirm already published effects of light on roots, e.g., on cytoskeleton proteins (Dyachok et al., 2011; Du et al., 2020; Halat et al., 2020; Cabrera et al., 2022) and on ROS (Yokawa et al., 2011). Additionally, first approaches to whole-plant phenotyping showed the effect of root illumination on shoot development. Our data therefore reinforces the importance of the application of field conditions to the lab and the value of our novel device, the BIBLOX. We want to point out, that our BIBLOX impresses with its structural flexibility. First, the dimensions of the rhizobox are easily adaptable if necessary, using the 3D-CAD software. Subsequently, a broad range of reusable rhizoboxes with different dimensions can be produced very quickly and independently, especially with an in-house 3D printer. The 2-mm thick glass can be ordered at a local glazier’s workshop or can be bought at a superstore and cut into pieces with a glass cutter. The glass is stably positioned in the rhizobox via the integrated rail; subsequently, no tools are necessary for the assembly of the rhizobox. For post-experiments, the glass can be removed smoothly via the rail without any shoot/root damage, subsequently enabling tissue processing for, e.g., -omics analysis. The used material PLA is very light and resistant to moisture, humidity, and temperatures. Additionally, the rhizobox is washable and subsequently reusable. Furthermore, since the 3D printing system enables the production of rhizoboxes of different sizes, the demand on the “housing” of these rhizoboxes was flexibility. The usage of LEGO® bricks, together with the software Studio 2.0 BrickLink Studio, allows an easy construction and production of BIBLOXes of different sizes (**Supplementary Data 3**). Once build, the LEGO® BIBLOX is more stable and thus more easily relocatable compared to housing systems using other materials like recycled cartons. We further want to point out the reproducibility of this system, since all components of the BIBLOX can computer-aided designed in advance and subsequently mounted in a consistent way. Within this manuscript, we described the BIBLOX by using opaque, black bricks. The BIBLOX can also be built with other colored bricks for root growth analysis. However, we recommend the usage of black bricks for root tracking analysis to reduce reflection issues. If necessary, opaque LEGO® bricks, which are composed of acrylonitrile-butadiene-styrene block copolymer (ABS), can be cleaned by warm soap water (also washable in washing machine) and sterilized with ethanol (Lind et al., 2014). Thus, when one set of LEGO® bricks is available, they can be reused for the construction of houses of different sizes. In addition to this flexibility, using a 3D-printing system for the production of rhizoboxes and the usage of (secondhand) LEGO® bricks to build the houses allows to construct an inexpensive DRD. Finally, we emphasized on setting up experiments that include more sustainable research to reduce the anthropogenic climate change. We used secondhand LEGO® bricks and produced 3D-printed rhizoboxes with bio-degradable materials. The usage of local and reusable material enables us to reduce the CO_2_ footprint in our lab. Of course, this is only the first step, and we have to optimize our experimental setup to further reduce our CO_2_ footprint to a minimum.

**Figure 7:**
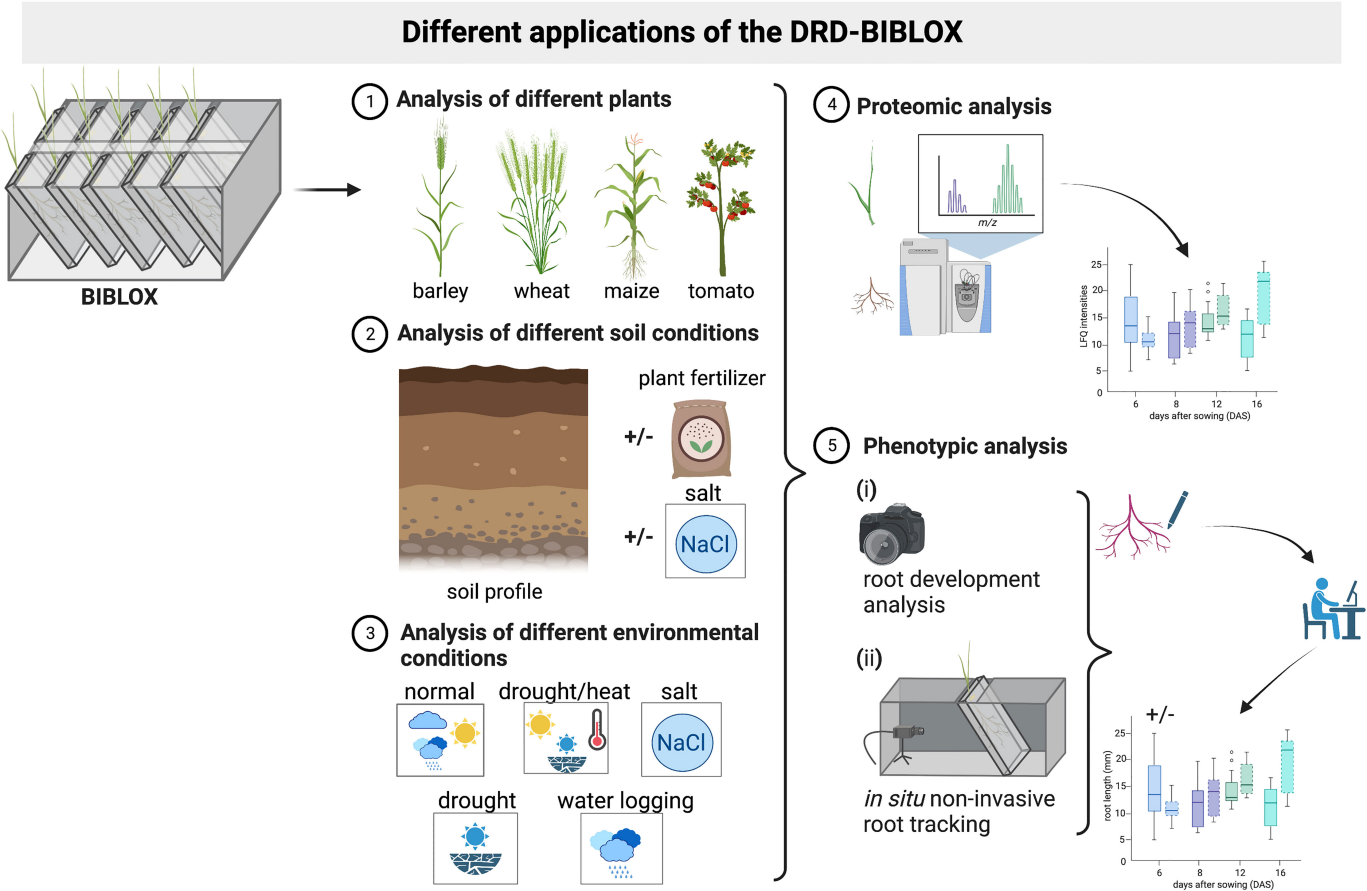
Schematic representation of the different applications of the BIBLOX. The BIBLOX can be used to (1) analyze a variety of different plants, (2) it can be utilized using different soil profiles, and (3) a variety of different environmental conditions are applicable. (4) Furthermore, the BIBLOX can be used as a plant sampling tool for molecular experiments like proteomic analysis; additionally, (5) phenotypical analyses are possible *via* a root tracking BIBLOX setup. Schema is illustrated @Biorender.

Our focus was to develop a DRD that is especially suitable for the analysis of specific and focused molecular biology-related investigations (e.g., reverse and forward genetic approaches) and for analyses of the RSA in response to distinct environmental factors including different substrate composition. Our setup allows follow-up molecular, biochemical, -omics, and physiological approaches of different crops (**Figure 7**). Since we used LEGO® bricks, our bench-top BIBLOX is flexible regarding its size and is easily relocatable. This flexibility will be extremely helpful for future experiments to investigate and adapt soil temperature and moisture descent-gradient environment for the root (González-García et al., 2022).

## 5 Conclusion

Here, we present our open-hardware tool, the DRD-BIBLOX, which is an inexpensive, very flexible, temperature- and humidity-resistant DRD, which allows barley germination and root development in soil, in the dark, with applied environmental parameters mirroring natural environmental conditions (soil temperature, air temperature, and soil moisture). Finally, the BIBLOX provides an imaging application for Dark-Root tracking controlled by a Raspberry Pi that enables an easy-to-use, reproducible, inexpensive, and a non-invasive RSA phenotyping approach. Recapitulating, the BIBLOX is a novel system with several striking features that allows non-invasive *in situ* early root tracking of several crops under controlled environmental conditions as near as possible to nature while being as accessible as possible and sustainable.

## Data availability statement

The original contributions presented in the study are included in the article/**Supplementary Material**. Further inquiries can be directed to the corresponding author.

## Author contributions

VI, GD, JJ, and MS designed the experiment. GD, ES, SP, and MS wrote the scripts. ES designed and fabricated the 3D-printed rhizobox and performed the image analyses. GD designed the non-invasive root tracking experiment. VI traced the images and performed the SRGA analyses. MS conducted the biological growth analysis in the BIBLOX. TN, GM, and DL performed the LC-MS/MS experiments. MS performed the data analysis. AT harvested, analyzed the bio-organic soil, and performed the setup of the soils as near as possible to nature. SP, MS, and VI mounted the data sensors and loggers in the field. SP analyzed the data logger. VI and MS wrote the manuscript. All authors contributed to manuscript preparation and editing. All authors contributed to the article and approved the submitted version.

## Supplementary material

Supplementary Figure 1 Set up of the field experiment in a barley field, recording environmental parameters. **(A)** A schematic presentation of the field and a picture taken via a drone are showing the position of the sensors within the field in lower Austria. **(B)** The recordings of the sensors of the soil temperature and soil moisture in -20 cm depth, the PAR values, and the air temperature. For the soil temperature and moisture only two sensors are presented due to missing measurements of the third sensor. The black line of the soil moisture graph indicates the mean value.

Supplementary Figure 2 Virtual constructions of LEGO® BIBLOXes, computer-aided designed via Studio 2.0 BrickLink Studio software, suitable for different applications. **(A)** A single BIBLOX for root-tracking experiments in dark. One rhizobox can be inserted, including space for camera equipment. **(B)** A triple BIBLOX, for root-tracking experiments. Three rhizoboxes can be inserted, including space for camera equipment. **(C)** A triple BIBLOX, for root-tracking experiments in dark and light. Three rhizoboxes can be inserted (two in dark, one in light), including space for camera equipment. **(D)** A BIBLOX for sampling approaches with roots in dark. In this setup the BIBLOX can fit up to 14 rhizoboxes. **(E)** A LEGO® scaffold for experiments with light exposed roots, setup can fit up to 6 rhizoboxes. All the constructions can be altered according to individual needs.

Supplementary Figure 3 Gallery of images taken between 15 hours and 230 hours after sowing (HAS) of barley (GP) DGRs in the BIBLOX root-tracking device at 14 °C/12 °C day/night cycle in a plant growth chamber (**Supplemental Video 1**). **(A)** Series of images, 15 hours interval. **(B)** The same images as in **(A)** traced manually to enhance the contrast between the root and the soil for further semi-automatic analyses. **(C)** Representative output from the image analysis at 76 hours (76 HAS), 152 hours (152 HAS) and 230 hours (230 HAS) after sowing (HAS). The blue line is the maximum root system width and height, the green line is the convex hull, and the red shows the analyzed roots. **(D)** Representative image of the SRGA calculation. The sum of all measured angles, root growth angle left (RGAl), root growth angle right (RGAr) and SRGA is 180°. Scale equals 0.5 cm. Plants were grown in Cocopeat_black, which refers to Cocopeat including activated charcoal. PVC rhizoboxes were used.

Supplementary Figure 4 PCA and loading blot of proteins of 8 DAS. **(A)** PC1 (68.7%) separates the proteins of shoots and roots, whereas PC2 (17.6%) separates the proteins depending on the illumination of the root. **(B)** The loading plot indicates the proteins (UniProt reference numbers) that contribute most to the distribution in PC1 and PC2. **(C)** The contribution plots for PC1 (C1, C2) and PC2 (C3, C4) show how much the total number of proteins (2158) (C1, C3) and the top 30 proteins (C2, C4) contribute to the distribution in PC1 and PC2 respectively. The y axis shows the extent of contribution in %, and the x axis shows the number of the different proteins according to **Supplemental Table 4**.

Supplementary Figure 5 PCA and loading blot of proteins of 16 DAS. **(A)** PC1 (72.5%) separates the proteins of shoots and roots, whereas PC2 (15.5%) separates the proteins depending on the illumination of the root. **(B)** The loading plot indicates the proteins (UniProt reference numbers) that contribute most to the distribution in PC1 and PC2. **(C)** The contribution plots for PC1 (C1, C2) and PC2 (C3, C4) show how much the total number of proteins (2158) (C1, C3) and the top 30 proteins (C2, C4) contribute to the distribution in PC1 and PC2 respectively. The y axis shows the extent of contribution in %, and the x axis shows the number of the different proteins according to **Supplemental Table 4**.

Supplementary Figure 6 PCA and loading blot of proteins of 8 and 16 DAS. **(A)** PC1 (53.5%) separates the proteins of shoots and roots, whereas PC2 (13.7%) separates the proteins depending on the illumination of the root. **(B)** The loading plot indicates the proteins (UniProt reference numbers) that contribute most to the distribution in PC1 and PC2. **(C)** The contribution plots for PC1 (C1, C2) and PC2 (C3, C4) show how much the total number of proteins (2158) (C1, C3) and the top 30 proteins (C2, C4) contribute to the distribution in PC1 and PC2 respectively. The y axis shows the extent of contribution in %, and the x axis shows the number of the different proteins according to **Supplemental Table 4**.

Supplementary Figure 7 Test arrangement of several BIBLOXes in one growth chamber. During running D-Root experiments the BIBLOXes are covered with a lid out of LEGO® plates. Note the experiment in the middle where the roots are not protected from light.

Supplementary Data Sheet 1 R-script for data logger analysis of the recorded environmental parameters.

Supplementary Data Sheet 2 3D-models and the corresponding file of the constructed rhizobox.

Supplementary Data Sheet 3 Studio 2.0 BrickLink Studio software file of the constructed BIBLOXes es of different sizes and purposes. The dataset includes a single BIBLOX for root tracking in dark, a triple BIBLOX for root tracking in dark and light, a triple BIBLOX for root tracking in dark, a BIBLOX for sampling in dark holding up to 14 rhizoboxes and a scaffold for 6 rhizoboxes for experiments with root light exposure.

Supplementary Data Sheet 4 Official AGES report of the tested bio-organic- and conventional field soil.

Supplementary Data Sheet 5 R-script used for analysis of proteomic data and graphic representation of results.

Supplementary Table 1 Data of the environmental parameters recorded in the field experiment in year 2021.

Supplementary Table 2 The ID numbers and the quantity of all used LEGO® bricks to set up the different BIBLOXes. The corresponding rendered BIBLOXes are available in **Supplemental Data 3**.

Supplementary Table 3 Excel file of results and mathematical analysis of the proteomic data.

Supplementary Table 4 Excel file including extracts of **Supplemental Table 3**, referred to in R script as pca_r_try_29.12.2022.xlsx. Data for PCA plots.

Supplementary Video 1 Root tracking of the barley trait GP. Pictures were taken every 4 minutes for a period of 230 hours after sowing.

Supplementary Video 2 Root tracking of the barley trait BCC93. Pictures were taken every 4 minutes for a period of 192 hours after sowing.
