# Supplementary figures and images for: A bench-top Dark-Root device built with LEGO® bricks enables a non-invasive plant root development analysis in soil conditions mirroring nature
Source: Front Plant Sci. 2023 May 31;14:1166511. doi: 10.3389/fpls.2023.1166511 (PMC10264708; doi:10.3389/fpls.2023.1166511)

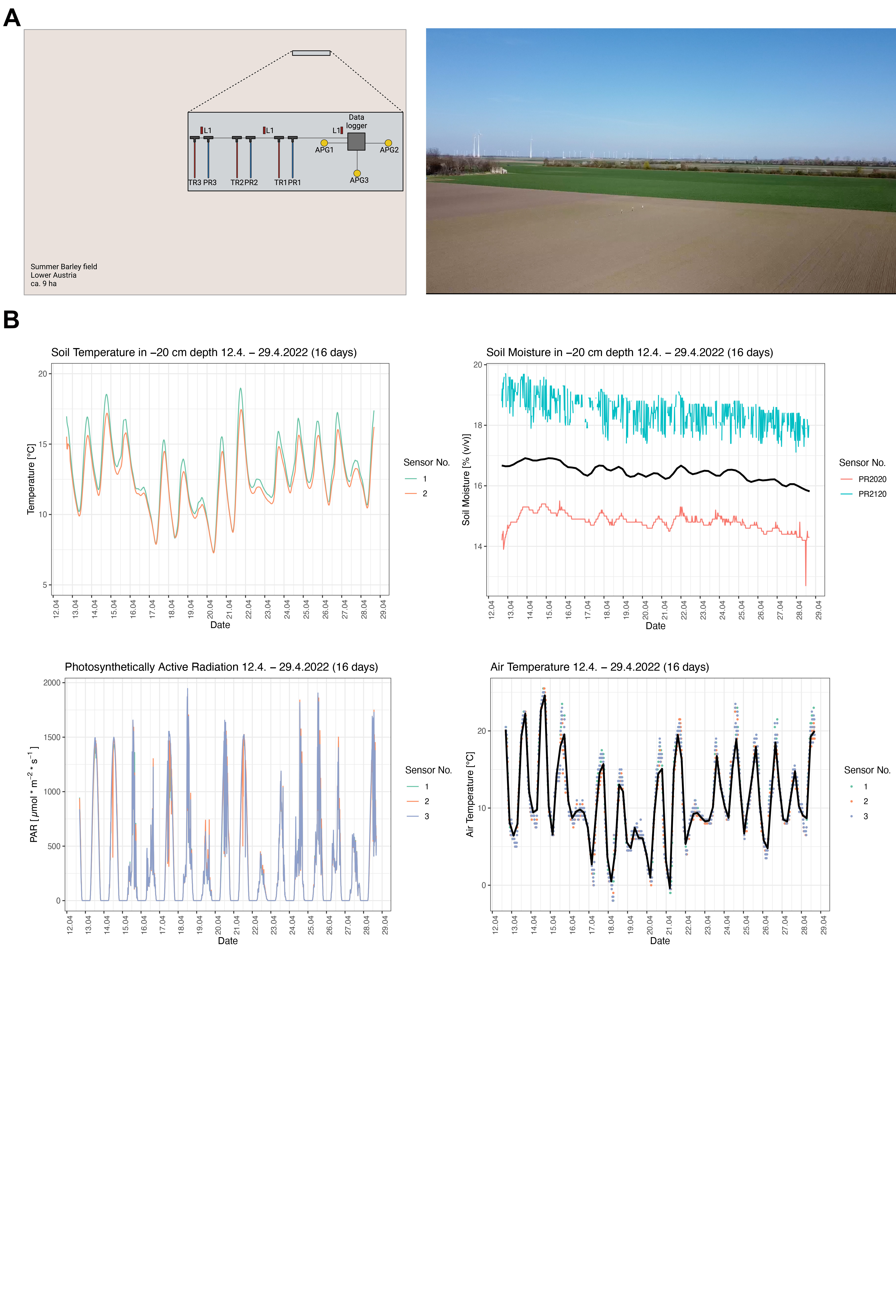

Supplement: Supplementary Figure 1 — Set up of the field experiment in a barley field, recording environmental parameters. (A) A schematic presentation of the field and a picture taken via a drone are showing the position of the sensors within the field in lower Austria. (B) The recordings of the sensors of the soil temperature and soil moisture in -20 cm depth, the PAR values, and the air temperature. For the soil temperature and moisture only two sensors are presented due to missing measurements of the third sensor. The black line of the soil moisture graph indicates the mean value. [file Image_1.jpeg]

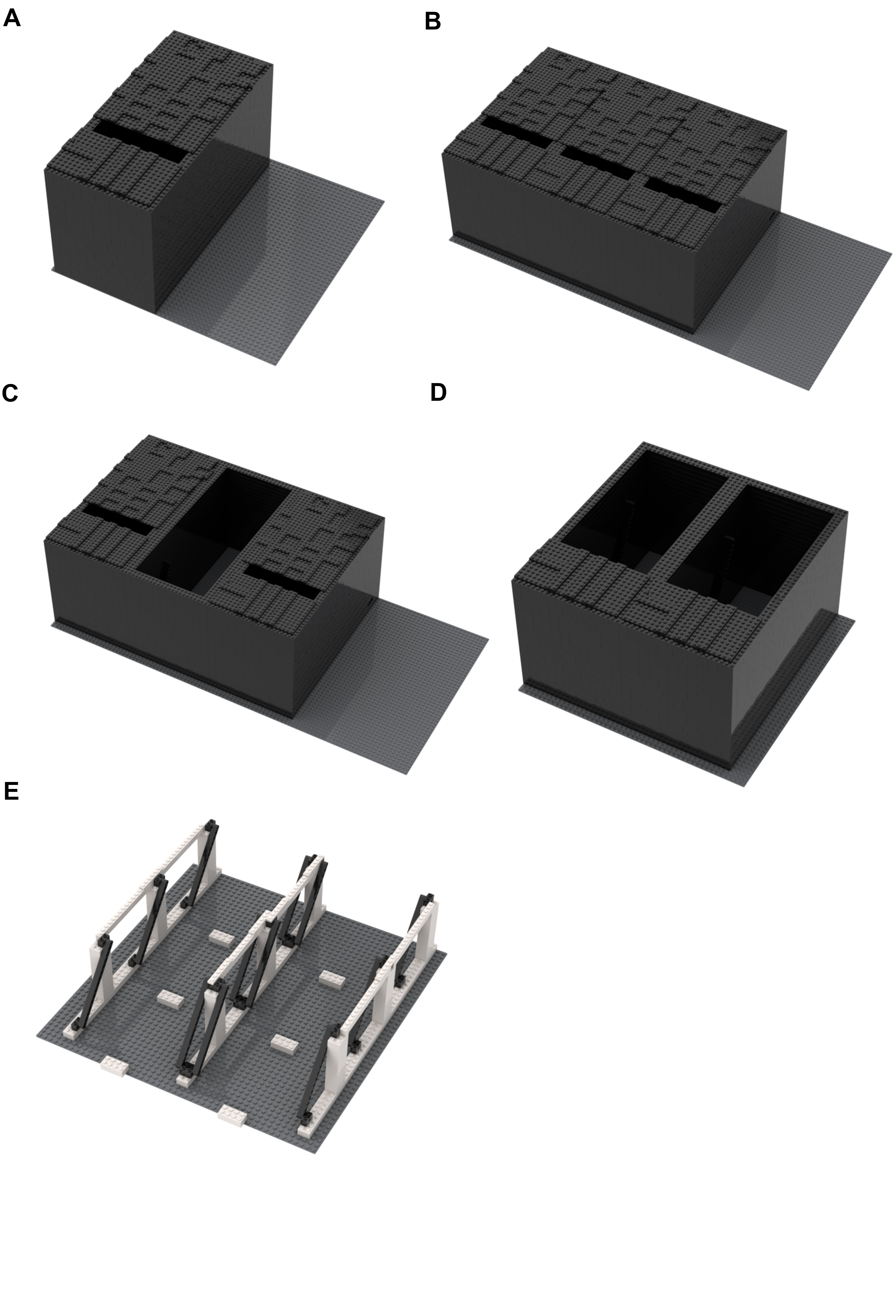

Supplement: Supplementary Figure 2 — Virtual constructions of LEGO® BIBLOXes, computer-aided designed via Studio 2.0 BrickLink Studio software, suitable for different applications. (A) A single BIBLOX for root-tracking experiments in dark. One rhizobox can be inserted, including space for camera equipment. (B) A triple BIBLOX, for root-tracking experiments. Three rhizoboxes can be inserted, including space for camera equipment. (C) A triple BIBLOX, for root-tracking experiments in dark and light. Three rhizoboxes can be inserted (two in dark, one in light), including space for camera equipment. (D) A BIBLOX for sampling approaches with roots in dark. In this setup the BIBLOX can fit up to 14 rhizoboxes. (E) A LEGO® scaffold for experiments with light exposed roots, setup can fit up to 6 rhizoboxes. All the constructions can be altered according to individual needs. [file Image_2.jpeg]

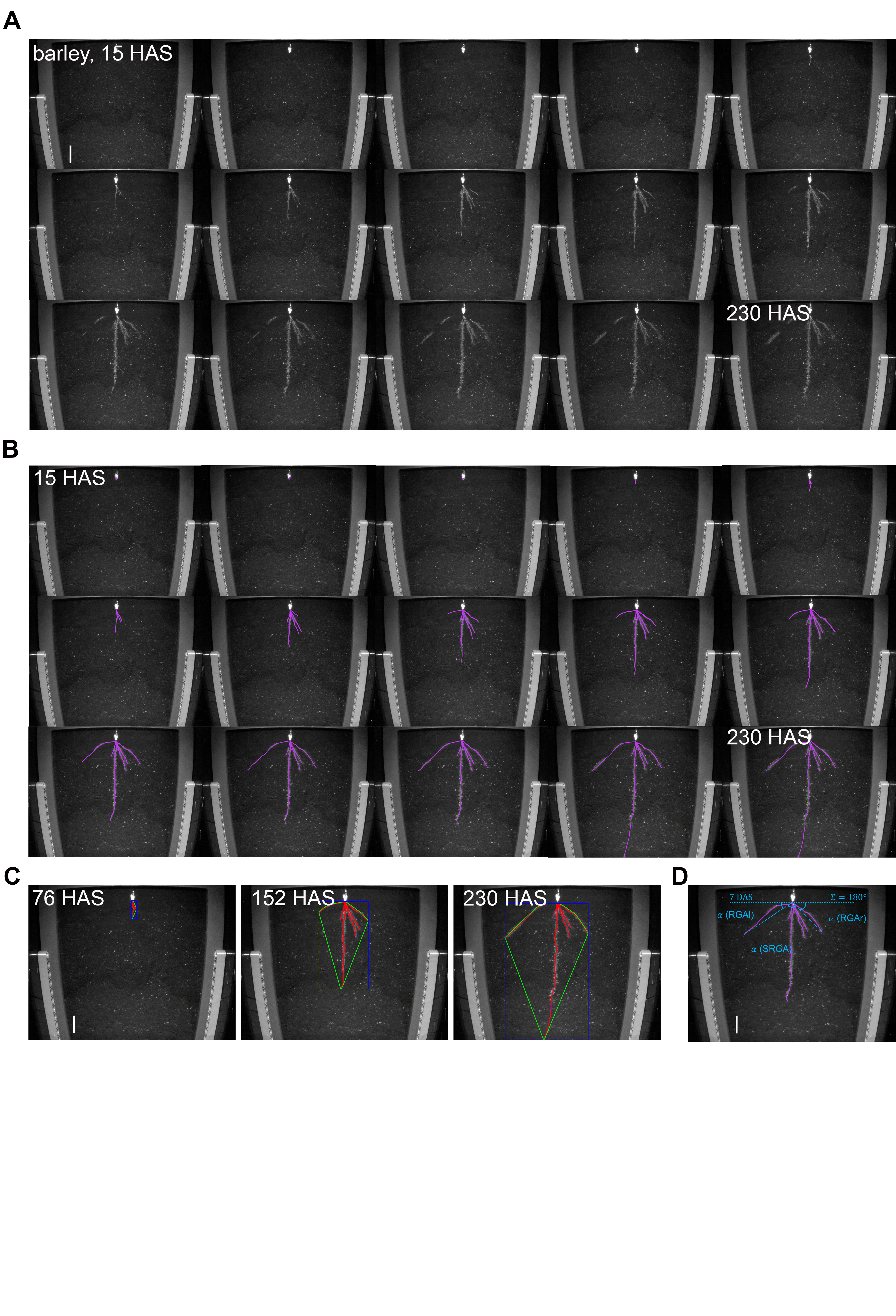

Supplement: Supplementary Figure 3 — Gallery of images taken between 15 hours and 230 hours after sowing (HAS) of barley (GP) DGRs in the BIBLOX root-tracking device at 14 °C/12 °C day/night cycle in a plant growth chamber (Supplemental Video 1). (A) Series of images, 15 hours interval. (B) The same images as in (A) traced manually to enhance the contrast between the root and the soil for further semi-automatic analyses. (C) Representative output from the image analysis at 76 hours (76 HAS), 152 hours (152 HAS) and 230 hours (230 HAS) after sowing (HAS). The blue line is the maximum root system width and height, the green line is the convex hull, and the red shows the analyzed roots. (D) Representative image of the SRGA calculation. The sum of all measured angles, root growth angle left (RGAl), root growth angle right (RGAr) and SRGA is 180°. Scale equals 0.5 cm. Plants were grown in Cocopeat_black, which refers to Cocopeat including activated charcoal. PVC rhizoboxes were used. [file Image_3.jpeg]

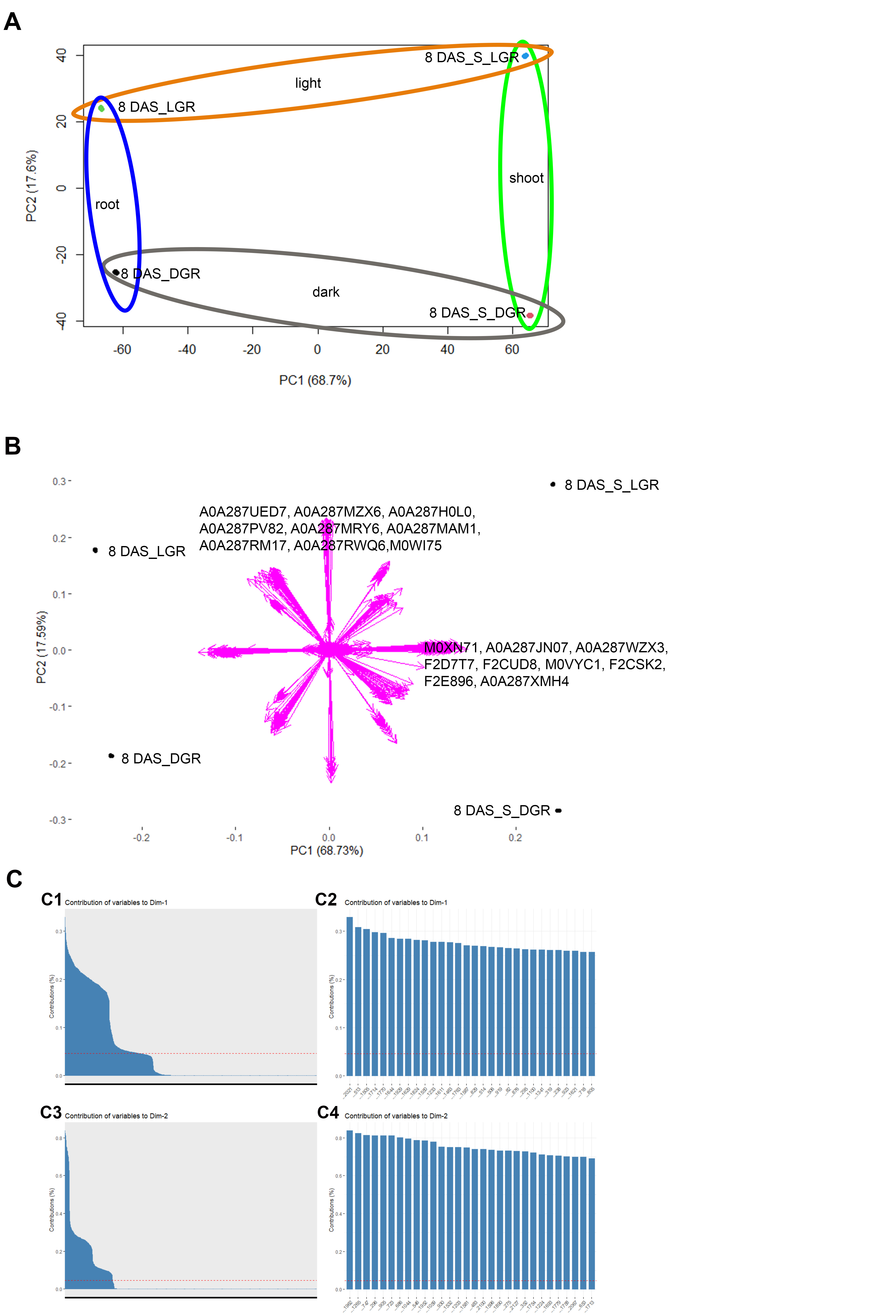

Supplement: Supplementary Figure 4 — PCA and loading blot of proteins of 8 DAS. (A) PC1 (68.7%) separates the proteins of shoots and roots, whereas PC2 (17.6%) separates the proteins depending on the illumination of the root. (B) The loading plot indicates the proteins (UniProt reference numbers) that contribute most to the distribution in PC1 and PC2. (C) The contribution plots for PC1 (C1, C2) and PC2 (C3, C4) show how much the total number of proteins (2158) (C1, C3) and the top 30 proteins (C2, C4) contribute to the distribution in PC1 and PC2 respectively. The y axis shows the extent of contribution in %, and the x axis shows the number of the different proteins according to Supplemental Table 4. [file Image_4.jpeg]

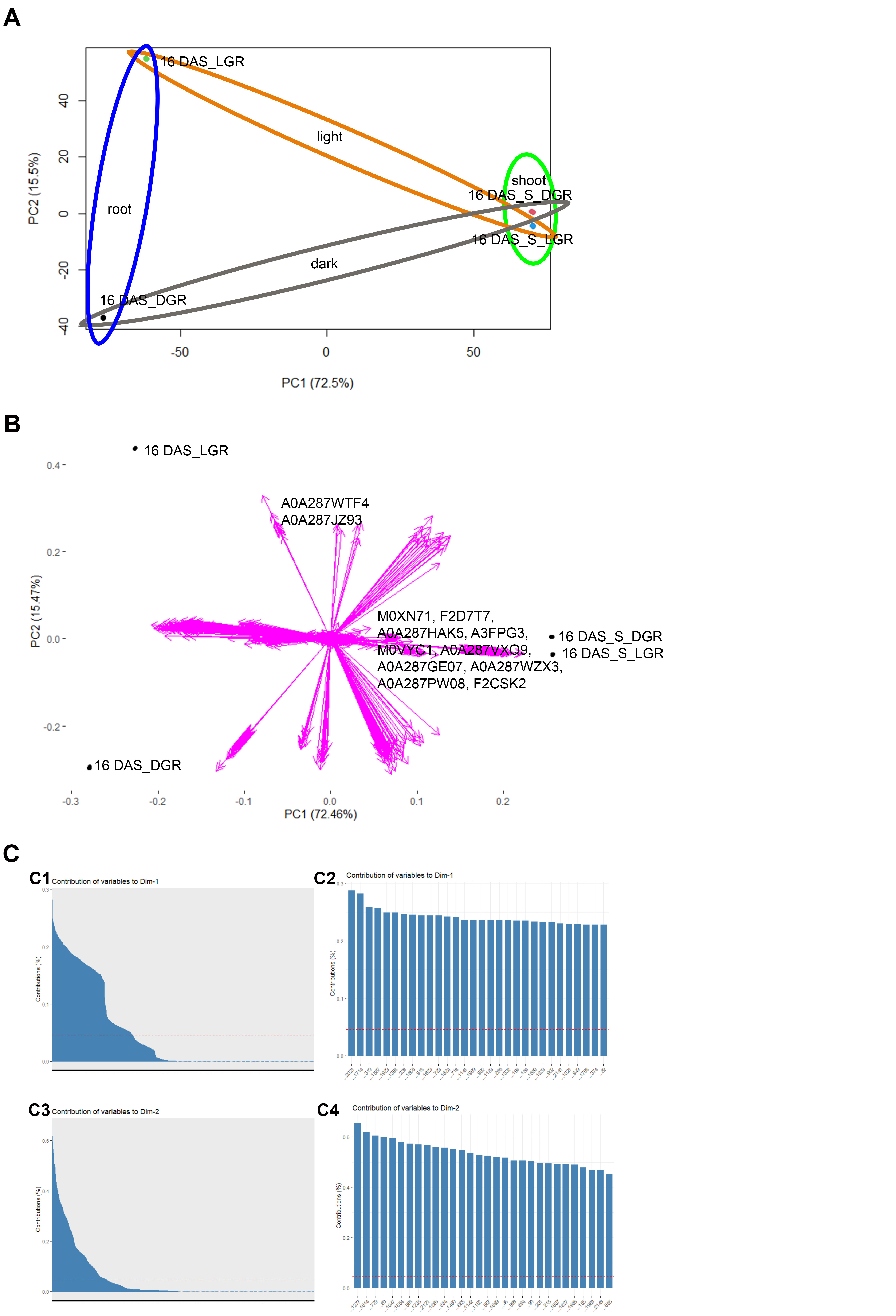

Supplement: Supplementary Figure 5 — PCA and loading blot of proteins of 16 DAS. (A) PC1 (72.5%) separates the proteins of shoots and roots, whereas PC2 (15.5%) separates the proteins depending on the illumination of the root. (B) The loading plot indicates the proteins (UniProt reference numbers) that contribute most to the distribution in PC1 and PC2. (C) The contribution plots for PC1 (C1, C2) and PC2 (C3, C4) show how much the total number of proteins (2158) (C1, C3) and the top 30 proteins (C2, C4) contribute to the distribution in PC1 and PC2 respectively. The y axis shows the extent of contribution in %, and the x axis shows the number of the different proteins according to Supplemental Table 4. [file Image_5.jpeg]

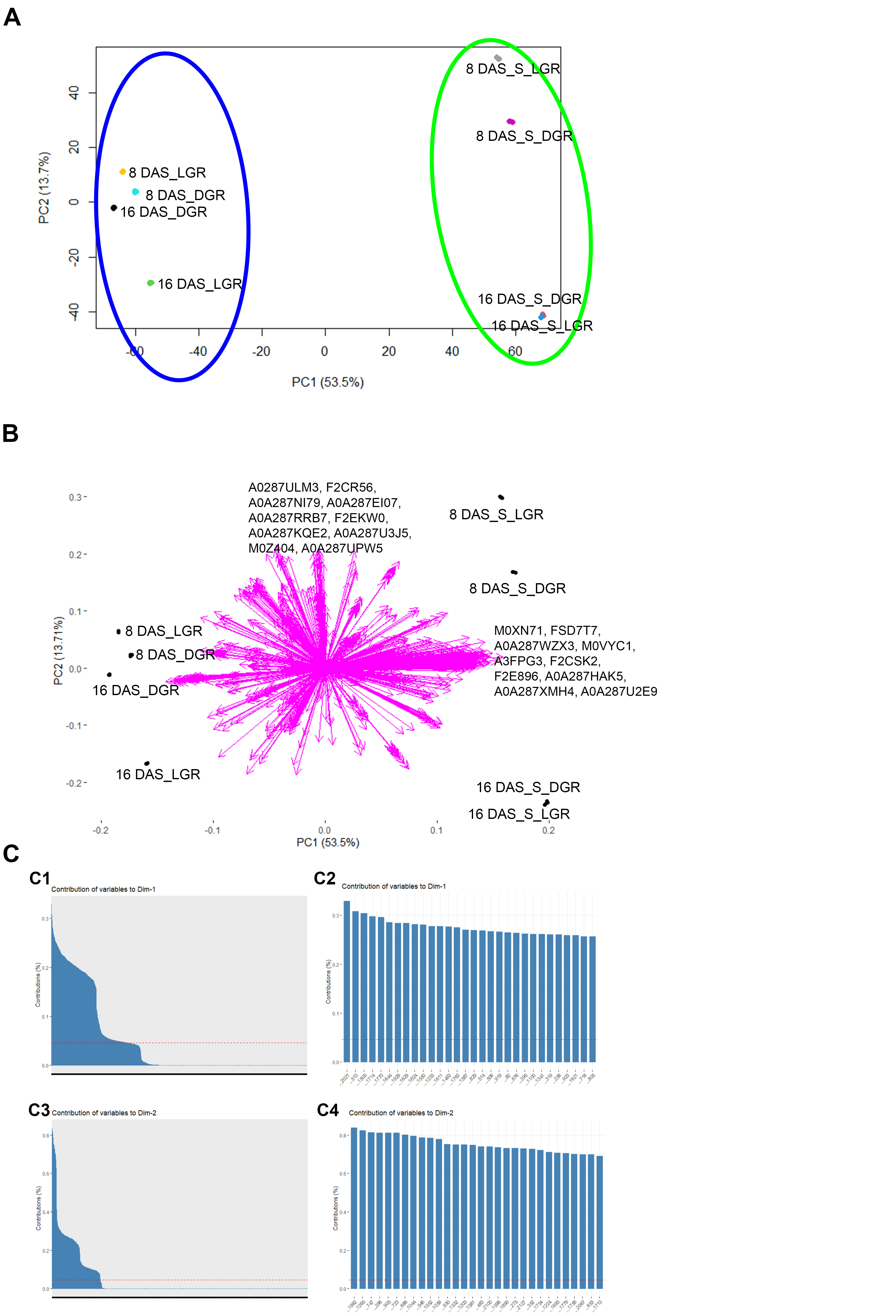

Supplement: Supplementary Figure 6 — PCA and loading blot of proteins of 8 and 16 DAS. (A) PC1 (53.5%) separates the proteins of shoots and roots, whereas PC2 (13.7%) separates the proteins depending on the illumination of the root. (B) The loading plot indicates the proteins (UniProt reference numbers) that contribute most to the distribution in PC1 and PC2. (C) The contribution plots for PC1 (C1, C2) and PC2 (C3, C4) show how much the total number of proteins (2158) (C1, C3) and the top 30 proteins (C2, C4) contribute to the distribution in PC1 and PC2 respectively. The y axis shows the extent of contribution in %, and the x axis shows the number of the different proteins according to Supplemental Table 4. [file Image_6.jpeg]

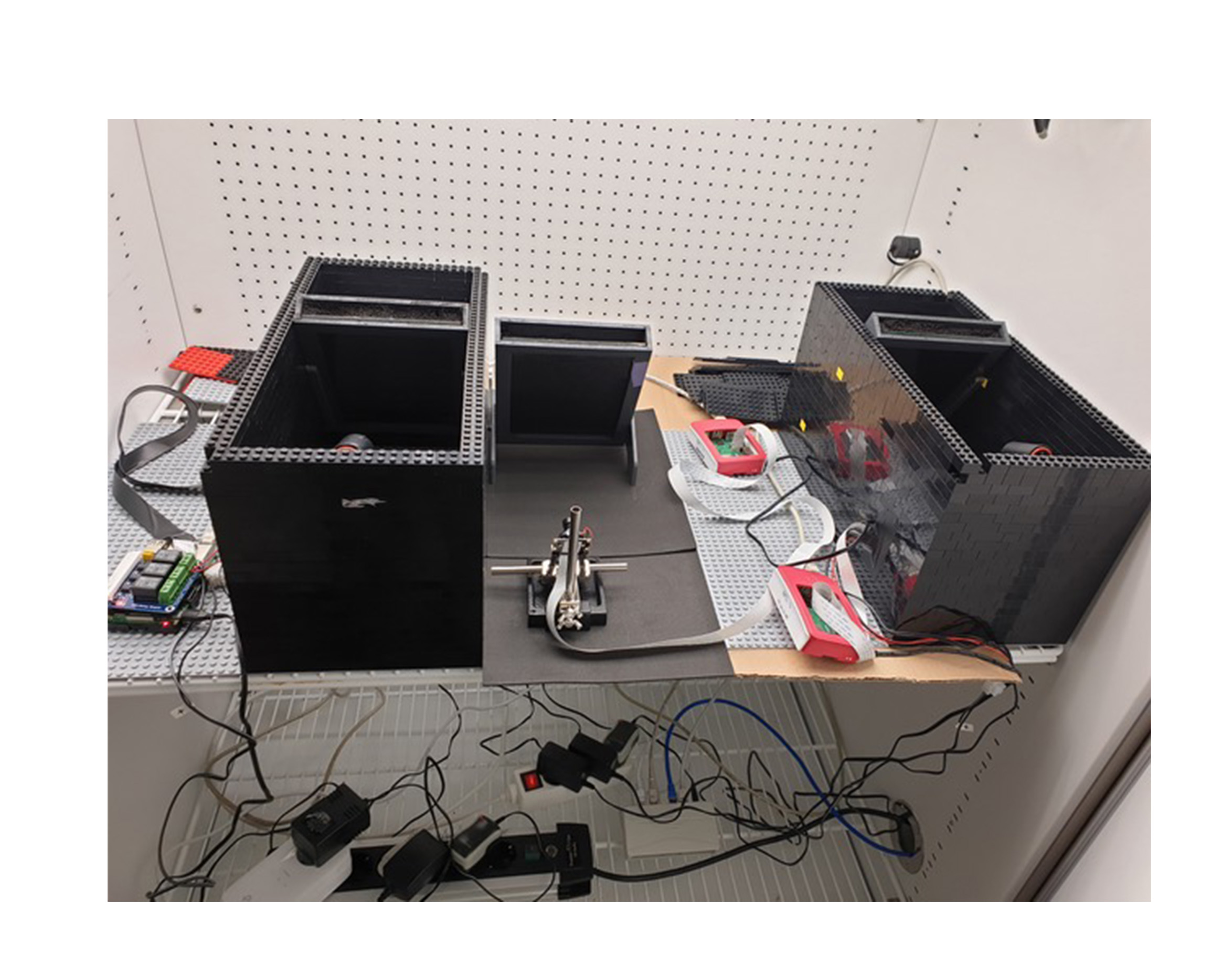

Supplement: Supplementary Figure 7 — Test arrangement of several BIBLOXes in one growth chamber. During running D-Root experiments the BIBLOXes are covered with a lid out of LEGO® plates. Note the experiment in the middle where the roots are not protected from light. [file Image_7.jpeg]
