# Supplementary material for: A bench-top Dark-Root device built with LEGO® bricks enables a non-invasive plant root development analysis in soil conditions mirroring nature
Source: Front Plant Sci. 2023 May 31;14:1166511. doi: 10.3389/fpls.2023.1166511 (PMC10264708; doi:10.3389/fpls.2023.1166511)
Supplement: Supplementary Data Sheet 1 — R-script for data logger analysis of the recorded environmental parameters. [file DataSheet_1.pdf]

```r
# Setup ------------------------------------------------------------------------
# libraries
library(ggplot2)
library(RColorBrewer)
library(reshape2)
library(lubridate)
library(scales)

# set wd
wd <- ("ENTER WD")
setwd(wd)

# setting a color palette for plotting
colpal <- brewer.pal(3, "Set2")

# Data preparation yDoc ------------------------------------------------------------
# importing the yDoc Data
yDoc <- read.csv("2022_field.csv", skip = 2)

# setting date column date format
yDoc[, 1] <- as.Date(yDoc[, 1])

# adding a Date - Time column with correct format
yDoc <- cbind(yDoc, DateTime = paste(yDoc[, 1], yDoc[, 2], sep = " "))
yDoc$DateTime <- as.POSIXct(yDoc$DateTime)

# remove all rows where no data was recorded.
yDoc_data <- yDoc[-which(is.na(yDoc$AVGVi)), ]

# Names of Sensors
PAR_sensors  <- c("PAR1", "PAR2", "PAR3")
ST20_sensors <- c("STa20", "STb20", "STc20")
ST30_sensors <- c("STa30", "STb30", "STc30")
ST50_sensors <- c("STb50", "STc50") # STa50 did not measure!
PR10_sensors <- c("PR2010", "PR2110", "PR2210")
PR20_sensors <- c("PR2020", "PR2120", "PR2220")
PR30_sensors <- c("PR2030", "PR2130", "PR2230")
PR40_sensors <- c("PR2040", "PR2140", "PR2240")
temp_sensors <- c("STa20", "STa30", # again left out STa50 here
                  "STb20", "STb30", "STb50",
                  "STc20", "STc30", "STc50",
                  "mean_ST20", "mean_ST30", "mean_ST50")
moist_sensors <- c("PR2010", "PR2020", "PR2030", "PR2040",
                   "PR2110", "PR2120", "PR2130", "PR2140",
                   "PR2210", "PR2220", "PR2230", "PR2240",
                   "mean_PR10", "mean_PR20", "mean_PR30", "mean_PR40")

# calculate means & standard deviations
# Means
mean_PAR  <- rowMeans(yDoc_data[, PAR_sensors],  na.rm = TRUE)
mean_ST20 <- rowMeans(yDoc_data[, ST20_sensors], na.rm = TRUE)
mean_ST30 <- rowMeans(yDoc_data[, ST30_sensors], na.rm = TRUE)
mean_ST50 <- rowMeans(yDoc_data[, ST50_sensors], na.rm = TRUE)
mean_PR10 <- rowMeans(yDoc_data[, PR10_sensors], na.rm = TRUE)
mean_PR20 <- rowMeans(yDoc_data[, PR20_sensors], na.rm = TRUE)
mean_PR30 <- rowMeans(yDoc_data[, PR30_sensors], na.rm = TRUE)
mean_PR40 <- rowMeans(yDoc_data[, PR40_sensors], na.rm = TRUE)

# SDs
sdPAR  <- apply(yDoc_data[, PAR_sensors],  2, sd, na.rm = TRUE)
sdST20 <- apply(yDoc_data[, ST20_sensors], 2, sd, na.rm = TRUE)
sdST30 <- apply(yDoc_data[, ST30_sensors], 2, sd, na.rm = TRUE)
sdST50 <- apply(yDoc_data[, ST50_sensors], 2, sd, na.rm = TRUE)
sdPR10 <- apply(yDoc_data[, PR10_sensors], 2, sd, na.rm = TRUE)
sdPR20 <- apply(yDoc_data[, PR20_sensors], 2, sd, na.rm = TRUE)
sdPR30 <- apply(yDoc_data[, PR30_sensors], 2, sd, na.rm = TRUE)
sdPR40 <- apply(yDoc_data[, PR40_sensors], 2, sd, na.rm = TRUE)

# merge to a dataframe
yDoc_data_calc <- data.frame(yDoc_data, mean_PAR,
                             mean_ST20, mean_ST30, mean_ST50,
                             mean_PR10, mean_PR20, mean_PR30, mean_PR40,
                             sdPAR, sdST20, sdST30, sdST50,
```

```
                                sdPR10, sdPR20, sdPR30, sdPR40)

# setting measurement time borders
datetime_start <- yDoc_data$DateTime[1]
datetime_16d   <- yDoc_data$DateTime[1] + 16 * 60 * 60 * 24

# Subsetting data of first 16 days
subs_16 <- yDoc_data_calc[which(yDoc_data_calc$DateTime < datetime_16d), ]
# and for different sensors
subs_16_st  <- cbind(subs_16$DateTime, subs_16[, temp_sensors])
subs_16_pr  <- cbind(subs_16$DateTime, subs_16[, moist_sensors])
subs_16_par <- cbind(subs_16$DateTime, subs_16[, PAR_sensors])

colnames(subs_16_st)  <- c("DateTime", temp_sensors)
colnames(subs_16_pr)  <- c("DateTime", moist_sensors)
colnames(subs_16_par) <- c("DateTime", PAR_sensors)

# Reshaping from wide to long data format
subs_16_temp_long  <- melt(subs_16_st,  id.vars = "DateTime")
subs_16_moist_long <- melt(subs_16_pr,  id.vars = "DateTime")
subs_16_PAR_long   <- melt(subs_16_par, id.vars = "DateTime")

# subsetting different depths, the soil temperature measurements of 20 and 50 cm were switched in the yDoc sensor setup.

subs_16_temp20_long <-
  subs_16_temp_long[which(subs_16_temp_long$variable == ST50_sensors), ]

subs_16_sm20_long <-
  subs_16_moist_long[which(subs_16_moist_long$variable == c("PR2020",
                                                              "PR2120")), ]

# calculating soil temperature means for first 16 days in 20 cm depth --------
subs_16_meantemp20 <- cbind(subs_16$DateTime, subs_16$mean_ST50)
summary(subs_16_meantemp20[, 2])

# Data Preparation Air-Sensors -----------------------------------------------
# import  data
air1 <- read.csv("2022_field1.csv", sep = ",")
air2 <- read.csv("2022_field2.csv", sep = ",")
air3 <- read.csv("2022_field3.csv", sep = ",")

# date column as POSIX
air1$time1 <- as.POSIXct(air1$time1)
air2$time2 <- as.POSIXct(air2$time2)
air3$time3 <- as.POSIXct(air3$time3)

# merge air 2 and 3 (same length)
air <- data.frame(air2, air3)

# merge air 1 to this (shorter)
air <- merge(air1, air, by = "nr", all = TRUE)

# removing unnecessary cols
air <- subset(air, select = -c(sn1, sn2, sn3, nr.1))

# subsetting the first 16 days
air_subs16 <- air[which(air$time3 > datetime_start & air$time3 < datetime_16d), ]

# setting sensor names
airtemp_sensor_names <- c("celsius1",
                          "celsius2",
                          "celsius3")
airhum_sensor_names <- c("rh1",
                         "rh2",
                         "rh3")
airdp_sensor_names <- c("dewpoint1",
                        "dewpoint2",
                        "dewpoint3")

# calculating average temperature, humidity & dewpoint
mean_air_temp <- rowMeans(air_subs16[, airtemp_sensor_names])
mean_air_rh   <- rowMeans(air_subs16[, airhum_sensor_names])
```

```r
mean_air_dp   <- rowMeans(air_subs16[, airdp_sensor_names])

air_subs16 <- cbind(air_subs16, mean_air_temp, mean_air_rh, mean_air_dp)

# round minutes
t_round <- ceiling_date(air_subs16$time3, "minute")
air_subs16 <- cbind(air_subs16, t_round)

# subsetting the different measurement categories
airsens_subs_colnames <- c("time", "sensor1", "sensor2", "sensor3")

airtemp_subs16 <- air_subs16[, c("t_round", airtemp_sensor_names)]
colnames(airtemp_subs16) <- airsens_subs_colnames

airhum_subs16 <- air_subs16[, c("t_round", airhum_sensor_names)]
colnames(airhum_subs16) <- airsens_subs_colnames

airdp_subs16 <- air_subs16[, c("t_round", airdp_sensor_names)]
colnames(airdp_subs16) <- airsens_subs_colnames

mean_airtemphum_subs16 <- air_subs16[, c("t_round",
                                         "mean_air_temp",
                                         "mean_air_rh")]

colnames(mean_airtemphum_subs16) <- c("time", "temperature", "rh")

# convert into long format
airtemp_subs16_long <- melt(airtemp_subs16, id.vars = "time")
airhum_subs16_long  <- melt(airhum_subs16,  id.vars = "time")
airdp_subs16_long   <- melt(airdp_subs16,   id.vars = "time")

# Plotting ------------------------------------------------------------------
# Plotting data of all PAR Sensors
p_par <- ggplot(subs_16_PAR_long, aes(x = DateTime, y = value)) +
  geom_line(aes(colour = variable, group = variable)) +
  # geom_smooth(method = "loess", span = 0.05) +
  labs(title = "Photosynthetically Active Radiation 12.4. - 29.4.2022 (16 days)",
       x     = "Date",
       y = bquote("PAR ["~µmol~"*"~ m^-2~"*"~ s^-1~"]"),
       colour = "Sensor No.") +
  scale_x_datetime(date_labels = "%d.%m", date_breaks = "1 day") +
  scale_colour_manual(labels = c("1", "2", "3"), values = colpal) +
  theme_bw() +
  theme(axis.text.x = element_text(angle = 90))
p_par

# plotting soil temperature at 20 cm
p_soiltemp20 <- ggplot(subs_16_temp20_long, aes(x = DateTime, y = value)) +
  geom_line(aes(colour = variable, group = variable)) +
  labs(title = "Soil Temperature in 20 cm Depth 12.4. - 29.4.2022 (16 days)",
       x     = "Date",
       y     = "Temperature [°C]",
       colour = "Sensor No.") +
  ylim(5, 20) +
  scale_x_datetime(date_labels = "%d.%m", date_breaks = "1 day") +
  scale_colour_manual(labels = c("1", "2"), values = colpal) +
  theme_bw() +
  theme(axis.text.x = element_text(angle = 90))
p_soiltemp20

# plotting soil moisture at 20 cm
p_soilmoist20 <- ggplot(subs_16_sm20_long, aes(x = DateTime, y = value)) +
  geom_line(aes(colour = variable, group = variable)) +
  labs(title = "Soil moisture in 20 cm depth 12.4. - 29.4.2022 (16 days)",
       x     = "Date",
       y     = "Soil Moisture [% (v/v)]",
       colour = "Sensor No.") +
  scale_x_datetime(date_labels = "%d.%m", date_breaks = "1 day") +
  theme_bw() +
  theme(axis.text.x = element_text(angle = 90)) +
  geom_smooth(method = "loess", span = 0.1, colour = "black")
p_soilmoist20
```

```r
# plotting air temperature
p_airtemp <- ggplot(airtemp_subs16_long, aes(x = time, y = value)) +
  geom_point(size = 0.6, aes(colour = variable, group = variable)) +
  labs(title  = "Air Temperature 12.4. - 29.4.2022 (16 days)",
       x      = "Date",
       y      = "Air Temperature [°C]",
       colour = "Sensor No." ) +
  scale_x_datetime(date_labels = "%d.%m", date_breaks = "1 day") +
  scale_colour_manual(labels = c("1", "2", "3"), values = colpal) +
  theme_bw() +
  theme(axis.text.x = element_text(angle = 90)) +
  geom_smooth(method = "loess", span = 0.01, colour = "black", se = FALSE)
p_airtemp

# plotting air humidity
p_airhum <- ggplot(airhum_subs16_long, aes(x = time, y = value)) +
  geom_point(size = 0.6, aes(colour = variable, group = variable)) +
  labs(title = "Air Humidity 12.4. - 29.4.2022 (16 days)",
       x     = "Date",
       y     = "Relative Air Humidity [%]",
       colour = "Sensor No." ) +
  scale_x_datetime(date_labels = "%d.%m", date_breaks = "1 day") +
  scale_colour_manual(labels = c("1", "2", "3"), values = colpal) +
  theme_bw() +
  theme(axis.text.x = element_text(angle = 90)) +
  geom_smooth(method = "loess", span = 0.01, colour = "black", se = FALSE)
p_airhum
```
