# Supplementary material for: A bench-top Dark-Root device built with LEGO® bricks enables a non-invasive plant root development analysis in soil conditions mirroring nature
Source: Front Plant Sci. 2023 May 31;14:1166511. doi: 10.3389/fpls.2023.1166511 (PMC10264708; doi:10.3389/fpls.2023.1166511)
Supplement: Supplementary Data Sheet 4 — Official AGES report of the tested bio-organic- and conventional field soil. [file DataSheet_4.pdf]

Institut für Nachhaltige Pflanzenproduktion
Spargelfeldstraße 191, 1220 Wien
Head of Institute: Mag. Bernhard Föger

Thür Robert
Reitzenschlag 15
3874 Litschau

Date: 29.03.2023
Contact: DI Dr. Georg Dersch
Tel.: +43(0)5 0555 34120
Fax: +43 50 555 22019
e-mail: georg.dersch@ages.at
Doc.No.: D-19186534

ANALYSIS REPORT

This analysis report including the analysis results contained within applies only to the present test object/s and the extent of the performed analyses. The testing laboratory had no influence on sampling, storage and transport prior to handover to AGES, unless sampling was carried out by AGES and is documented below. The measurement uncertainty, which results from sampling, is not included in the expanded measurement uncertainty (if stated), unless explicitly stated otherwise. This analysis report may only be copied as a whole; it may only be forwarded or published with the prior consent of AGES and may not be amended. Terms and Conditions of AGES apply. (All dates shown in format dd.mm.yyyy)

Order number: 22030378

External identification:	
Order date:	08.03.2022
Invoice recipient:	Universität Wien Finanzwesen und Controlling, Universitätsring 1, 1010 Wien
Remark:	
Analysis report to be sent to:	Universität Wien, Verena Ibl

Comments:

Nach ÖNORM L 1091 ist die Bestimmung des Humusgehaltes zur Berechnung des Ergebnisses in kg Stickstoff pro Hektar erforderlich.

Die Bewertung der Nährstoffe erfolgt nach den Richtlinien für die

sachgerechte Düngung in der aktuell geltenden Auflage.

Informationen zur Interpretation der Bodenuntersuchungsergebnisse

und zur Erstellung des Düngeplans finden sie unter https://www.ages.at/boden

Institut für Nachhaltige Pflanzenproduktion
Spargelfeldstraße 191, 1220 Wien
Head of Institute: Mag. Bernhard Föger

AGES

Sample number: 22030378-001

External sample ID:	1
Lot/batch:	08/00967
Sample received on:	08.03.2022
Sample type:	Private sample
Beprobungstiefe von:	5
Beprobungstiefe bis:	20
Vorfrucht:	WROGGEN-MA
Ernterückstände eingearbeitet:	ja
Anzubauende Frucht:	HAFER
Nutzungsart:	ACKER
Erwarteter Ertrag:	4,60
Wirtschaftsdünger 1:	MIS Milchkühe Stallmist
Menge Wirtschaftsdünger1:	6
Gründigkeit:	mittelgründig
Bodenschwere:	mittel
Wasserverhältnisse:	trocken - mäßig feucht
Grobanteil:	gering - mäßig
Bewirtschaftungsform:	konventionell
PK Empfehlung Folgekulturen AK:	ja
Analysis carried out:	11.03.2022 - 27.04.2022

The analysis produced the following result:

Parameter	calc. as	Result	LOD	LOQ	Unit	N	R
Humus		4,3		0,3	%		1
Gehaltsklasse Humus	humos - C						1
Nitrogen (N)		0,3		0,2	mg/100g		2
Nitrate-nitrogen (NO3-N)		<LOQ		0,2	mg/100g		2
Ammonium-nitrogen (NH4-N)		0,1		0,1	mg/100g		2
Total nitrogen (N)	10 kg/ha						2
Boron (B)		0,2		0,2	mg/kg		3
Gehaltsklasse Bor	A - niedrig						3
Magnesium (Mg)		207		12	mg/kg		4
Gehaltsklasse Magnesium	E - sehr hoch						4
Iron (Fe)		239			mg/kg		5
Manganese (Mn)		81			mg/kg		5
Copper (Cu)		2,9			mg/kg		5
Zinc (Zn)		4,0			mg/kg		5
Gehaltsklasse Eisen	C - mittel						5
Gehaltsklasse Mangan	C - mittel						5
Gehaltsklasse Kupfer	C - mittel						5
Gehaltsklasse Zink	C - mittel						5
Stickstoff nachlieferbar		68		19	mg/kg/7d	x	6
Gehaltsklasse Stickstoff nachlieferbar	mittel					x	6
Total calcium carbonate (CaCO3)		<LOQ		0,5	%		7
Einstufung des Karbonatgehaltes	gering						7
Calcium (Ca)		7,38		2,60	cmolc/kg		8
Magnesium (Mg)		2,11		0,10	cmolc/kg		8
Potassium (K)		0,40		0,20	cmolc/kg		8
Sodium (Na)		0,05		0,04	cmolc/kg		8
Aluminium (Al)		<LOQ		0,06	cmolc/kg		8
Iron (Fe)		<LOQ		0,00	cmolc/kg		8

Institut für Nachhaltige Pflanzenproduktion
Spargelfeldstraße 191, 1220 Wien
Head of Institute: Mag. Bernhard Föger

Parameter	calc. as	Result	LOD	LOQ	Unit	N	R
Manganese (Mn)		0,03		0,01	cmolc/kg		8
H-Wert		0,002			cmolc/kg		8
Austauschkapazität		9,97			cmolc/kg		8
Variable a	74 % Ca vom Sorptionskomplex						8
Variable b	21,2 % Mg vom Sorptionskomplex						8
Variable c	4 % K vom Sorptionskomplex						8
Variable d	,5 % Na vom Sorptionskomplex						8
Variable e	0 % Al vom Sorptionskomplex						8
Variable f	0 % Fe vom Sorptionskomplex						8
Variable g	,3 % Mn vom Sorptionskomplex						8
Variable q	% Protonen vom Sorptionsk.						8
Total nitrogen (N)		0,203		0,070	%		9
Phosphorus (P)		72		20	mg/kg		10
Potassium (K)		161		18	mg/kg		10
Gehaltsklasse Phosphor	C - ausreichend						10
Gehaltsklasse Kalium	C - ausreichend						10
pH value		6,3					11
Bodenreaktion	schwach sauer						11
H-Wert	see comment						12

Abbreviations:

n.d. ... not detectable
n.e. ... not evaluable
LOD ... Limit of detection
LOQ ... Limit of quantification
NAD ... no abnormality detected
N ... Indication of non-accredited method
x ... method not accredited
R ... Comment
calc. as ... calculated as

Institut für Nachhaltige Pflanzenproduktion
Spargelfeldstraße 191, 1220 Wien
Head of Institute: Mag. Bernhard Föger

AGES

Sample number: 22030378-002

External sample ID: 2
Lot/batch: 08/00968
Sample received on: 08.03.2022
Sample type: Private sample

Beprobungstiefe von: 5
Beprobungstiefe bis: 20
Vorfrucht: HAFER
Ernterückstände eingearbeitet: ja
Anzubauende Frucht: KLEE
Nutzungsart: ACKER
Wirtschaftsdünger 1: MIS Milchkühe Stallmist
Menge Wirtschaftsdünger1: 9
Gründigkeit: mittelgründig
Bodenschwere: mittel
Wasserverhältnisse: trocken - mäßig feucht
Grobanteil: gering - mäßig
Bewirtschaftungsform: konventionell
PK Empfehlung Folgekulturen AK: ja

Analysis carried out: 11.03.2022 - 27.04.2022

The analysis produced the following result:

Parameter	calc. as	Result	LOD	LOQ	Unit	N	R
Humus		4,4		0,3	%		1
Gehaltsklasse Humus	humos - C						1
Nitrogen (N)		0,5		0,2	mg/100g		2
Nitrate-nitrogen (NO3-N)		0,3		0,2	mg/100g		2
Ammonium-nitrogen (NH4-N)		0,2		0,1	mg/100g		2
Total nitrogen (N)	15 kg/ha						2
Boron (B)		0,2		0,2	mg/kg		3
Gehaltsklasse Bor	A - niedrig						3
Magnesium (Mg)		99		12	mg/kg		4
Gehaltsklasse Magnesium	C - ausreichend						4
Iron (Fe)		479			mg/kg		5
Manganese (Mn)		182			mg/kg		5
Copper (Cu)		2,9			mg/kg		5
Zinc (Zn)		5,2			mg/kg		5
Gehaltsklasse Eisen	E - hoch						5
Gehaltsklasse Mangan	C - mittel						5
Gehaltsklasse Kupfer	C - mittel						5
Gehaltsklasse Zink	C - mittel						5
Stickstoff nachlieferbar		87		19	mg/kg/7d	**x**	6
Gehaltsklasse Stickstoff nachlieferbar	hoch					**x**	6
Total calcium carbonate (CaCO3)		<LOQ		0,5	%		7
Einstufung des Karbonatgehaltes	gering						7
Calcium (Ca)		7,37		2,60	cmolc/kg		8
Magnesium (Mg)		1,09		0,10	cmolc/kg		8
Potassium (K)		0,26		0,20	cmolc/kg		8
Sodium (Na)		0,22		0,04	cmolc/kg		8
Aluminium (Al)		0,09		0,06	cmolc/kg		8
Iron (Fe)		<LOQ		0,00	cmolc/kg		8
Manganese (Mn)		0,09		0,01	cmolc/kg		8

Institut für Nachhaltige Pflanzenproduktion
Spargelfeldstraße 191, 1220 Wien
Head of Institute: Mag. Bernhard Föger

Parameter	calc. as	Result	LOD	LOQ	Unit	N	R
H-Wert		0,002			cmolc/kg		8
Austauschkapazität		9,12			cmolc/kg		8
Variable a	80,8 % Ca vom Sorptionskomplex						8
Variable b	12 % Mg vom Sorptionskomplex						8
Variable c	2,8 % K vom Sorptionskomplex						8
Variable d	2,5 % Na vom Sorptionskomplex						8
Variable e	1 % Al vom Sorptionskomplex						8
Variable f	0 % Fe vom Sorptionskomplex						8
Variable g	1 % Mn vom Sorptionskomplex						8
Variable q	% Protonen vom Sorptionsk.						8
Total nitrogen (N)		0,211		0,070	%		9
Phosphorus (P)		53		20	mg/kg		10
Potassium (K)		96		18	mg/kg		10
Gehaltsklasse Phosphor	C - ausreichend						10
Gehaltsklasse Kalium	B - niedrig						10
pH value		5,5					11
Bodenreaktion	sauer						11
pH value		6,31					13
H-Wert	see comment						12

Abbreviations:

n.d. ... not detectable
n.e. ... not evaluable
LOD ... Limit of detection
LOQ ... Limit of quantification
NAD ... no abnormality detected
N ... Indication of non-accredited method
x ... method not accredited
R ... Comment
calc. as ... calculated as

Institut für Nachhaltige Pflanzenproduktion
Spargelfeldstraße 191, 1220 Wien
Head of Institute: Mag. Bernhard Föger

Sample number: 22030378-003

External sample ID:	3
Lot/batch:	06/00671
Sample received on:	08.03.2022
Sample type:	Private sample
Beprobungstiefe von:	0
Beprobungstiefe bis:	25
Ernterückstände eingearbeitet:	nein
Anzubauende Frucht:	WROGGEN-MA
Nutzungsart:	ACKER
Erwarteter Ertrag:	5,00
Gründigkeit:	mittelgründig
Bodenschwere:	mittel
Wasserverhältnisse:	trocken - mäßig feucht
Grobanteil:	kein
Bewirtschaftungsform:	konventionell
PK Empfehlung Folgekulturen AK:	ja
Analysis carried out:	11.03.2022 - 27.04.2022

The analysis produced the following result:

Parameter	calc. as	Result	LOD	LOQ	Unit	N	R
Boron (B)		2,5		0,2	mg/kg		3
Gehaltsklasse Bor	E - hoch						3
Magnesium (Mg)		158		12	mg/kg		4
Gehaltsklasse Magnesium	D - hoch						4
Iron (Fe)		54			mg/kg		5
Manganese (Mn)		93			mg/kg		5
Copper (Cu)		4,2			mg/kg		5
Zinc (Zn)		3,6			mg/kg		5
Gehaltsklasse Eisen	C - mittel						5
Gehaltsklasse Mangan	C - mittel						5
Gehaltsklasse Kupfer	C - mittel						5
Gehaltsklasse Zink	C - mittel						5
Stickstoff nachlieferbar		36		19	mg/kg/7d	**x**	6
Gehaltsklasse Stickstoff nachlieferbar	mittel					**x**	6
Total calcium carbonate ($CaCO_3$)		9,7		0,5	%		7
Einstufung des Karbonatgehaltes	hoch						7
Calcium (Ca)		18,16		2,60	cmolc/kg		8
Magnesium (Mg)		1,88		0,10	cmolc/kg		8
Potassium (K)		0,43		0,20	cmolc/kg		8
Sodium (Na)		<LOQ		0,04	cmolc/kg		8
Aluminium (Al)		<LOQ		0,06	cmolc/kg		8
Iron (Fe)		<LOQ		0,00	cmolc/kg		8
Manganese (Mn)		<LOQ		0,01	cmolc/kg		8
H-Wert		0,002			cmolc/kg		8
Austauschkapazität		20,48			cmolc/kg		8
Variable a	88,7 % Ca vom Sorptionskomplex						8
Variable b	9,2 % Mg vom Sorptionskomplex						8
Variable c	2,1 % K vom Sorptionskomplex						8
Variable d	,1 % Na vom Sorptionskomplex						8
Variable e	0 % Al vom Sorptionskomplex						8
Variable f	0 % Fe vom Sorptionskomplex						8

Parameter	calc. as	Result	LOD	LOQ	Unit	N	R
Variable g	0 % Mn vom Sorptionskomplex						8
Variable q	% Protonen vom Sorptionsk.						8
Total nitrogen (N)		0,160		0,070	%		9
Phosphorus (P)		78		20	mg/kg		10
Potassium (K)		128		18	mg/kg		10
Gehaltsklasse Phosphor	C - ausreichend						10
Gehaltsklasse Kalium	C - ausreichend						10
pH value		7,4					11
Bodenreaktion	alkalisch						11
Humus		3,1		0,3	%		1
Gehaltsklasse Humus	humos - C						1
H-Wert	see comment						12

Abbreviations:

n.d. ... not detectable
n.e. ... not evaluable
LOD ... Limit of detection
LOQ ... Limit of quantification
NAD ... no abnormality detected
N ... Indication of non-accredited method
x ... method not accredited
R ... Comment
calc. as ... calculated as

Comments (Test methods used):

1.) Bestimmung des organischen Kohlenstoffs in Böden durch trockene Verbrennung
External standard: ÖNORM L 1080: 2013 03 15

2.) Bestimmung des mineralischen Stickstoffs in Böden mittels SFAS
External standard: ÖNORM L1091

3.) Bestimmung von "pflanzenverfügbarem" Bor in Böden mittels ICP-OES
External standard: ÖNORM L 1090: 2010 12 01

4.) Bestimmung von CaCl2-extrahierbarem Magnesium in Böden mittels AAS-Flamme
External standard: ÖNORM L 1093: 2010 12 01

5.) Bestimmung von EDTA-extrahierbarem Fe, Mn, Cu und Zn in Böden mittels ICP-OES
External standard: ÖNORM L1098

6.) Bestimmung von nachlieferbarem Stickstoff in Böden im anaeroben Brutversuch mittels SFAS-Autoanalyzer

7.) Bestimmung von Karbonat in Böden und Kompost
External standard: ÖNORM L1084 Bezugsbasis:lufttrocken, ÖNORM EN ISO 10693 Bezugsbasis:lufttrocken, Kompostverordnung Anlage 5-3.3

8.) Bestimmung der austauschbaren Kationen und der effektiven Austauschkapazität mittels ICP-OES und AAS
External standard: ÖNORM L1086-1

9.) Bestimmung von Gesamtstickstoff in Böden durch trockene Verbrennung
External standard: ÖNORM L1095, ÖNORM EN 16168

10.) Bestimmung von P und K in Böden nach der CAL Methode mittels SFAS Autoanalyser
External standard: ÖNORM L 1087: 2012 12 01

11.) Bestimmung des pH-Werts in Böden
External standard: ÖNORM L 1083: 2006 04 01 und ÖNORM EN 15933: 2012 10 01 Standzeit vor der Messung max 24h

12.) Aufgrund eines pH-Wertes >5 wird der H-Wert nicht bestimmt und nicht verrechnet.

13.) Bestimmung des Kalkbedarfs von Mineralböden nach Schachtschabel
External standard: VDLUFA Methodenbuch Band 1 Untersuchung von Böden, Methode A 5.2.1, Doc. ID: 4887

Authorised signatory:

DI Dr. Georg Dersch

----- End of analysis report -----

Institut für Nachhaltige Pflanzenproduktion
Spargelfeldstraße 191, 1220 Wien
Head of Institute: Mag. Bernhard Föger

Signaturwert	kWYRcYYZduSdn/0SLq66s3GDGbiAS4GmR5r4+Uo/d0QEimgdULr7b3++ot7l1GE90FtvoDdxbjZqcS1xgWCIJhteW4/EG8BrL7juoaqpJptVoFT449dgQkFXqhqWFCgvI6uaFjI7/ITzYJjAzozGxG0RX02uhc4MjQxbvYCyfj49REqC794rOgkRS2Nwlq+zhAIkv6PxG58RhVDP/Y5LBifKybMXzeB05lga7clhgVrlhJK6biiuz4FL3PEce/zho2qZLLCMH3gwHX9OLoj0RNa/wZ9OGG84N2cJTnXlmVg5o4fpwzgnpSTTh7ZIZUORKhMPrjuH9vCHEZ0iuDv26Q==	
	Unterzeichner	serialNumber=586178147653 CN=Agentur für Gesundheit und Ernährungssicherheit GmbH C=AT
	Datum/Zeit-UTC	2023-03-29T06:43:13Z
	Aussteller-Zertifikat	CN=a-sign-corporate-07,OU=a-sign-corporate-07,O=A-Trust Ges. f. Sicherheitssysteme im elektr. Datenverkehr GmbH,C=AT
	Serien-Nr.	419848915
	Methode	urn:pdfsigfilter:bka.gv.at:binaer:v1.1.0
	Parameter	etsi-bka-moa-1.0
Prüfinformation	Dieses Dokument wurde amtssigniert.<br>Informationen zur Prüfung der elektronischen Signatur und des Ausdrucks finden Sie unter http://www.signaturpruefung.gv.at	
