# Supplementary material for: A bench-top Dark-Root device built with LEGO® bricks enables a non-invasive plant root development analysis in soil conditions mirroring nature
Source: Front Plant Sci. 2023 May 31;14:1166511. doi: 10.3389/fpls.2023.1166511 (PMC10264708; doi:10.3389/fpls.2023.1166511)
Supplement: Supplementary Data Sheet 5 — R-script used for analysis of proteomic data and graphic representation of results. [file DataSheet_5.pdf]

```
#---------------------------------------------------------
install.packages("readxl")
library(readxl)
install.packages("ggplot2")
library(ggplot2)
install.packages("reshape2")
library(reshape2)
install.packages("ggpubr")
library(ggpubr)
install.packages("tidyverse")
library(tidyverse)
install.packages("broom")
library(broom)
install.packages("AICcmodavg")
library(AICcmodavg)
installed.packages("mvtnorm")
installed.packages("survival")
installed.packages("TH.data")
installed.packages("MASS")

install.packages("multcomp")
library(multcomp)
install.packages("DescTools")
library(DescTools)
install.packages("multcompView")
library(multcompView)
install.packages("emmeans")
library(emmeans)

installed.packages("xlsx")
library(xlsx)
installed.packages("readxl")
library(readxl)


library(tidyverse)
library(dplyr)
install.packages("gplots")
library(gplots)
library(tidyverse)
install.packages("pheatmap")
library(pheatmap)
library(ggfortify)

install.packages("autoplot")
library(ggfortify)

#--------------------



#used logarithmized data (log10(x+1)) for the analysis...
```

```
library(readxl)

#---------------------------------------
#only with tap data...see C:...pca_r_try_29.12.2022.xlsx -
mappe:pca_log_woprot_onlytap

library(readxl)
pca_log_woprot_onlytap <- read_excel("pca_r_try_29.12.2022.xlsx",
                             sheet = "pca_log_woprot_onlytap",
col_names = FALSE,
                             col_types = c("text",
                                           "numeric", "numeric",
"numeric",
                                           "numeric", "numeric",
"numeric",
                                           "numeric", "numeric",
"numeric",
                                           "numeric", "numeric",
"numeric",
                                           "numeric", "numeric",
"numeric",
                                           "numeric", "numeric",
"numeric",
                                           "numeric", "numeric",
"numeric",
                                           "numeric", "numeric",
"numeric",
                                           "numeric", "numeric",
"numeric",
                                           "numeric", "numeric",
"numeric",
                                           "numeric", "numeric",
"numeric",
                                           "numeric", "numeric",
"numeric",
                                           "numeric", "numeric",
"numeric",
                                           "numeric", "numeric",
"numeric",
                                           "numeric", "numeric",
"numeric",
                                           "numeric", "numeric",
"numeric",
                                           "numeric", "numeric",
"numeric",
                                           "numeric", "numeric",
"numeric",
                                           "numeric", "numeric",
"numeric",
                                           "numeric", "numeric",
"numeric",
                                           "numeric", "numeric",
"numeric",
```

"numeric",
"numeric",
"numeric",
"numeric",
"numeric",
"numeric",
"numeric",
"numeric",
"numeric",
"numeric",
"numeric",
"numeric",
"numeric",
"numeric",
"numeric",
"numeric",
"numeric",
"numeric",
"numeric",
"numeric",
"numeric",
"numeric",
"numeric",
"numeric",
"numeric",
"numeric",
"numeric",
"numeric", "numeric",
"numeric", "numeric",
"numeric", "numeric",
"numeric", "numeric",
"numeric", "numeric",
"numeric", "numeric",
"numeric", "numeric",
"numeric", "numeric",
"numeric", "numeric",
"numeric", "numeric",
"numeric", "numeric",
"numeric", "numeric",
"numeric", "numeric",
"numeric", "numeric",
"numeric", "numeric",
"numeric", "numeric",
"numeric", "numeric",
"numeric", "numeric",
"numeric", "numeric",
"numeric", "numeric",
"numeric", "numeric",
"numeric", "numeric",
"numeric", "numeric",
"numeric", "numeric",
"numeric", "numeric",
"numeric", "numeric",
"numeric", "numeric",

"numeric",
"numeric",
"numeric",
"numeric",
"numeric",
"numeric",
"numeric",
"numeric",
"numeric",
"numeric",
"numeric",
"numeric",
"numeric",
"numeric",
"numeric",
"numeric",
"numeric",
"numeric",
"numeric",
"numeric",
"numeric",
"numeric",
"numeric",
"numeric",
"numeric",
"numeric",
"numeric",
"numeric", "numeric",
"numeric", "numeric",
"numeric", "numeric",
"numeric", "numeric",
"numeric", "numeric",
"numeric", "numeric",
"numeric", "numeric",
"numeric", "numeric",
"numeric", "numeric",
"numeric", "numeric",
"numeric", "numeric",
"numeric", "numeric",
"numeric", "numeric",
"numeric", "numeric",
"numeric", "numeric",
"numeric", "numeric",
"numeric", "numeric",
"numeric", "numeric",
"numeric", "numeric",
"numeric", "numeric",
"numeric", "numeric",
"numeric", "numeric",
"numeric", "numeric",
"numeric", "numeric",
"numeric", "numeric",
"numeric", "numeric",
"numeric", "numeric",

"numeric", "numeric",
"numeric",
"numeric", "numeric",
"numeric",
"numeric", "numeric",
"numeric",
"numeric", "numeric",
"numeric",
"numeric", "numeric",
"numeric",
"numeric", "numeric",
"numeric",
"numeric", "numeric",
"numeric",
"numeric", "numeric",
"numeric",
"numeric", "numeric",
"numeric",
"numeric", "numeric",
"numeric",
"numeric", "numeric",
"numeric",
"numeric", "numeric",
"numeric",
"numeric", "numeric",
"numeric",
"numeric", "numeric",
"numeric",
"numeric", "numeric",
"numeric",
"numeric", "numeric",
"numeric",
"numeric", "numeric",
"numeric",
"numeric", "numeric",
"numeric",
"numeric", "numeric",
"numeric",
"numeric", "numeric",
"numeric",
"numeric", "numeric",
"numeric",
"numeric", "numeric",
"numeric",
"numeric", "numeric",
"numeric",
"numeric", "numeric",
"numeric",
"numeric", "numeric",
"numeric",
"numeric", "numeric",
"numeric",
"numeric", "numeric",
"numeric",

"numeric",
"numeric",
"numeric",
"numeric",
"numeric",
"numeric",
"numeric",
"numeric",
"numeric",
"numeric",
"numeric",
"numeric",
"numeric",
"numeric",
"numeric",
"numeric",
"numeric",
"numeric",
"numeric",
"numeric",
"numeric",
"numeric",
"numeric",
"numeric",
"numeric",
"numeric",
"numeric",
"numeric", "numeric",
"numeric", "numeric",
"numeric", "numeric",
"numeric", "numeric",
"numeric", "numeric",
"numeric", "numeric",
"numeric", "numeric",
"numeric", "numeric",
"numeric", "numeric",
"numeric", "numeric",
"numeric", "numeric",
"numeric", "numeric",
"numeric", "numeric",
"numeric", "numeric",
"numeric", "numeric",
"numeric", "numeric",
"numeric", "numeric",
"numeric", "numeric",
"numeric", "numeric",
"numeric", "numeric",
"numeric", "numeric",
"numeric", "numeric",
"numeric", "numeric",
"numeric", "numeric",
"numeric", "numeric",
"numeric", "numeric",
"numeric", "numeric",

```
"numeric",
"numeric",
"numeric",
"numeric",
"numeric",
"numeric",
"numeric",
"numeric",
"numeric",
"numeric",
"numeric",
"numeric",
"numeric",
"numeric",
"numeric",
"numeric",
"numeric",
"numeric",
"numeric",
"numeric",
"numeric",
"numeric",
"numeric",
"numeric",
"numeric",
"numeric",
"numeric",
```

```
"numeric", "numeric",
"numeric", "numeric",
"numeric", "numeric",
"numeric", "numeric",
"numeric", "numeric",
"numeric", "numeric",
"numeric", "numeric",
"numeric", "numeric",
"numeric", "numeric",
"numeric", "numeric",
"numeric", "numeric",
"numeric", "numeric",
"numeric", "numeric",
"numeric", "numeric",
"numeric", "numeric",
"numeric", "numeric",
"numeric", "numeric",
"numeric", "numeric",
"numeric", "numeric",
"numeric", "numeric",
"numeric", "numeric",
"numeric", "numeric",
"numeric", "numeric",
"numeric", "numeric",
"numeric", "numeric",
"numeric", "numeric",
"numeric", "numeric",
```

"numeric",
"numeric",
"numeric",
"numeric",
"numeric",
"numeric",
"numeric",
"numeric",
"numeric",
"numeric",
"numeric",
"numeric",
"numeric",
"numeric",
"numeric",
"numeric",
"numeric",
"numeric",
"numeric",
"numeric",
"numeric",
"numeric",
"numeric",
"numeric",
"numeric",
"numeric",
"numeric",
"numeric", "numeric",
"numeric", "numeric",
"numeric", "numeric",
"numeric", "numeric",
"numeric", "numeric",
"numeric", "numeric",
"numeric", "numeric",
"numeric", "numeric",
"numeric", "numeric",
"numeric", "numeric",
"numeric", "numeric",
"numeric", "numeric",
"numeric", "numeric",
"numeric", "numeric",
"numeric", "numeric",
"numeric", "numeric",
"numeric", "numeric",
"numeric", "numeric",
"numeric", "numeric",
"numeric", "numeric",
"numeric", "numeric",
"numeric", "numeric",
"numeric", "numeric",
"numeric", "numeric",
"numeric", "numeric",
"numeric", "numeric",
"numeric", "numeric",

"numeric",
"numeric",
"numeric",
"numeric",
"numeric",
"numeric",
"numeric",
"numeric",
"numeric",
"numeric",
"numeric",
"numeric",
"numeric",
"numeric",
"numeric",
"numeric",
"numeric",
"numeric",
"numeric",
"numeric",
"numeric",
"numeric",
"numeric",
"numeric",
"numeric",
"numeric",
"numeric",
"numeric", "numeric",
"numeric", "numeric",
"numeric", "numeric",
"numeric", "numeric",
"numeric", "numeric",
"numeric", "numeric",
"numeric", "numeric",
"numeric", "numeric",
"numeric", "numeric",
"numeric", "numeric",
"numeric", "numeric",
"numeric", "numeric",
"numeric", "numeric",
"numeric", "numeric",
"numeric", "numeric",
"numeric", "numeric",
"numeric", "numeric",
"numeric", "numeric",
"numeric", "numeric",
"numeric", "numeric",
"numeric", "numeric",
"numeric", "numeric",
"numeric", "numeric",
"numeric", "numeric",
"numeric", "numeric",
"numeric", "numeric",
"numeric", "numeric",

"numeric",
"numeric",
"numeric",
"numeric",
"numeric",
"numeric",
"numeric",
"numeric",
"numeric",
"numeric",
"numeric",
"numeric",
"numeric",
"numeric",
"numeric",
"numeric",
"numeric",
"numeric",
"numeric",
"numeric",
"numeric",
"numeric",
"numeric",
"numeric",
"numeric",
"numeric",
"numeric",
"numeric", "numeric",
"numeric", "numeric",
"numeric", "numeric",
"numeric", "numeric",
"numeric", "numeric",
"numeric", "numeric",
"numeric", "numeric",
"numeric", "numeric",
"numeric", "numeric",
"numeric", "numeric",
"numeric", "numeric",
"numeric", "numeric",
"numeric", "numeric",
"numeric", "numeric",
"numeric", "numeric",
"numeric", "numeric",
"numeric", "numeric",
"numeric", "numeric",
"numeric", "numeric",
"numeric", "numeric",
"numeric", "numeric",
"numeric", "numeric",
"numeric", "numeric",
"numeric", "numeric",
"numeric", "numeric",
"numeric", "numeric",
"numeric", "numeric",

```
"numeric",
"numeric",
"numeric",
"numeric",
"numeric",
"numeric",
"numeric",
"numeric",
"numeric",
"numeric",
"numeric",
"numeric",
"numeric",
"numeric",
"numeric",
"numeric",
"numeric",
"numeric",
"numeric",
"numeric",
"numeric",
"numeric",
"numeric",
"numeric",
"numeric",
"numeric",
"numeric",
"numeric", "numeric",
"numeric", "numeric",
"numeric", "numeric",
"numeric", "numeric",
"numeric", "numeric",
"numeric", "numeric",
"numeric", "numeric",
"numeric", "numeric",
"numeric", "numeric",
"numeric", "numeric",
"numeric", "numeric",
"numeric", "numeric",
"numeric", "numeric",
"numeric", "numeric",
"numeric", "numeric",
"numeric", "numeric",
"numeric", "numeric",
"numeric", "numeric",
"numeric", "numeric",
"numeric", "numeric",
"numeric", "numeric",
"numeric", "numeric",
"numeric", "numeric",
"numeric", "numeric",
"numeric", "numeric",
"numeric", "numeric",
"numeric", "numeric",
```

"numeric",
"numeric",
"numeric",
"numeric",
"numeric",
"numeric",
"numeric",
"numeric",
"numeric",
"numeric",
"numeric",
"numeric",
"numeric",
"numeric",
"numeric",
"numeric",
"numeric",
"numeric",
"numeric",
"numeric",
"numeric",
"numeric",
"numeric",
"numeric",
"numeric",
"numeric",
"numeric",
"numeric", "numeric",
"numeric", "numeric",
"numeric", "numeric",
"numeric", "numeric",
"numeric", "numeric",
"numeric", "numeric",
"numeric", "numeric",
"numeric", "numeric",
"numeric", "numeric",
"numeric", "numeric",
"numeric", "numeric",
"numeric", "numeric",
"numeric", "numeric",
"numeric", "numeric",
"numeric", "numeric",
"numeric", "numeric",
"numeric", "numeric",
"numeric", "numeric",
"numeric", "numeric",
"numeric", "numeric",
"numeric", "numeric",
"numeric", "numeric",
"numeric", "numeric",
"numeric", "numeric",
"numeric", "numeric",
"numeric", "numeric",
"numeric", "numeric",

"numeric",
"numeric",
"numeric",
"numeric",
"numeric",
"numeric",
"numeric",
"numeric",
"numeric",
"numeric",
"numeric",
"numeric",
"numeric",
"numeric",
"numeric",
"numeric",
"numeric",
"numeric",
"numeric",
"numeric",
"numeric",
"numeric",
"numeric",
"numeric",
"numeric",
"numeric",
"numeric",
"numeric", "numeric",
"numeric", "numeric",
"numeric", "numeric",
"numeric", "numeric",
"numeric", "numeric",
"numeric", "numeric",
"numeric", "numeric",
"numeric", "numeric",
"numeric", "numeric",
"numeric", "numeric",
"numeric", "numeric",
"numeric", "numeric",
"numeric", "numeric",
"numeric", "numeric",
"numeric", "numeric",
"numeric", "numeric",
"numeric", "numeric",
"numeric", "numeric",
"numeric", "numeric",
"numeric", "numeric",
"numeric", "numeric",
"numeric", "numeric",
"numeric", "numeric",
"numeric", "numeric",
"numeric", "numeric",
"numeric", "numeric",
"numeric", "numeric",

"numeric",
"numeric",
"numeric",
"numeric",
"numeric",
"numeric",
"numeric",
"numeric",
"numeric",
"numeric",
"numeric",
"numeric",
"numeric",
"numeric",
"numeric",
"numeric",
"numeric",
"numeric",
"numeric",
"numeric",
"numeric",
"numeric",
"numeric",
"numeric",
"numeric",
"numeric",
"numeric",

"numeric", "numeric",
"numeric", "numeric",
"numeric", "numeric",
"numeric", "numeric",
"numeric", "numeric",
"numeric", "numeric",
"numeric", "numeric",
"numeric", "numeric",
"numeric", "numeric",
"numeric", "numeric",
"numeric", "numeric",
"numeric", "numeric",
"numeric", "numeric",
"numeric", "numeric",
"numeric", "numeric",
"numeric", "numeric",
"numeric", "numeric",
"numeric", "numeric",
"numeric", "numeric",
"numeric", "numeric",
"numeric", "numeric",
"numeric", "numeric",
"numeric", "numeric",
"numeric", "numeric",
"numeric", "numeric",
"numeric", "numeric",
"numeric", "numeric",

"numeric",
"numeric",
"numeric",
"numeric",
"numeric",
"numeric",
"numeric",
"numeric",
"numeric",
"numeric",
"numeric",
"numeric",
"numeric",
"numeric",
"numeric",
"numeric",
"numeric",
"numeric",
"numeric",
"numeric",
"numeric",
"numeric",
"numeric",
"numeric",
"numeric",
"numeric",
"numeric",
"numeric", "numeric",
"numeric", "numeric",
"numeric", "numeric",
"numeric", "numeric",
"numeric", "numeric",
"numeric", "numeric",
"numeric", "numeric",
"numeric", "numeric",
"numeric", "numeric",
"numeric", "numeric",
"numeric", "numeric",
"numeric", "numeric",
"numeric", "numeric",
"numeric", "numeric",
"numeric", "numeric",
"numeric", "numeric",
"numeric", "numeric",
"numeric", "numeric",
"numeric", "numeric",
"numeric", "numeric",
"numeric", "numeric",
"numeric", "numeric",
"numeric", "numeric",
"numeric", "numeric",
"numeric", "numeric",
"numeric", "numeric",
"numeric", "numeric",

"numeric",
"numeric",
"numeric",
"numeric",
"numeric",
"numeric",
"numeric",
"numeric",
"numeric",
"numeric",
"numeric",
"numeric",
"numeric",
"numeric",
"numeric",
"numeric",
"numeric",
"numeric",
"numeric",
"numeric",
"numeric",
"numeric",
"numeric",
"numeric",
"numeric",
"numeric",
"numeric",
"numeric", "numeric",
"numeric", "numeric",
"numeric", "numeric",
"numeric", "numeric",
"numeric", "numeric",
"numeric", "numeric",
"numeric", "numeric",
"numeric", "numeric",
"numeric", "numeric",
"numeric", "numeric",
"numeric", "numeric",
"numeric", "numeric",
"numeric", "numeric",
"numeric", "numeric",
"numeric", "numeric",
"numeric", "numeric",
"numeric", "numeric",
"numeric", "numeric",
"numeric", "numeric",
"numeric", "numeric",
"numeric", "numeric",
"numeric", "numeric",
"numeric", "numeric",
"numeric", "numeric",
"numeric", "numeric",
"numeric", "numeric",
"numeric", "numeric",

"numeric",
"numeric",
"numeric",
"numeric",
"numeric",
"numeric",
"numeric",
"numeric",
"numeric",
"numeric",
"numeric",
"numeric",
"numeric",
"numeric",
"numeric",
"numeric",
"numeric",
"numeric",
"numeric",
"numeric",
"numeric",
"numeric",
"numeric",
"numeric",
"numeric",
"numeric",
"numeric",
"numeric", "numeric",
"numeric", "numeric",
"numeric", "numeric",
"numeric", "numeric",
"numeric", "numeric",
"numeric", "numeric",
"numeric", "numeric",
"numeric", "numeric",
"numeric", "numeric",
"numeric", "numeric",
"numeric", "numeric",
"numeric", "numeric",
"numeric", "numeric",
"numeric", "numeric",
"numeric", "numeric",
"numeric", "numeric",
"numeric", "numeric",
"numeric", "numeric",
"numeric", "numeric",
"numeric", "numeric",
"numeric", "numeric",
"numeric", "numeric",
"numeric", "numeric",
"numeric", "numeric",
"numeric", "numeric",
"numeric", "numeric",
"numeric", "numeric",

"numeric",
"numeric",
"numeric",
"numeric",
"numeric",
"numeric",
"numeric",
"numeric",
"numeric",
"numeric",
"numeric",
"numeric",
"numeric",
"numeric",
"numeric",
"numeric",
"numeric",
"numeric",
"numeric",
"numeric",
"numeric",
"numeric",
"numeric",
"numeric",
"numeric",
"numeric",
"numeric",
"numeric", "numeric",
"numeric", "numeric",
"numeric", "numeric",
"numeric", "numeric",
"numeric", "numeric",
"numeric", "numeric",
"numeric", "numeric",
"numeric", "numeric",
"numeric", "numeric",
"numeric", "numeric",
"numeric", "numeric",
"numeric", "numeric",
"numeric", "numeric",
"numeric", "numeric",
"numeric", "numeric",
"numeric", "numeric",
"numeric", "numeric",
"numeric", "numeric",
"numeric", "numeric",
"numeric", "numeric",
"numeric", "numeric",
"numeric", "numeric",
"numeric", "numeric",
"numeric", "numeric",
"numeric", "numeric",
"numeric", "numeric",
"numeric", "numeric",

"numeric",
"numeric",
"numeric",
"numeric",
"numeric",
"numeric",
"numeric",
"numeric",
"numeric",
"numeric",
"numeric",
"numeric",
"numeric",
"numeric",
"numeric",
"numeric",
"numeric",
"numeric",
"numeric",
"numeric",
"numeric",
"numeric",
"numeric",
"numeric",
"numeric",
"numeric",
"numeric",
"numeric", "numeric",
"numeric", "numeric",
"numeric", "numeric",
"numeric", "numeric",
"numeric", "numeric",
"numeric", "numeric",
"numeric", "numeric",
"numeric", "numeric",
"numeric", "numeric",
"numeric", "numeric",
"numeric", "numeric",
"numeric", "numeric",
"numeric", "numeric",
"numeric", "numeric",
"numeric", "numeric",
"numeric", "numeric",
"numeric", "numeric",
"numeric", "numeric",
"numeric", "numeric",
"numeric", "numeric",
"numeric", "numeric",
"numeric", "numeric",
"numeric", "numeric",
"numeric", "numeric",
"numeric", "numeric",
"numeric", "numeric",
"numeric", "numeric",

"numeric",
"numeric",
"numeric",
"numeric",
"numeric",
"numeric",
"numeric",
"numeric",
"numeric",
"numeric",
"numeric",
"numeric",
"numeric",
"numeric",
"numeric",
"numeric",
"numeric",
"numeric",
"numeric",
"numeric",
"numeric",
"numeric",
"numeric",
"numeric",
"numeric",
"numeric",
"numeric",
"numeric", "numeric",
"numeric", "numeric",
"numeric", "numeric",
"numeric", "numeric",
"numeric", "numeric",
"numeric", "numeric",
"numeric", "numeric",
"numeric", "numeric",
"numeric", "numeric",
"numeric", "numeric",
"numeric", "numeric",
"numeric", "numeric",
"numeric", "numeric",
"numeric", "numeric",
"numeric", "numeric",
"numeric", "numeric",
"numeric", "numeric",
"numeric", "numeric",
"numeric", "numeric",
"numeric", "numeric",
"numeric", "numeric",
"numeric", "numeric",
"numeric", "numeric",
"numeric", "numeric",
"numeric", "numeric",
"numeric", "numeric",
"numeric", "numeric",

"numeric",
"numeric",
"numeric",
"numeric",
"numeric",
"numeric",
"numeric",
"numeric",
"numeric",
"numeric",
"numeric",
"numeric",
"numeric",
"numeric",
"numeric",
"numeric",
"numeric",
"numeric",
"numeric",
"numeric",
"numeric",
"numeric",
"numeric",
"numeric",
"numeric",
"numeric",
"numeric",
"numeric", "numeric",
"numeric", "numeric",
"numeric", "numeric",
"numeric", "numeric",
"numeric", "numeric",
"numeric", "numeric",
"numeric", "numeric",
"numeric", "numeric",
"numeric", "numeric",
"numeric", "numeric",
"numeric", "numeric",
"numeric", "numeric",
"numeric", "numeric",
"numeric", "numeric",
"numeric", "numeric",
"numeric", "numeric",
"numeric", "numeric",
"numeric", "numeric",
"numeric", "numeric",
"numeric", "numeric",
"numeric", "numeric",
"numeric", "numeric",
"numeric", "numeric",
"numeric", "numeric",
"numeric", "numeric",
"numeric", "numeric",
"numeric", "numeric",

"numeric",
"numeric",
"numeric",
"numeric",
"numeric",
"numeric",
"numeric",
"numeric",
"numeric",
"numeric",
"numeric",
"numeric",
"numeric",
"numeric",
"numeric",
"numeric",
"numeric",
"numeric",
"numeric",
"numeric",
"numeric",
"numeric",
"numeric",
"numeric",
"numeric",
"numeric",
"numeric",
"numeric", "numeric",
"numeric", "numeric",
"numeric", "numeric",
"numeric", "numeric",
"numeric", "numeric",
"numeric", "numeric",
"numeric", "numeric",
"numeric", "numeric",
"numeric", "numeric",
"numeric", "numeric",
"numeric", "numeric",
"numeric", "numeric",
"numeric", "numeric",
"numeric", "numeric",
"numeric", "numeric",
"numeric", "numeric",
"numeric", "numeric",
"numeric", "numeric",
"numeric", "numeric",
"numeric", "numeric",
"numeric", "numeric",
"numeric", "numeric",
"numeric", "numeric",
"numeric", "numeric",
"numeric", "numeric",
"numeric", "numeric",
"numeric", "numeric",

"numeric",
"numeric",
"numeric",
"numeric",
"numeric",
"numeric",
"numeric",
"numeric",
"numeric",
"numeric",
"numeric",
"numeric",
"numeric",
"numeric",
"numeric",
"numeric",
"numeric",
"numeric",
"numeric",
"numeric",
"numeric",
"numeric",
"numeric",
"numeric",
"numeric",
"numeric",
"numeric",
"numeric", "numeric",
"numeric", "numeric",
"numeric", "numeric",
"numeric", "numeric",
"numeric", "numeric",
"numeric", "numeric",
"numeric", "numeric",
"numeric", "numeric",
"numeric", "numeric",
"numeric", "numeric",
"numeric", "numeric",
"numeric", "numeric",
"numeric", "numeric",
"numeric", "numeric",
"numeric", "numeric",
"numeric", "numeric",
"numeric", "numeric",
"numeric", "numeric",
"numeric", "numeric",
"numeric", "numeric",
"numeric", "numeric",
"numeric", "numeric",
"numeric", "numeric",
"numeric", "numeric",
"numeric", "numeric",
"numeric", "numeric",
"numeric", "numeric",

"numeric",
"numeric",
"numeric",
"numeric",
"numeric",
"numeric",
"numeric",
"numeric",
"numeric",
"numeric",
"numeric",
"numeric",
"numeric",
"numeric",
"numeric",
"numeric",
"numeric",
"numeric",
"numeric",
"numeric",
"numeric",
"numeric",
"numeric",
"numeric",
"numeric",
"numeric",
"numeric",

"numeric", "numeric",
"numeric", "numeric",
"numeric", "numeric",
"numeric", "numeric",
"numeric", "numeric",
"numeric", "numeric",
"numeric", "numeric",
"numeric", "numeric",
"numeric", "numeric",
"numeric", "numeric",
"numeric", "numeric",
"numeric", "numeric",
"numeric", "numeric",
"numeric", "numeric",
"numeric", "numeric",
"numeric", "numeric",
"numeric", "numeric",
"numeric", "numeric",
"numeric", "numeric",
"numeric", "numeric",
"numeric", "numeric",
"numeric", "numeric",
"numeric", "numeric",
"numeric", "numeric",
"numeric", "numeric",
"numeric", "numeric",
"numeric", "numeric",

"numeric",
"numeric",
"numeric",
"numeric",
"numeric",
"numeric",
"numeric",
"numeric",
"numeric",
"numeric",
"numeric",
"numeric",
"numeric",
"numeric",
"numeric",
"numeric",
"numeric",
"numeric",
"numeric",
"numeric",
"numeric",
"numeric",
"numeric",
"numeric",
"numeric",
"numeric",
"numeric",
"numeric", "numeric",
"numeric", "numeric",
"numeric", "numeric",
"numeric", "numeric",
"numeric", "numeric",
"numeric", "numeric",
"numeric", "numeric",
"numeric", "numeric",
"numeric", "numeric",
"numeric", "numeric",
"numeric", "numeric",
"numeric", "numeric",
"numeric", "numeric",
"numeric", "numeric",
"numeric", "numeric",
"numeric", "numeric",
"numeric", "numeric",
"numeric", "numeric",
"numeric", "numeric",
"numeric", "numeric",
"numeric", "numeric",
"numeric", "numeric",
"numeric", "numeric",
"numeric", "numeric",
"numeric", "numeric",
"numeric", "numeric",
"numeric", "numeric",

"numeric",
"numeric",
"numeric",
"numeric",
"numeric",
"numeric",
"numeric",
"numeric",
"numeric",
"numeric",
"numeric",
"numeric",
"numeric",
"numeric",
"numeric",
"numeric",
"numeric",
"numeric",
"numeric",
"numeric",
"numeric",
"numeric",
"numeric",
"numeric",
"numeric",
"numeric",
"numeric",
"numeric", "numeric",
"numeric", "numeric",
"numeric", "numeric",
"numeric", "numeric",
"numeric", "numeric",
"numeric", "numeric",
"numeric", "numeric",
"numeric", "numeric",
"numeric", "numeric",
"numeric", "numeric",
"numeric", "numeric",
"numeric", "numeric",
"numeric", "numeric",
"numeric", "numeric",
"numeric", "numeric",
"numeric", "numeric",
"numeric", "numeric",
"numeric", "numeric",
"numeric", "numeric",
"numeric", "numeric",
"numeric", "numeric",
"numeric", "numeric",
"numeric", "numeric",
"numeric", "numeric",
"numeric", "numeric",
"numeric", "numeric",
"numeric", "numeric",

"numeric",
"numeric",
"numeric",
"numeric",
"numeric",
"numeric",
"numeric",
"numeric",
"numeric",
"numeric",
"numeric",
"numeric",
"numeric",
"numeric",
"numeric",
"numeric",
"numeric",
"numeric",
"numeric",
"numeric",
"numeric",
"numeric",
"numeric",
"numeric",
"numeric",
"numeric",
"numeric",
"numeric", "numeric",
"numeric", "numeric",
"numeric", "numeric",
"numeric", "numeric",
"numeric", "numeric",
"numeric", "numeric",
"numeric", "numeric",
"numeric", "numeric",
"numeric", "numeric",
"numeric", "numeric",
"numeric", "numeric",
"numeric", "numeric",
"numeric", "numeric",
"numeric", "numeric",
"numeric", "numeric",
"numeric", "numeric",
"numeric", "numeric",
"numeric", "numeric",
"numeric", "numeric",
"numeric", "numeric",
"numeric", "numeric",
"numeric", "numeric",
"numeric", "numeric",
"numeric", "numeric",
"numeric", "numeric",
"numeric", "numeric",
"numeric", "numeric",

```
                                                          "numeric", "numeric",
"numeric",
                                                          "numeric", "numeric",
"numeric",
                                                          "numeric", "numeric",
"numeric",
                                                          "numeric", "numeric",
"numeric",
                                                          "numeric", "numeric",
"numeric",
                                                          "numeric", "numeric",
"numeric",
                                                          "numeric", "numeric",
"numeric",
                                                          "numeric", "numeric",
"numeric",
                                                          "numeric", "numeric",
"numeric",
                                                          "numeric", "numeric",
"numeric",
                                                          "numeric", "numeric",
"numeric",
                                                          "numeric", "numeric",
"numeric",
                                                          "numeric", "numeric",
"numeric",
                                                          "numeric", "numeric",
"numeric",
                                                          "numeric", "numeric",
"numeric",
                                                          "numeric", "numeric",
"numeric",
                                                          "numeric", "numeric",
"numeric",
                                                          "numeric", "numeric",
"numeric",
                                                          "numeric", "numeric",
"numeric",
                                                          "numeric", "numeric",
"numeric",
                                                          "numeric", "numeric",
"numeric",
                                                          "numeric", "numeric",
"numeric",
                                                          "numeric", "numeric",
"numeric",
                                                          "numeric", "numeric",
"numeric",
                                                          "numeric"))

View(pca_log_woprot_onlytap)
pca_log_woprot_onlytap
pca_log_woprot_onlytap_matrix<- as.matrix(pca_log_woprot_onlytap)
#pca <- prcomp((pca_log_woprot), scale=TRUE)
pca_log_woprot_onlytap_matrix
```

```
pca_log_woprot_onlytap_new <- pca_log_woprot_onlytap[ ,
unlist(lapply(pca_log_woprot_onlytap,            # Remove non-
numeric columns
                                                     is.numeric))]
pca_log_woprot_onlytap_new
pca02012023_onlytap <- prcomp(pca_log_woprot_onlytap_new)
#worked!!!!! (https://statisticsglobe.com/r-error-colmeans-x-must-
be-numeric)
plot(pca02012023_onlytap$x[,1], pca02012023_onlytap$x[,2])
#worked!!!!   https://www.youtube.com/watch?v=0Jp4gsfOLMs&t=153s
pca02012023_onlytap.var <-pca02012023_onlytap$sdev^2
pca02012023_onlytap.var.per <- round(pca02012023_onlytap.var/
sum(pca02012023_onlytap.var)*100,1)
barplot(pca02012023_onlytap.var.per, main="Scree Plot",
xlab="Principal Component", ylab="Percent Variation") # i have PCs
for every sample....!

pca02012023_onlytap.var.per

#pca plot only tap



group_onlytap <- c("16DAI_tap_DR", "16DAI_tap_DR",  "16DAI_tap_DR",
            "16DAI_tap_DR",  "16DAI_tap_DS",  "16DAI_tap_DS",
"16DAI_tap_DS",  "16DAI_tap_LR",  "16DAI_tap_LR",
            "16DAI_tap_LR",  "16DAI_tap_LS",  "16DAI_tap_LS",
"16DAI_tap_LS",  "16DAI_tap_LS",  "8DAI_tap_DR" ,
            "8DAI_tap_DR",   "8DAI_tap_DR",  "8DAI_tap_DR",
"8DAI_tap_DS",
            "8DAI_tap_DS",   "8DAI_tap_DS" ,  "8DAI_tap_DS",
"8DAI_tap_LR",   "8DAI_tap_LR",   "8DAI_tap_LR",
            "8DAI_tap_LR",   "8DAI_tap_LS",   "8DAI_tap_LS",
"8DAI_tap_LS",   "8DAI_tap_LS")
group_onlytap


plot(pca02012023_onlytap$x[,1],pca02012023_onlytap$x[,2],
     col=factor(group_onlytap),main="PCA plot tap - light - dark",
     pch=19, xlab="PC1 (53.5%)",
     ylab="PC2 (13.7%)")

library(ggfortify)
autoplot(pca02012023_onlytap, data = pca_log_woprot_onlytap_new,
main="PCA plot tap - light - dark", shape = FALSE, label.size = 3)#
hier andere dastellweise mit Zahlen als beschriftung... 1-53

#-------------------------------------------------

#----------------------------------------------------------------

# try to understand which proteins have the most impact on the
```

```
datashift.To get a sence of how meaningfull those clusters are...
#how to use loding scores to determine which proteins have the
larges effect on where samples are plotted in the PCA plot...:

#--------------------------------------------------------------------
-------
#top10 and top 100 proteins in x and y of tap only data...

plot(pca02012023_onlytap$x[,1],pca02012023_onlytap$x[,2],
     col=factor(group_onlytap),main="PCA plot tap - light - dark",
     pch=19, xlab="PC1 (53.5%)",
     ylab="PC2 (13.7%)")

# here only for pc1... x axis!!! (push to root on the left
(negative) and to shoot on the right (positive...!))

#top10x
loading_scores_only.tap.data <- pca02012023_onlytap$rotation[,1]
protein_scores_only.tap.data <- abs(loading_scores_only.tap.data)
#proteins that push samples to the left side of the graph will have
a large negative values and proteins that push samples to
# the right will have a large positive values.
# since we are interested in both sets, of proteins, we will use the
abs() function to sort based on the
# numbers magnitude rather from high to low
protein_score_ranked_only.tap.data <-
sort(protein_scores_only.tap.data, decreasing=TRUE) #here we sort
the magnitudes of the loading scores from high to low.
top_10_proteins_only.tap.data <-
names(protein_score_ranked_only.tap.data[1:10]) #now we get the
names of the top 10 proteins with the largest loading score
magnitudes.

#top_10_proteins_only.tap.data
#[1] "...2021" "...1714" "...1505" "...1929" "...1587" "...1629"
"...1824" "...319"  "...1550" "...1233"
#pca02012023_onlytap$rotation[top_10_proteins_only.tap.data,1]
#  ...2021    ...1714    ...1505    ...1929    ...1587    ...1629
...1824     ...319    ...1550    ...1233
#0.05987511 0.05813526 0.05634171 0.05563724 0.05542639 0.05534252
0.05508708 0.05505511 0.05456930 0.05440111

#top100x

top_100_proteins_only.tap.data <-
names(protein_score_ranked_only.tap.data[1:100]) #now we get the
names of the top 100 proteins with the largest loading score
magnitudes.
top_100_proteins_only.tap.data


#top_100_proteins_only.tap.data
#[1] "...2021" "...1714" "...1505" "...1929" "...1587" "...1629"
"...1824" "...319"  "...1550" "...1233" "...238"  "...1763"
```

```
"...1644"
#[14] "...513"  "...718"  "...506"  "...82"   "...913"  "...1611"
"...919"  "...295"  "...1341" "...1100" "...829"  "...1969"
"...1621"
#[27] "...1183" "...2141" "...374"  "...805"  "...840"  "...1122"
"...923"  "...1859" "...1172" "...1835" "...62"   "...366"  "...61"
#[40] "...949"  "...1567" "...667"  "...5"    "...60"   "...2158"
"...784"  "...630"  "...502"  "...1332" "...105"  "...1489"
"...1575"
#[53] "...1461" "...200"  "...1574" "...2071" "...135"  "...1355"
"...2153" "...434"  "...208"  "...1133" "...697"  "...931"
"...2140"
#[66] "...904"  "...149"  "...57"   "...2138" "...31"   "...277"
"...1003" "...902"  "...1033" "...2156" "...739"  "...853"
"...2155"
#[79] "...47"   "...1320" "...487"  "...48"   "...1690" "...845"
"...1786" "...663"  "...910"  "...1563" "...1512" "...1823"
"...1909"
#[92] "...484"  "...1214" "...1525" "...1846" "...531"  "...217"
"...1262" "...681"  "...1727"

pca02012023_onlytap$rotation[top_100_proteins_only.tap.data,1]

#pca02012023_onlytap$rotation[top_100_proteins_only.tap.data,1]
#...2021     ...1714     ...1505     ...1929     ...1587     ...1629
...1824      ...319     ...1550     ...1233      ...238
#0.05987511  0.05813526  0.05634171  0.05563724  0.05542639
0.05534252  0.05508708  0.05505511  0.05456930  0.05440111
0.05434530
#...1763     ...1644      ...513      ...718      ...506       ...82
...913     ...1611      ...919      ...295     ...1341
#0.05399269  0.05397580  0.05374762 -0.05372119  0.05353172
0.05345179  0.05325161  0.05324244  0.05316486  0.05297860
0.05292109
#...1100      ...829     ...1969     ...1621     ...1183     ...2141
...374      ...805      ...840     ...1122      ...923
#0.05284457  0.05278340  0.05277817  0.05269410  0.05245988
-0.05242046  0.05232486  0.05229337  0.05199437  0.05197257
0.05173940
#...1859     ...1172     ...1835       ...62      ...366       ...61
...949     ...1567      ...667        ...5       ...60
#0.05165274  0.05150787  0.05150376  0.05147276  0.05144228
0.05135306  0.05133218  0.05128910  0.05125957  0.05124869
-0.05108730
#...2158      ...784      ...630      ...502     ...1332      ...105
...1489     ...1575     ...1461      ...200     ...1574
#0.05107253  0.05098742  0.05094791 -0.05094680 -0.05089891
0.05089125  0.05086068  0.05069797  0.05061893 -0.05057830
0.05057046
#...2071      ...135     ...1355     ...2153      ...434      ...208
...1133      ...697      ...931     ...2140      ...904
#0.05050206  0.05047839  0.05045617  0.05043808  0.05043164
0.05041150  0.05039652  0.05034349 -0.05028054  0.05025242
0.05022082
```

```
#...149       ...57     ...2138       ...31      ...277     ...1003
...902     ...1033     ...2156      ...739      ...853
#0.05021939 -0.05014530  0.05009818  0.05007734  0.05001494
0.05001054 -0.05000042  0.04996464  0.04995126  0.04987414
0.04978606
#...2155       ...47     ...1320      ...487       ...48     ...1690
...845     ...1786      ...663      ...910     ...1563
#0.04975325  0.04974624  0.04967379  0.04964311 -0.04954971
0.04950957  0.04947598  0.04946429  0.04946056 -0.04941722
0.04941380
#...1512     ...1823     ...1909      ...484     ...1214     ...1525
...1846      ...531      ...217     ...1262      ...681
#0.04941251  0.04941233 -0.04924749  0.04918905  0.04893154
-0.04891581  0.04883143 -0.04878635  0.04874070  0.04872878
0.04872259
#...1727
#0.04870088


#----------

#now only for pc2... y axis!!!  (push to 8DAI on the upper
(positive) and to 16DAI on the lower (negative...!))


loading_scores_only.tap.data.y <- pca02012023_onlytap$rotation[,2]
protein_scores_only.tap.data.y <-
abs(loading_scores_only.tap.data.y) #proteins that push samples to
the left side of the graph will have a large negative values and
proteins that push samples to
# the right will have a large positive values.
# since we are interested in both sets, of proteins, we will use the
abs() function to sort based on the
# numbers magnitude rather from high to low
protein_score_ranked_only.tap.data.y <-
sort(protein_scores_only.tap.data.y, decreasing=TRUE) #here we sort
the magnitudes of the loading scores from high to low.
top_10_proteins_only.tap.data.y <-
names(protein_score_ranked_only.tap.data.y[1:10]) #now we get the
names of the top 10 proteins with the largest loading score
magnitudes.
top_10_proteins_only.tap.data.y

#top_10_proteins_only.tap.data.y
#[1] "...1277" "...1614" "...779"  "...80"   "...1047" "...1854"
"...586"  "...1235" "...2121" "...1286"

pca02012023_onlytap$rotation[top_10_proteins_only.tap.data.y,2] ##
show the scores (and +/- sign)

#pca02012023_onlytap$rotation[top_10_proteins_only.tap.data.y,2] ##
show the scores (and +/- sign)
#...1277    ...1614     ...779      ...80    ...1047    ...1854
...586    ...1235    ...2121    ...1286
```

```
#0.08095443 0.07861840 0.07780556 0.07750517 0.07718588 0.07616493
0.07575745 0.07544822 0.07525795 0.07476087


top_100_proteins_only.tap.data.y <-
names(protein_score_ranked_only.tap.data.y[1:100]) #now we get the
names of the top 100 proteins with the largest loading score
magnitudes.
top_100_proteins_only.tap.data.y

#top_100_proteins_only.tap.data.y
#[1] "...1277" "...1614" "...779"  "...80"   "...1047" "...1854"
"...586"  "...1235" "...2121" "...1286" "...834"  "...1480" "...885"
#[14] "...1142" "...1182" "...587"  "...1699" "...46"   "...598"
"...894"  "...90"   "...201"  "...215"  "...1607" "...1827"
"...1938"
#[27] "...155"  "...1989" "...2148" "...655"  "...1523" "...265"
"...354"  "...754"  "...1021" "...1328" "...973"  "...1385" "...14"
#[40] "...641"  "...422"  "...1661" "...279"  "...743"  "...1965"
"...2011" "...518"  "...1931" "...544"  "...819"  "...1684"
"...1954"
#[53] "...1820" "...165"  "...815"  "...1120" "...781"  "...64"
"...664"  "...75"   "...590"  "...1617" "...1968" "...1746" "...329"
#[66] "...2087" "...356"  "...899"  "...1354" "...2157" "...1394"
"...1383" "...708"  "...1770" "...1020" "...145"  "...1559" "...453"
#[79] "...382"  "...2107" "...2060" "...880"  "...1782" "...335"
"...381"  "...188"  "...13"   "...311"  "...1052" "...1883"
"...1899"
#[92] "...1865" "...351"  "...1356" "...1704" "...775"  "...278"
"...1668" "...627"  "...772"

pca02012023_onlytap$rotation[top_100_proteins_only.tap.data.y,2]
#pca02012023_onlytap$rotation[top_100_proteins_only.tap.data.y,2]
#...1277     ...1614      ...779       ...80      ...1047     ...1854
...586     ...1235     ...2121     ...1286      ...834
#0.08095443  0.07861840  0.07780556  0.07750517  0.07718588
0.07616493  0.07575745  0.07544822  0.07525795  0.07476087
0.07468039
#...1480      ...885     ...1142     ...1182      ...587     ...1699
...46      ...598      ...894       ...90      ...201
#0.07424452  0.07394512  0.07321742  0.07256040  0.07242906
0.07213084  0.07193225  0.07112411  0.07108765  0.07091678
-0.07042758
#...215     ...1607     ...1827     ...1938      ...155     ...1989
...2148      ...655     ...1523      ...265      ...354
#0.07036442  0.07028003  0.07018610 -0.06997229  0.06921133
0.06843601  0.06834266  0.06723892  0.06682019 -0.06678953
0.06625638
#...754     ...1021     ...1328      ...973     ...1385       ...14
...641      ...422     ...1661      ...279      ...743
#0.06590657 -0.06589207  0.06562749  0.06547706  0.06526612
0.06496701  0.06438061 -0.06430754  0.06409745  0.06401232
0.06388638
#...1965     ...2011      ...518     ...1931      ...544      ...819
```

```
...1684     ...1954     ...1820      ...165      ...815
#-0.06341730  0.06336356 -0.06331140  0.06319392  0.06318949
0.06280857 -0.06270427  0.06225440  0.06215771  0.06200968
0.06182155
#...1120      ...781       ...64      ...664       ...75      ...590
...1617     ...1968     ...1746      ...329     ...2087
#0.06175418  0.06116853 -0.06100336 -0.06088959  0.06076728
0.06071577  0.06019292 -0.06015923  0.05989561  0.05988319
-0.05978720
#...356      ...899     ...1354     ...2157     ...1394     ...1383
...708     ...1770     ...1020      ...145     ...1559
#0.05953369 -0.05951191  0.05945451 -0.05941259 -0.05927610
-0.05895077 -0.05893116 -0.05889845 -0.05882383 -0.05881813
-0.05860316
#...453      ...382     ...2107     ...2060      ...880     ...1782
...335      ...381      ...188       ...13      ...311
#-0.05858730  0.05855390 -0.05831265 -0.05828838 -0.05819102
-0.05818459 -0.05808237 -0.05788447 -0.05740581 -0.05714136
-0.05699044
#...1052     ...1883     ...1899     ...1865      ...351     ...1356
...1704      ...775      ...278     ...1668      ...627
#-0.05695490  0.05693134  0.05681528 -0.05671799  0.05663585
-0.05652936 -0.05628316 -0.05610872 -0.05610826  0.05606884
-0.05597490
#...772
#0.05589484
#---------------------
#-----------------
#---------------------


#----------------------------
#with only data of tap 16DAP (root, shoot, light, dark)


library(readxl)
pca_log_woprot_onlytap16DAI <-
read_excel("pca_r_try_29.12.2022.xlsx",
                                    sheet =
"pca_log_woprot_onlytap16DAI", col_names = FALSE,
                                    col_types = c("text",
                                                  "numeric",
"numeric", "numeric",
                                                  "numeric",
"numeric", "numeric",
                                                  "numeric",
"numeric", "numeric",
                                                  "numeric",
"numeric", "numeric",
                                                  "numeric",
"numeric", "numeric",
                                                  "numeric",
"numeric", "numeric",
                                                  "numeric",
```

"numeric", "numeric",
"numeric",
"numeric", "numeric",
"numeric",
"numeric", "numeric",
"numeric",
"numeric", "numeric",
"numeric",
"numeric", "numeric",
"numeric",
"numeric", "numeric",
"numeric",
"numeric", "numeric",
"numeric",
"numeric", "numeric",
"numeric",
"numeric", "numeric",
"numeric",
"numeric", "numeric",
"numeric",
"numeric", "numeric",
"numeric",
"numeric", "numeric",
"numeric",
"numeric", "numeric",
"numeric",
"numeric", "numeric",
"numeric",
"numeric", "numeric",
"numeric",
"numeric", "numeric",
"numeric",
"numeric", "numeric",
"numeric",
"numeric", "numeric",
"numeric",
"numeric", "numeric",
"numeric",
"numeric", "numeric",
"numeric",
"numeric", "numeric",
"numeric",
"numeric", "numeric",
"numeric",
"numeric", "numeric",
"numeric",
"numeric", "numeric",
"numeric",
"numeric", "numeric",
"numeric",
"numeric", "numeric",
"numeric",

"numeric", "numeric",
"numeric",
"numeric", "numeric",
"numeric",
"numeric", "numeric",
"numeric",
"numeric", "numeric",
"numeric",
"numeric", "numeric",
"numeric",
"numeric", "numeric",
"numeric",
"numeric", "numeric",
"numeric",
"numeric", "numeric",
"numeric",
"numeric", "numeric",
"numeric",
"numeric", "numeric",
"numeric",
"numeric", "numeric",
"numeric",
"numeric", "numeric",
"numeric",
"numeric", "numeric",
"numeric",
"numeric", "numeric",
"numeric",
"numeric", "numeric",
"numeric",
"numeric", "numeric",
"numeric",
"numeric", "numeric",
"numeric",
"numeric", "numeric",
"numeric",
"numeric", "numeric",
"numeric",
"numeric", "numeric",
"numeric",
"numeric", "numeric",
"numeric",
"numeric", "numeric",
"numeric",
"numeric", "numeric",
"numeric",
"numeric", "numeric",
"numeric",
"numeric", "numeric",
"numeric",
"numeric", "numeric",
"numeric",
"numeric", "numeric",
"numeric",

"numeric", "numeric",
"numeric",
"numeric", "numeric",
"numeric",
"numeric", "numeric",
"numeric",
"numeric", "numeric",
"numeric",
"numeric", "numeric",
"numeric",
"numeric", "numeric",
"numeric",
"numeric", "numeric",
"numeric",
"numeric", "numeric",
"numeric",
"numeric", "numeric",
"numeric",
"numeric", "numeric",
"numeric",
"numeric", "numeric",
"numeric",
"numeric", "numeric",
"numeric",
"numeric", "numeric",
"numeric",
"numeric", "numeric",
"numeric",
"numeric", "numeric",
"numeric",
"numeric", "numeric",
"numeric",
"numeric", "numeric",
"numeric",
"numeric", "numeric",
"numeric",
"numeric", "numeric",
"numeric",
"numeric", "numeric",
"numeric",
"numeric", "numeric",
"numeric",
"numeric", "numeric",
"numeric",
"numeric", "numeric",
"numeric",
"numeric", "numeric",
"numeric",
"numeric", "numeric",
"numeric",
"numeric", "numeric",
"numeric",
"numeric", "numeric",
"numeric",

```
"numeric", "numeric",
"numeric",
"numeric", "numeric",
"numeric",
"numeric", "numeric",
"numeric",
"numeric", "numeric",
"numeric",
"numeric", "numeric",
"numeric",
"numeric", "numeric",
"numeric",
"numeric", "numeric",
"numeric",
"numeric", "numeric",
"numeric",
"numeric", "numeric",
"numeric",
"numeric", "numeric",
"numeric",
"numeric", "numeric",
"numeric",
"numeric", "numeric",
"numeric",
"numeric", "numeric",
"numeric",
"numeric", "numeric",
"numeric",
"numeric", "numeric",
"numeric",
"numeric", "numeric",
"numeric",
"numeric", "numeric",
"numeric",
"numeric", "numeric",
"numeric",
"numeric", "numeric",
"numeric",
"numeric", "numeric",
"numeric",
"numeric", "numeric",
"numeric",
"numeric", "numeric",
"numeric",
"numeric", "numeric",
"numeric",
"numeric", "numeric",
"numeric",
"numeric", "numeric",
"numeric",
"numeric", "numeric",
"numeric",
"numeric", "numeric",
"numeric",
```

```
"numeric", "numeric",
"numeric",
"numeric", "numeric",
"numeric",
"numeric", "numeric",
"numeric",
"numeric", "numeric",
"numeric",
"numeric", "numeric",
"numeric",
"numeric", "numeric",
"numeric",
"numeric", "numeric",
"numeric",
"numeric", "numeric",
"numeric",
"numeric", "numeric",
"numeric",
"numeric", "numeric",
"numeric",
"numeric", "numeric",
"numeric",
"numeric", "numeric",
"numeric",
"numeric", "numeric",
"numeric",
"numeric", "numeric",
"numeric",
"numeric", "numeric",
"numeric",
"numeric", "numeric",
"numeric",
"numeric", "numeric",
"numeric",
"numeric", "numeric",
"numeric",
"numeric", "numeric",
"numeric",
"numeric", "numeric",
"numeric",
"numeric", "numeric",
"numeric",
"numeric", "numeric",
"numeric",
"numeric", "numeric",
"numeric",
"numeric", "numeric",
"numeric",
"numeric", "numeric",
"numeric",
"numeric", "numeric",
"numeric",
"numeric", "numeric",
"numeric",
```

"numeric", "numeric",
"numeric",
"numeric", "numeric",
"numeric",
"numeric", "numeric",
"numeric",
"numeric", "numeric",
"numeric",
"numeric", "numeric",
"numeric",
"numeric", "numeric",
"numeric",
"numeric", "numeric",
"numeric",
"numeric", "numeric",
"numeric",
"numeric", "numeric",
"numeric",
"numeric", "numeric",
"numeric",
"numeric", "numeric",
"numeric",
"numeric", "numeric",
"numeric",
"numeric", "numeric",
"numeric",
"numeric", "numeric",
"numeric",
"numeric", "numeric",
"numeric",
"numeric", "numeric",
"numeric",
"numeric", "numeric",
"numeric",
"numeric", "numeric",
"numeric",
"numeric", "numeric",
"numeric",
"numeric", "numeric",
"numeric",
"numeric", "numeric",
"numeric",
"numeric", "numeric",
"numeric",
"numeric", "numeric",
"numeric",
"numeric", "numeric",
"numeric",
"numeric", "numeric",
"numeric",
"numeric", "numeric",
"numeric",
"numeric", "numeric",
"numeric",

"numeric", "numeric",
"numeric",
"numeric", "numeric",
"numeric",
"numeric", "numeric",
"numeric",
"numeric", "numeric",
"numeric",
"numeric", "numeric",
"numeric",
"numeric", "numeric",
"numeric",
"numeric", "numeric",
"numeric",
"numeric", "numeric",
"numeric",
"numeric", "numeric",
"numeric",
"numeric", "numeric",
"numeric",
"numeric", "numeric",
"numeric",
"numeric", "numeric",
"numeric",
"numeric", "numeric",
"numeric",
"numeric", "numeric",
"numeric",
"numeric", "numeric",
"numeric",
"numeric", "numeric",
"numeric",
"numeric", "numeric",
"numeric",
"numeric", "numeric",
"numeric",
"numeric", "numeric",
"numeric",
"numeric", "numeric",
"numeric",
"numeric", "numeric",
"numeric",
"numeric", "numeric",
"numeric",
"numeric", "numeric",
"numeric",
"numeric", "numeric",
"numeric",
"numeric", "numeric",
"numeric",
"numeric", "numeric",
"numeric",
"numeric", "numeric",
"numeric",

"numeric", "numeric",
"numeric",
"numeric", "numeric",
"numeric",
"numeric", "numeric",
"numeric",
"numeric", "numeric",
"numeric",
"numeric", "numeric",
"numeric",
"numeric", "numeric",
"numeric",
"numeric", "numeric",
"numeric",
"numeric", "numeric",
"numeric",
"numeric", "numeric",
"numeric",
"numeric", "numeric",
"numeric",
"numeric", "numeric",
"numeric",
"numeric", "numeric",
"numeric",
"numeric", "numeric",
"numeric",
"numeric", "numeric",
"numeric",
"numeric", "numeric",
"numeric",
"numeric", "numeric",
"numeric",
"numeric", "numeric",
"numeric",
"numeric", "numeric",
"numeric",
"numeric", "numeric",
"numeric",
"numeric", "numeric",
"numeric",
"numeric", "numeric",
"numeric",
"numeric", "numeric",
"numeric",
"numeric", "numeric",
"numeric",
"numeric", "numeric",
"numeric",
"numeric", "numeric",
"numeric",
"numeric", "numeric",
"numeric",
"numeric", "numeric",
"numeric",

"numeric", "numeric",
"numeric",
"numeric", "numeric",
"numeric",
"numeric", "numeric",
"numeric",
"numeric", "numeric",
"numeric",
"numeric", "numeric",
"numeric",
"numeric", "numeric",
"numeric",
"numeric", "numeric",
"numeric",
"numeric", "numeric",
"numeric",
"numeric", "numeric",
"numeric",
"numeric", "numeric",
"numeric",
"numeric", "numeric",
"numeric",
"numeric", "numeric",
"numeric",
"numeric", "numeric",
"numeric",
"numeric", "numeric",
"numeric",
"numeric", "numeric",
"numeric",
"numeric", "numeric",
"numeric",
"numeric", "numeric",
"numeric",
"numeric", "numeric",
"numeric",
"numeric", "numeric",
"numeric",
"numeric", "numeric",
"numeric",
"numeric", "numeric",
"numeric",
"numeric", "numeric",
"numeric",
"numeric", "numeric",
"numeric",
"numeric", "numeric",
"numeric",
"numeric", "numeric",
"numeric",
"numeric", "numeric",
"numeric",
"numeric", "numeric",
"numeric",

"numeric", "numeric",
"numeric",
"numeric", "numeric",
"numeric",
"numeric", "numeric",
"numeric",
"numeric", "numeric",
"numeric",
"numeric", "numeric",
"numeric",
"numeric", "numeric",
"numeric",
"numeric", "numeric",
"numeric",
"numeric", "numeric",
"numeric",
"numeric", "numeric",
"numeric",
"numeric", "numeric",
"numeric",
"numeric", "numeric",
"numeric",
"numeric", "numeric",
"numeric",
"numeric", "numeric",
"numeric",
"numeric", "numeric",
"numeric",
"numeric", "numeric",
"numeric",
"numeric", "numeric",
"numeric",
"numeric", "numeric",
"numeric",
"numeric", "numeric",
"numeric",
"numeric", "numeric",
"numeric",
"numeric", "numeric",
"numeric",
"numeric", "numeric",
"numeric",
"numeric", "numeric",
"numeric",
"numeric", "numeric",
"numeric",
"numeric", "numeric",
"numeric",
"numeric", "numeric",
"numeric",
"numeric", "numeric",
"numeric",
"numeric", "numeric",
"numeric",

```
"numeric", "numeric",
"numeric",
"numeric", "numeric",
"numeric",
"numeric", "numeric",
"numeric",
"numeric", "numeric",
"numeric",
"numeric", "numeric",
"numeric",
"numeric", "numeric",
"numeric",
"numeric", "numeric",
"numeric",
"numeric", "numeric",
"numeric",
"numeric", "numeric",
"numeric",
"numeric", "numeric",
"numeric",
"numeric", "numeric",
"numeric",
"numeric", "numeric",
"numeric",
"numeric", "numeric",
"numeric",
"numeric", "numeric",
"numeric",
"numeric", "numeric",
"numeric",
"numeric", "numeric",
"numeric",
"numeric", "numeric",
"numeric",
"numeric", "numeric",
"numeric",
"numeric", "numeric",
"numeric",
"numeric", "numeric",
"numeric",
"numeric", "numeric",
"numeric",
"numeric", "numeric",
"numeric",
"numeric", "numeric",
"numeric",
"numeric", "numeric",
"numeric",
"numeric", "numeric",
"numeric",
"numeric", "numeric",
"numeric",
"numeric", "numeric",
"numeric",
```

"numeric", "numeric",
"numeric",
"numeric", "numeric",
"numeric",
"numeric", "numeric",
"numeric",
"numeric", "numeric",
"numeric",
"numeric", "numeric",
"numeric",
"numeric", "numeric",
"numeric",
"numeric", "numeric",
"numeric",
"numeric", "numeric",
"numeric",
"numeric", "numeric",
"numeric",
"numeric", "numeric",
"numeric",
"numeric", "numeric",
"numeric",
"numeric", "numeric",
"numeric",
"numeric", "numeric",
"numeric",
"numeric", "numeric",
"numeric",
"numeric", "numeric",
"numeric",
"numeric", "numeric",
"numeric",
"numeric", "numeric",
"numeric",
"numeric", "numeric",
"numeric",
"numeric", "numeric",
"numeric",
"numeric", "numeric",
"numeric",
"numeric", "numeric",
"numeric",
"numeric", "numeric",
"numeric",
"numeric", "numeric",
"numeric",
"numeric", "numeric",
"numeric",
"numeric", "numeric",
"numeric",
"numeric", "numeric",
"numeric",
"numeric", "numeric",
"numeric",

"numeric", "numeric",
"numeric",
"numeric", "numeric",
"numeric",
"numeric", "numeric",
"numeric",
"numeric", "numeric",
"numeric",
"numeric", "numeric",
"numeric",
"numeric", "numeric",
"numeric",
"numeric", "numeric",
"numeric",
"numeric", "numeric",
"numeric",
"numeric", "numeric",
"numeric",
"numeric", "numeric",
"numeric",
"numeric", "numeric",
"numeric",
"numeric", "numeric",
"numeric",
"numeric", "numeric",
"numeric",
"numeric", "numeric",
"numeric",
"numeric", "numeric",
"numeric",
"numeric", "numeric",
"numeric",
"numeric", "numeric",
"numeric",
"numeric", "numeric",
"numeric",
"numeric", "numeric",
"numeric",
"numeric", "numeric",
"numeric",
"numeric", "numeric",
"numeric",
"numeric", "numeric",
"numeric",
"numeric", "numeric",
"numeric",
"numeric", "numeric",
"numeric",
"numeric", "numeric",
"numeric",
"numeric", "numeric",
"numeric",
"numeric", "numeric",
"numeric",

"numeric", "numeric",
"numeric", "numeric",
"numeric", "numeric",
"numeric", "numeric",
"numeric", "numeric",
"numeric", "numeric",
"numeric", "numeric",
"numeric", "numeric",
"numeric", "numeric",
"numeric", "numeric",
"numeric", "numeric",
"numeric", "numeric",
"numeric", "numeric",
"numeric", "numeric",
"numeric", "numeric",
"numeric", "numeric",
"numeric", "numeric",
"numeric", "numeric",
"numeric", "numeric",
"numeric", "numeric",
"numeric", "numeric",
"numeric", "numeric",
"numeric", "numeric",
"numeric", "numeric",
"numeric", "numeric",
"numeric", "numeric",
"numeric", "numeric",
"numeric",
"numeric",
"numeric",
"numeric",
"numeric",
"numeric",
"numeric",
"numeric",
"numeric",
"numeric",
"numeric",
"numeric",
"numeric",
"numeric",
"numeric",
"numeric",
"numeric",
"numeric",
"numeric",
"numeric",
"numeric",
"numeric",
"numeric",
"numeric",
"numeric",
"numeric",
"numeric",

```
"numeric", "numeric",
"numeric",
"numeric", "numeric",
"numeric",
"numeric", "numeric",
"numeric",
"numeric", "numeric",
"numeric",
"numeric", "numeric",
"numeric",
"numeric", "numeric",
"numeric",
"numeric", "numeric",
"numeric",
"numeric", "numeric",
"numeric",
"numeric", "numeric",
"numeric",
"numeric", "numeric",
"numeric",
"numeric", "numeric",
"numeric",
"numeric", "numeric",
"numeric",
"numeric", "numeric",
"numeric",
"numeric", "numeric",
"numeric",
"numeric", "numeric",
"numeric",
"numeric", "numeric",
"numeric",
"numeric", "numeric",
"numeric",
"numeric", "numeric",
"numeric",
"numeric", "numeric",
"numeric",
"numeric", "numeric",
"numeric",
"numeric", "numeric",
"numeric",
"numeric", "numeric",
"numeric",
"numeric", "numeric",
"numeric",
"numeric", "numeric",
"numeric",
"numeric", "numeric",
"numeric",
"numeric", "numeric",
"numeric",
"numeric", "numeric",
"numeric",
```

"numeric", "numeric",
"numeric",
"numeric", "numeric",
"numeric",
"numeric", "numeric",
"numeric",
"numeric", "numeric",
"numeric",
"numeric", "numeric",
"numeric",
"numeric", "numeric",
"numeric",
"numeric", "numeric",
"numeric",
"numeric", "numeric",
"numeric",
"numeric", "numeric",
"numeric",
"numeric", "numeric",
"numeric",
"numeric", "numeric",
"numeric",
"numeric", "numeric",
"numeric",
"numeric", "numeric",
"numeric",
"numeric", "numeric",
"numeric",
"numeric", "numeric",
"numeric",
"numeric", "numeric",
"numeric",
"numeric", "numeric",
"numeric",
"numeric", "numeric",
"numeric",
"numeric", "numeric",
"numeric",
"numeric", "numeric",
"numeric",
"numeric", "numeric",
"numeric",
"numeric", "numeric",
"numeric",
"numeric", "numeric",
"numeric",
"numeric", "numeric",
"numeric",
"numeric", "numeric",
"numeric",
"numeric", "numeric",
"numeric",
"numeric", "numeric",
"numeric",

"numeric", "numeric",
"numeric", "numeric",
"numeric", "numeric",
"numeric", "numeric",
"numeric", "numeric",
"numeric", "numeric",
"numeric", "numeric",
"numeric", "numeric",
"numeric", "numeric",
"numeric", "numeric",
"numeric", "numeric",
"numeric", "numeric",
"numeric", "numeric",
"numeric", "numeric",
"numeric", "numeric",
"numeric", "numeric",
"numeric", "numeric",
"numeric", "numeric",
"numeric", "numeric",
"numeric", "numeric",
"numeric", "numeric",
"numeric", "numeric",
"numeric", "numeric",
"numeric", "numeric",
"numeric", "numeric",
"numeric", "numeric",
"numeric", "numeric",
"numeric",
"numeric",
"numeric",
"numeric",
"numeric",
"numeric",
"numeric",
"numeric",
"numeric",
"numeric",
"numeric",
"numeric",
"numeric",
"numeric",
"numeric",
"numeric",
"numeric",
"numeric",
"numeric",
"numeric",
"numeric",
"numeric",
"numeric",
"numeric",
"numeric",
"numeric",
"numeric",

"numeric", "numeric",
"numeric",
"numeric", "numeric",
"numeric",
"numeric", "numeric",
"numeric",
"numeric", "numeric",
"numeric",
"numeric", "numeric",
"numeric",
"numeric", "numeric",
"numeric",
"numeric", "numeric",
"numeric",
"numeric", "numeric",
"numeric",
"numeric", "numeric",
"numeric",
"numeric", "numeric",
"numeric",
"numeric", "numeric",
"numeric",
"numeric", "numeric",
"numeric",
"numeric", "numeric",
"numeric",
"numeric", "numeric",
"numeric",
"numeric", "numeric",
"numeric",
"numeric", "numeric",
"numeric",
"numeric", "numeric",
"numeric",
"numeric", "numeric",
"numeric",
"numeric", "numeric",
"numeric",
"numeric", "numeric",
"numeric",
"numeric", "numeric",
"numeric",
"numeric", "numeric",
"numeric",
"numeric", "numeric",
"numeric",
"numeric", "numeric",
"numeric",
"numeric", "numeric",
"numeric",
"numeric", "numeric",
"numeric",
"numeric", "numeric",
"numeric",

"numeric", "numeric",
"numeric",
"numeric", "numeric",
"numeric",
"numeric", "numeric",
"numeric",
"numeric", "numeric",
"numeric",
"numeric", "numeric",
"numeric",
"numeric", "numeric",
"numeric",
"numeric", "numeric",
"numeric",
"numeric", "numeric",
"numeric",
"numeric", "numeric",
"numeric",
"numeric", "numeric",
"numeric",
"numeric", "numeric",
"numeric",
"numeric", "numeric",
"numeric",
"numeric", "numeric",
"numeric",
"numeric", "numeric",
"numeric",
"numeric", "numeric",
"numeric",
"numeric", "numeric",
"numeric",
"numeric", "numeric",
"numeric",
"numeric", "numeric",
"numeric",
"numeric", "numeric",
"numeric",
"numeric", "numeric",
"numeric",
"numeric", "numeric",
"numeric",
"numeric", "numeric",
"numeric",
"numeric", "numeric",
"numeric",
"numeric", "numeric",
"numeric",
"numeric", "numeric",
"numeric",
"numeric", "numeric",
"numeric",
"numeric", "numeric",
"numeric",

```
"numeric", "numeric",
"numeric", "numeric",
"numeric", "numeric",
"numeric", "numeric",
"numeric", "numeric",
"numeric", "numeric",
"numeric", "numeric",
"numeric", "numeric",
"numeric", "numeric",
"numeric", "numeric",
"numeric", "numeric",
"numeric", "numeric",
"numeric", "numeric",
"numeric", "numeric",
"numeric", "numeric",
"numeric", "numeric",
"numeric", "numeric",
"numeric", "numeric",
"numeric", "numeric",
"numeric", "numeric",
"numeric", "numeric",
"numeric", "numeric",
"numeric", "numeric",
"numeric", "numeric",
"numeric", "numeric",
"numeric", "numeric",
"numeric", "numeric",
"numeric",
"numeric",
"numeric",
"numeric",
"numeric",
"numeric",
"numeric",
"numeric",
"numeric",
"numeric",
"numeric",
"numeric",
"numeric",
"numeric",
"numeric",
"numeric",
"numeric",
"numeric",
"numeric",
"numeric",
"numeric",
"numeric",
"numeric",
"numeric",
"numeric",
"numeric",
"numeric",
```

"numeric", "numeric",
"numeric",
"numeric", "numeric",
"numeric",
"numeric", "numeric",
"numeric",
"numeric", "numeric",
"numeric",
"numeric", "numeric",
"numeric",
"numeric", "numeric",
"numeric",
"numeric", "numeric",
"numeric",
"numeric", "numeric",
"numeric",
"numeric", "numeric",
"numeric",
"numeric", "numeric",
"numeric",
"numeric", "numeric",
"numeric",
"numeric", "numeric",
"numeric",
"numeric", "numeric",
"numeric",
"numeric", "numeric",
"numeric",
"numeric", "numeric",
"numeric",
"numeric", "numeric",
"numeric",
"numeric", "numeric",
"numeric",
"numeric", "numeric",
"numeric",
"numeric", "numeric",
"numeric",
"numeric", "numeric",
"numeric",
"numeric", "numeric",
"numeric",
"numeric", "numeric",
"numeric",
"numeric", "numeric",
"numeric",
"numeric", "numeric",
"numeric",
"numeric", "numeric",
"numeric",
"numeric", "numeric",
"numeric",
"numeric", "numeric",
"numeric",

"numeric", "numeric",
"numeric",
"numeric", "numeric",
"numeric",
"numeric", "numeric",
"numeric",
"numeric", "numeric",
"numeric",
"numeric", "numeric",
"numeric",
"numeric", "numeric",
"numeric",
"numeric", "numeric",
"numeric",
"numeric", "numeric",
"numeric",
"numeric", "numeric",
"numeric",
"numeric", "numeric",
"numeric",
"numeric", "numeric",
"numeric",
"numeric", "numeric",
"numeric",
"numeric", "numeric",
"numeric",
"numeric", "numeric",
"numeric",
"numeric", "numeric",
"numeric",
"numeric", "numeric",
"numeric",
"numeric", "numeric",
"numeric",
"numeric", "numeric",
"numeric",
"numeric", "numeric",
"numeric",
"numeric", "numeric",
"numeric",
"numeric", "numeric",
"numeric",
"numeric", "numeric",
"numeric",
"numeric", "numeric",
"numeric",
"numeric", "numeric",
"numeric",
"numeric", "numeric",
"numeric",
"numeric", "numeric",
"numeric",
"numeric", "numeric",
"numeric",

```
"numeric", "numeric",
"numeric", "numeric",
"numeric", "numeric",
"numeric", "numeric",
"numeric", "numeric",
"numeric", "numeric",
"numeric", "numeric",
"numeric", "numeric",
"numeric", "numeric",
"numeric", "numeric",
"numeric", "numeric",
"numeric", "numeric",
"numeric", "numeric",
"numeric", "numeric",
"numeric", "numeric",
"numeric", "numeric",
"numeric", "numeric",
"numeric", "numeric",
"numeric", "numeric",
"numeric", "numeric",
"numeric", "numeric",
"numeric", "numeric",
"numeric", "numeric",
"numeric", "numeric",
"numeric", "numeric",
"numeric", "numeric",
"numeric", "numeric",
"numeric",
"numeric",
"numeric",
"numeric",
"numeric",
"numeric",
"numeric",
"numeric",
"numeric",
"numeric",
"numeric",
"numeric",
"numeric",
"numeric",
"numeric",
"numeric",
"numeric",
"numeric",
"numeric",
"numeric",
"numeric",
"numeric",
"numeric",
"numeric",
"numeric",
"numeric",
"numeric",
```

"numeric", "numeric",
"numeric",
"numeric", "numeric",
"numeric",
"numeric", "numeric",
"numeric",
"numeric", "numeric",
"numeric",
"numeric", "numeric",
"numeric",
"numeric", "numeric",
"numeric",
"numeric", "numeric",
"numeric",
"numeric", "numeric",
"numeric",
"numeric", "numeric",
"numeric",
"numeric", "numeric",
"numeric",
"numeric", "numeric",
"numeric",
"numeric", "numeric",
"numeric",
"numeric", "numeric",
"numeric",
"numeric", "numeric",
"numeric",
"numeric", "numeric",
"numeric",
"numeric", "numeric",
"numeric",
"numeric", "numeric",
"numeric",
"numeric", "numeric",
"numeric",
"numeric", "numeric",
"numeric",
"numeric", "numeric",
"numeric",
"numeric", "numeric",
"numeric",
"numeric", "numeric",
"numeric",
"numeric", "numeric",
"numeric",
"numeric", "numeric",
"numeric",
"numeric", "numeric",
"numeric",
"numeric", "numeric",
"numeric",
"numeric", "numeric",
"numeric",

"numeric", "numeric",
"numeric", "numeric",
"numeric", "numeric",
"numeric", "numeric",
"numeric", "numeric",
"numeric", "numeric",
"numeric", "numeric",
"numeric", "numeric",
"numeric", "numeric",
"numeric", "numeric",
"numeric", "numeric",
"numeric", "numeric",
"numeric", "numeric",
"numeric", "numeric",
"numeric", "numeric",
"numeric", "numeric",
"numeric", "numeric",
"numeric", "numeric",
"numeric", "numeric",
"numeric", "numeric",
"numeric", "numeric",
"numeric", "numeric",
"numeric", "numeric",
"numeric", "numeric",
"numeric", "numeric",
"numeric", "numeric",
"numeric", "numeric",
"numeric",
"numeric",
"numeric",
"numeric",
"numeric",
"numeric",
"numeric",
"numeric",
"numeric",
"numeric",
"numeric",
"numeric",
"numeric",
"numeric",
"numeric",
"numeric",
"numeric",
"numeric",
"numeric",
"numeric",
"numeric",
"numeric",
"numeric",
"numeric",
"numeric",
"numeric",
"numeric",

"numeric", "numeric",
"numeric", "numeric",
"numeric", "numeric",
"numeric", "numeric",
"numeric", "numeric",
"numeric", "numeric",
"numeric", "numeric",
"numeric", "numeric",
"numeric", "numeric",
"numeric", "numeric",
"numeric", "numeric",
"numeric", "numeric",
"numeric", "numeric",
"numeric", "numeric",
"numeric", "numeric",
"numeric", "numeric",
"numeric", "numeric",
"numeric", "numeric",
"numeric", "numeric",
"numeric", "numeric",
"numeric", "numeric",
"numeric", "numeric",
"numeric", "numeric",
"numeric", "numeric",
"numeric", "numeric",
"numeric", "numeric",
"numeric", "numeric",
"numeric",
"numeric",
"numeric",
"numeric",
"numeric",
"numeric",
"numeric",
"numeric",
"numeric",
"numeric",
"numeric",
"numeric",
"numeric",
"numeric",
"numeric",
"numeric",
"numeric",
"numeric",
"numeric",
"numeric",
"numeric",
"numeric",
"numeric",
"numeric",
"numeric",
"numeric",
"numeric",

```
"numeric", "numeric",
                                                   "numeric",
"numeric", "numeric",
                                                   "numeric",
"numeric", "numeric",
                                                   "numeric",
"numeric", "numeric",
                                                   "numeric",
"numeric", "numeric",
                                                   "numeric",
"numeric", "numeric",
                                                   "numeric",
"numeric", "numeric",
                                                   "numeric",
"numeric", "numeric",
                                                   "numeric",
"numeric", "numeric",
                                                   "numeric",
"numeric", "numeric",
                                                   "numeric",
"numeric", "numeric",
                                                   "numeric"))

View(pca_log_woprot_onlytap16DAI)
pca_log_woprot_onlytap16DAI
pca_log_woprot_onlytap16DAI_matrix<-
as.matrix(pca_log_woprot_onlytap16DAI)
#pca <- prcomp((pca_log_woprot), scale=TRUE)
pca_log_woprot_onlytap16DAI_matrix



pca_log_woprot_onlytap16DAI_new <- pca_log_woprot_onlytap16DAI[ ,
unlist(lapply(pca_log_woprot_onlytap16DAI,             # Remove non-
numeric columns

is.numeric))]
pca_log_woprot_onlytap16DAI_new
pca_03012023_onlytap16DAI <- prcomp(pca_log_woprot_onlytap16DAI_new)
#worked!!!!! (https://statisticsglobe.com/r-error-colmeans-x-must-
be-numeric)
plot(pca_03012023_onlytap16DAI$x[,1],
pca_03012023_onlytap16DAI$x[,2]) #worked!!!!   https://
www.youtube.com/watch?v=0Jp4gsfOLMs&t=153s
pca_03012023_onlytap16DAI.var <-pca_03012023_onlytap16DAI$sdev^2
pca_03012023_onlytap16DAI.var.per <-
round(pca_03012023_onlytap16DAI.var/
sum(pca_03012023_onlytap16DAI.var)*100,1)
barplot(pca_03012023_onlytap16DAI.var.per, main="Scree Plot",
xlab="Principal Component", ylab="Percent Variation") # i have PCs
for every sample....!

pca_03012023_onlytap16DAI.var.per
```

```
#pca plot only tap



group_onlytap16DAI <- c("16DAI_tap_DR", "16DAI_tap_DR",
"16DAI_tap_DR",
                   "16DAI_tap_DR",  "16DAI_tap_DS",  "16DAI_tap_DS",
"16DAI_tap_DS",  "16DAI_tap_LR",  "16DAI_tap_LR",
                   "16DAI_tap_LR",  "16DAI_tap_LS",  "16DAI_tap_LS",
"16DAI_tap_LS",  "16DAI_tap_LS")
group_onlytap16DAI


plot(pca_03012023_onlytap16DAI$x[,1],pca_03012023_onlytap16DAI$x[,2]
,
     col=factor(group_onlytap16DAI),main="PCA plot 16DAI tap - light
- dark",
     pch=19, xlab="PC1 (72.5%)",
     ylab="PC2 (15.5%)")

library(ggfortify)
autoplot(pca_03012023_onlytap16DAI, data =
pca_log_woprot_onlytap16DAI_new, main="PCA plot 16DAI tap - light -
dark", shape = FALSE, label.size = 3)# hier andere dastellweise mit
Zahlen als beschriftung... 1-53

#die top 10 und top 100 proteine...
# für die x achse... durck nach root oder shoot...

loading_scores_tap16DAI <- pca_03012023_onlytap16DAI$rotation[,1]
protein_scores_tap16DAI <- abs(loading_scores_tap16DAI) #proteins
that push samples to the left side of the graph will have a large
negative values and proteins that push samples to
# the right will have a large positive values.
# since we are interested in both sets, of proteins, we will use the
abs() function to sort based on the
# numbers magnitude rather from high to low
protein_score_ranked_tap16DAI <- sort(protein_scores_tap16DAI,
decreasing=TRUE) #here we sort the magnitudes of the loading scores
from high to low.
top_10_proteins_tap16DAI <-
names(protein_score_ranked_tap16DAI[1:10]) #now we get the names of
the top 10 proteins with the largest loading score magnitudes.
top_10_proteins_tap16DAI

pca_03012023_onlytap16DAI$rotation[top_10_proteins_tap16DAI,1] ##
show the scores (and +/- sign)


#top_10_proteins_tap16DAI
#[1] "...2021" "...1714" "...319"  "...1587" "...1929" "...1393"
"...238"  "...1505" "...913"  "...1629"
#> pca_03012023_onlytap16DAI$rotation[top_10_proteins_tap16DAI,1] ##
show the scores (and +/- sign)
```

```
#...2021     ...1714      ...319     ...1587     ...1929     ...1393
...238     ...1505      ...913     ...1629
#0.05365639  0.05308743  0.05082510  0.05064362  0.04992053
-0.04989495  0.04964956  0.04953807  0.04943502  0.04942109


#---

top_100_proteins_tap16DAI <-
names(protein_score_ranked_tap16DAI[1:100]) #now we get the names of
the top 10 proteins with the largest loading score magnitudes.
top_100_proteins_tap16DAI

pca_03012023_onlytap16DAI$rotation[top_100_proteins_tap16DAI,1] ##
show the scores (and +/- sign)


#top_100_proteins_tap16DAI
#[1] "...2021" "...1714" "...319"  "...1587" "...1929" "...1393"
"...238"  "...1505" "...913"  "...1629" "...723"  "...1824" "...718"
#[14] "...1141" "...1969" "...982"  "...1183" "...265"  "...1332"
"...196"  "...154"  "...1550" "...1233" "...902"  "...2141"
"...1021"
#[27] "...949"  "...1763" "...374"  "...82"   "...1272" "...1461"
"...506"  "...667"  "...1840" "...621"  "...1355" "...1172" "...931"
#[40] "...919"  "...1341" "...295"  "...70"   "...1100" "...1644"
"...1621" "...2071" "...17"   "...2138" "...175"  "...1307" "...904"
#[53] "...1606" "...1575" "...1122" "...5"    "...439"  "...805"
"...840"  "...630"  "...1033" "...2156" "...1611" "...829"  "...728"
#[66] "...502"  "...2155" "...1859" "...518"  "...2077" "...1574"
"...845"  "...1831" "...105"  "...366"  "...2069" "...62"   "...208"
#[79] "...61"   "...149"  "...1478" "...1567" "...1214" "...1684"
"...1829" "...1835" "...1512" "...29"   "...824"  "...784"  "...135"
#[92] "...910"  "...923"  "...1732" "...48"   "...739"  "...394"
"...200"  "...2158" "...929"
#> pca_03012023_onlytap16DAI$rotation[top_100_proteins_tap16DAI,1]
## show the scores (and +/- sign)
#...2021     ...1714      ...319     ...1587     ...1929     ...1393
...238     ...1505      ...913     ...1629      ...723
#0.05365639  0.05308743  0.05082510  0.05064362  0.04992053
-0.04989495  0.04964956  0.04953807  0.04943502  0.04942109
0.04937223
#...1824      ...718     ...1141     ...1969      ...982     ...1183
...265     ...1332      ...196      ...154     ...1550
#0.04917228 -0.04909604  0.04864161  0.04861136  0.04858774
0.04858319  0.04855117 -0.04850756  0.04849072  0.04843534
0.04833739
#...1233      ...902     ...2141     ...1021      ...949     ...1763
...374       ...82     ...1272     ...1461      ...506
#0.04825601 -0.04816747 -0.04792554  0.04788675  0.04782381
0.04776317  0.04772711  0.04772605  0.04769007  0.04768000
0.04766847
#...667     ...1840      ...621     ...1355     ...1172      ...931
...919     ...1341      ...295       ...70     ...1100
```

```
#0.04756207  0.04746194  0.04741550  0.04723356  0.04721589
-0.04715471  0.04711400  0.04709954  0.04708964 -0.04700677
0.04693027
#...1644     ...1621     ...2071       ...17     ...2138      ...175
...1307      ...904     ...1606     ...1575     ...1122
#0.04689442  0.04688826  0.04688818 -0.04687735  0.04673735
0.04670641 -0.04663857  0.04655462  0.04654982  0.04653555
0.04653176
#...5      ...439      ...805      ...840      ...630     ...1033
...2156     ...1611      ...829      ...728      ...502
#0.04648165  0.04643719  0.04641609  0.04639773  0.04639209
0.04634204  0.04627806  0.04626514  0.04613634 -0.04609646
-0.04609380
#...2155     ...1859      ...518     ...2077     ...1574      ...845
...1831      ...105      ...366     ...2069       ...62
#0.04607151  0.04607012  0.04602219 -0.04601642  0.04598852
0.04595713  0.04589856  0.04589763  0.04587792  0.04587468
0.04577252
#...208       ...61      ...149     ...1478     ...1567     ...1214
...1684     ...1829     ...1835     ...1512       ...29
#0.04568356  0.04566704  0.04566031  0.04565275  0.04564539
0.04562908  0.04557599  0.04557590  0.04550160  0.04544585
0.04543247
#...824      ...784      ...135      ...910      ...923     ...1732
...48      ...739      ...394      ...200     ...2158
#0.04542604  0.04538554  0.04525377 -0.04521452  0.04520995
0.04515701 -0.04508364  0.04508358  0.04508046 -0.04507974
0.04507166
#...929
#-0.04504761

#---



#die top 10 und top 100 proteine...
# für die y achse... durck nach light  oder dark...


loading_scores_tap16DAI_y <- pca_03012023_onlytap16DAI$rotation[,2]
protein_scores_tap16DAI_y <- abs(loading_scores_tap16DAI_y)
#proteins that push samples to the left side of the graph will have
a large negative values and proteins that push samples to
# the right will have a large positive values.
# since we are interested in both sets, of proteins, we will use the
abs() function to sort based on the
# numbers magnitude rather from high to low
protein_score_ranked_tap16DAI_y <- sort(protein_scores_tap16DAI_y,
decreasing=TRUE) #here we sort the magnitudes of the loading scores
from high to low.
top_10_proteins_tap16DAI_y <-
names(protein_score_ranked_tap16DAI_y[1:10]) #now we get the names
of the top 10 proteins with the largest loading score magnitudes.
top_10_proteins_tap16DAI_y
```

```
pca_03012023_onlytap16DAI$rotation[top_10_proteins_tap16DAI_y,2] ## 
show the scores (and +/- sign)


#top_10_proteins_tap16DAI_y
#[1] "...1485" "...40"   "...370"  "...1065" "...185"  "...2150"
"...1388" "...539"  "...1071" "...1258"
#pca_03012023_onlytap16DAI$rotation[top_10_proteins_tap16DAI_y,2] ##
show the scores (and +/- sign)
#...1485       ...40      ...370     ...1065      ...185     ...2150
...1388      ...539     ...1071     ...1258
#0.07890395 -0.07467629 -0.07269262 -0.07268634 -0.07185008
-0.07127137 -0.06976783  0.06911840 -0.06906174 -0.06892502



top_100_proteins_tap16DAI_y <-
names(protein_score_ranked_tap16DAI_y[1:100]) #now we get the names
of the top 10 proteins with the largest loading score magnitudes.
top_100_proteins_tap16DAI_y

pca_03012023_onlytap16DAI$rotation[top_100_proteins_tap16DAI_y,2] ##
show the scores (and +/- sign)

#top_100_proteins_tap16DAI_y
#[1] "...1485" "...40"   "...370"  "...1065" "...185"  "...2150"
"...1388" "...539"  "...1071" "...1258" "...260"  "...730"
"...1855"
#[14] "...1144" "...1050" "...726"  "...1870" "...1456" "...428"
"...801"  "...1026" "...116"  "...460"  "...1330" "...1192"
"...1810"
#[27] "...2146" "...595"  "...856"  "...1837" "...958"  "...302"
"...1460" "...150"  "...701"  "...2121" "...2074" "...1222" "...779"
#[40] "...1699" "...1772" "...617"  "...497"  "...133"  "...790"
"...2041" "...944"  "...596"  "...846"  "...2127" "...1998" "...288"
#[53] "...1819" "...338"  "...1648" "...1114" "...578"  "...1962"
"...2064" "...350"  "...1988" "...1488" "...174"  "...1204" "...236"
#[66] "...1277" "...989"  "...1643" "...449"  "...1996" "...1566"
"...346"  "...985"  "...413"  "...1295" "...1228" "...324"
"...1934"
#[79] "...213"  "...1730" "...294"  "...1610" "...1235" "...920"
"...999"  "...122"  "...429"  "...657"  "...1989" "...481"
"...1940"
#[92] "...2133" "...328"  "...1206" "...1854" "...32"   "...412"
"...1265" "...1784" "...1509"
#> pca_03012023_onlytap16DAI$rotation[top_100_proteins_tap16DAI_y,2]
## show the scores (and +/- sign)
#...1485       ...40      ...370     ...1065      ...185     ...2150
...1388      ...539     ...1071     ...1258      ...260
#0.07890395 -0.07467629 -0.07269262 -0.07268634 -0.07185008
-0.07127137 -0.06976783  0.06911840 -0.06906174 -0.06892502
-0.06860981
#...730     ...1855     ...1144     ...1050      ...726     ...1870
```

```
...1456      ...428      ...801     ...1026      ...116
#-0.06819629 -0.06815311 -0.06800391  0.06789239 -0.06786549
-0.06770657 -0.06767119  0.06748961 -0.06728678 -0.06724155
-0.06693875
#...460     ...1330     ...1192     ...1810     ...2146      ...595
...856     ...1837      ...958      ...302     ...1460
#-0.06660861 -0.06629846 -0.06625555 -0.06598256 -0.06588992
-0.06564473 -0.06563471 -0.06553274 -0.06551399 -0.06512148
0.06498773
#...150      ...701     ...2121     ...2074     ...1222      ...779
...1699     ...1772      ...617      ...497      ...133
#-0.06498625 -0.06492982 -0.06492178  0.06491604 -0.06487277
-0.06472775 -0.06465763 -0.06460169  0.06455222 -0.06436520
-0.06435683
#...790     ...2041      ...944      ...596      ...846     ...2127
...1998      ...288     ...1819      ...338     ...1648
#-0.06433532 -0.06421765 -0.06405571 -0.06398910 -0.06398199
-0.06389542 -0.06384724  0.06383403 -0.06382321  0.06373575
0.06372686
#...1114      ...578     ...1962     ...2064      ...350     ...1988
...1488      ...174     ...1204      ...236     ...1277
#0.06369039 -0.06355492  0.06354678 -0.06349043  0.06348340
-0.06344847  0.06341511  0.06337791 -0.06323913 -0.06323738
-0.06285724
#...989     ...1643      ...449     ...1996     ...1566      ...346
...985      ...413     ...1295     ...1228      ...324
#0.06267055 -0.06266111 -0.06264104 -0.06261954 -0.06259102
0.06258168  0.06257610  0.06255301 -0.06221848 -0.06212551
-0.06211489
#...1934      ...213     ...1730      ...294     ...1610     ...1235
...920      ...999      ...122      ...429      ...657
#-0.06211476  0.06210095  0.06206596 -0.06199342 -0.06194333
-0.06194159  0.06191288 -0.06181531 -0.06181490  0.06180706
0.06176491
#...1989      ...481     ...1940     ...2133      ...328     ...1206
...1854       ...32      ...412     ...1265     ...1784
#-0.06169716 -0.06164999 -0.06155948  0.06148717 -0.06147129
0.06143349 -0.06142154 -0.06137188 -0.06136887 -0.06127208
-0.06116913
#...1509
#-0.06108032



#---------------------------------


#with only data of tap 8DAP (root, shoot, light, dark)


library(readxl)
pca_log_woprot_onlytap8DAI <-
read_excel("pca_r_try_29.12.2022.xlsx",
                                         sheet =
```

```
"pca_log_woprot_onlytap8DAI", col_names = FALSE,
                                      col_types = c("text",
                                                   "numeric",
"numeric", "numeric",
                                                   "numeric",
"numeric", "numeric",
                                                   "numeric",
"numeric", "numeric",
                                                   "numeric",
"numeric", "numeric",
                                                   "numeric",
"numeric", "numeric",
                                                   "numeric",
"numeric", "numeric",
                                                   "numeric",
"numeric", "numeric",
                                                   "numeric",
"numeric", "numeric",
                                                   "numeric",
"numeric", "numeric",
                                                   "numeric",
"numeric", "numeric",
                                                   "numeric",
"numeric", "numeric",
                                                   "numeric",
"numeric", "numeric",
                                                   "numeric",
"numeric", "numeric",
                                                   "numeric",
"numeric", "numeric",
                                                   "numeric",
"numeric", "numeric",
                                                   "numeric",
"numeric", "numeric",
                                                   "numeric",
"numeric", "numeric",
                                                   "numeric",
"numeric", "numeric",
                                                   "numeric",
"numeric", "numeric",
                                                   "numeric",
"numeric", "numeric",
                                                   "numeric",
"numeric", "numeric",
                                                   "numeric",
"numeric", "numeric",
                                                   "numeric",
"numeric", "numeric",
                                                   "numeric",
"numeric", "numeric",
                                                   "numeric",
"numeric", "numeric",
```

"numeric", "numeric",
"numeric", "numeric",
"numeric", "numeric",
"numeric", "numeric",
"numeric", "numeric",
"numeric", "numeric",
"numeric", "numeric",
"numeric", "numeric",
"numeric", "numeric",
"numeric", "numeric",
"numeric", "numeric",
"numeric", "numeric",
"numeric", "numeric",
"numeric", "numeric",
"numeric", "numeric",
"numeric", "numeric",
"numeric", "numeric",
"numeric", "numeric",
"numeric", "numeric",
"numeric", "numeric",
"numeric", "numeric",
"numeric", "numeric",
"numeric", "numeric",
"numeric", "numeric",
"numeric", "numeric",
"numeric", "numeric",
"numeric", "numeric",
"numeric",
"numeric",
"numeric",
"numeric",
"numeric",
"numeric",
"numeric",
"numeric",
"numeric",
"numeric",
"numeric",
"numeric",
"numeric",
"numeric",
"numeric",
"numeric",
"numeric",
"numeric",
"numeric",
"numeric",
"numeric",
"numeric",
"numeric",
"numeric",
"numeric",
"numeric",
"numeric",

"numeric", "numeric",
"numeric", "numeric",
"numeric", "numeric",
"numeric", "numeric",
"numeric", "numeric",
"numeric", "numeric",
"numeric", "numeric",
"numeric", "numeric",
"numeric", "numeric",
"numeric", "numeric",
"numeric", "numeric",
"numeric", "numeric",
"numeric", "numeric",
"numeric", "numeric",
"numeric", "numeric",
"numeric", "numeric",
"numeric", "numeric",
"numeric", "numeric",
"numeric", "numeric",
"numeric", "numeric",
"numeric", "numeric",
"numeric", "numeric",
"numeric", "numeric",
"numeric", "numeric",
"numeric", "numeric",
"numeric", "numeric",
"numeric", "numeric",
"numeric",
"numeric",
"numeric",
"numeric",
"numeric",
"numeric",
"numeric",
"numeric",
"numeric",
"numeric",
"numeric",
"numeric",
"numeric",
"numeric",
"numeric",
"numeric",
"numeric",
"numeric",
"numeric",
"numeric",
"numeric",
"numeric",
"numeric",
"numeric",
"numeric",
"numeric",
"numeric",

```
"numeric", "numeric",
"numeric", "numeric",
"numeric", "numeric",
"numeric", "numeric",
"numeric", "numeric",
"numeric", "numeric",
"numeric", "numeric",
"numeric", "numeric",
"numeric", "numeric",
"numeric", "numeric",
"numeric", "numeric",
"numeric", "numeric",
"numeric", "numeric",
"numeric", "numeric",
"numeric", "numeric",
"numeric", "numeric",
"numeric", "numeric",
"numeric", "numeric",
"numeric", "numeric",
"numeric", "numeric",
"numeric", "numeric",
"numeric", "numeric",
"numeric", "numeric",
"numeric", "numeric",
"numeric", "numeric",
"numeric", "numeric",
"numeric", "numeric",
```

```
"numeric",
"numeric",
"numeric",
"numeric",
"numeric",
"numeric",
"numeric",
"numeric",
"numeric",
"numeric",
"numeric",
"numeric",
"numeric",
"numeric",
"numeric",
"numeric",
"numeric",
"numeric",
"numeric",
"numeric",
"numeric",
"numeric",
"numeric",
"numeric",
"numeric",
"numeric",
"numeric",
```

"numeric", "numeric",
"numeric", "numeric",
"numeric", "numeric",
"numeric", "numeric",
"numeric", "numeric",
"numeric", "numeric",
"numeric", "numeric",
"numeric", "numeric",
"numeric", "numeric",
"numeric", "numeric",
"numeric", "numeric",
"numeric", "numeric",
"numeric", "numeric",
"numeric", "numeric",
"numeric", "numeric",
"numeric", "numeric",
"numeric", "numeric",
"numeric", "numeric",
"numeric", "numeric",
"numeric", "numeric",
"numeric", "numeric",
"numeric", "numeric",
"numeric", "numeric",
"numeric", "numeric",
"numeric", "numeric",
"numeric", "numeric",
"numeric", "numeric",

"numeric",
"numeric",
"numeric",
"numeric",
"numeric",
"numeric",
"numeric",
"numeric",
"numeric",
"numeric",
"numeric",
"numeric",
"numeric",
"numeric",
"numeric",
"numeric",
"numeric",
"numeric",
"numeric",
"numeric",
"numeric",
"numeric",
"numeric",
"numeric",
"numeric",
"numeric",
"numeric",

"numeric", "numeric",
"numeric", "numeric",
"numeric", "numeric",
"numeric", "numeric",
"numeric", "numeric",
"numeric", "numeric",
"numeric", "numeric",
"numeric", "numeric",
"numeric", "numeric",
"numeric", "numeric",
"numeric", "numeric",
"numeric", "numeric",
"numeric", "numeric",
"numeric", "numeric",
"numeric", "numeric",
"numeric", "numeric",
"numeric", "numeric",
"numeric", "numeric",
"numeric", "numeric",
"numeric", "numeric",
"numeric", "numeric",
"numeric", "numeric",
"numeric", "numeric",
"numeric", "numeric",
"numeric", "numeric",
"numeric", "numeric",
"numeric", "numeric",
"numeric",
"numeric",
"numeric",
"numeric",
"numeric",
"numeric",
"numeric",
"numeric",
"numeric",
"numeric",
"numeric",
"numeric",
"numeric",
"numeric",
"numeric",
"numeric",
"numeric",
"numeric",
"numeric",
"numeric",
"numeric",
"numeric",
"numeric",
"numeric",
"numeric",
"numeric",
"numeric",

"numeric", "numeric",
"numeric", "numeric",
"numeric", "numeric",
"numeric", "numeric",
"numeric", "numeric",
"numeric", "numeric",
"numeric", "numeric",
"numeric", "numeric",
"numeric", "numeric",
"numeric", "numeric",
"numeric", "numeric",
"numeric", "numeric",
"numeric", "numeric",
"numeric", "numeric",
"numeric", "numeric",
"numeric", "numeric",
"numeric", "numeric",
"numeric", "numeric",
"numeric", "numeric",
"numeric", "numeric",
"numeric", "numeric",
"numeric", "numeric",
"numeric", "numeric",
"numeric", "numeric",
"numeric", "numeric",
"numeric", "numeric",
"numeric", "numeric",
"numeric",
"numeric",
"numeric",
"numeric",
"numeric",
"numeric",
"numeric",
"numeric",
"numeric",
"numeric",
"numeric",
"numeric",
"numeric",
"numeric",
"numeric",
"numeric",
"numeric",
"numeric",
"numeric",
"numeric",
"numeric",
"numeric",
"numeric",
"numeric",
"numeric",
"numeric",
"numeric",

"numeric", "numeric",
"numeric", "numeric",
"numeric", "numeric",
"numeric", "numeric",
"numeric", "numeric",
"numeric", "numeric",
"numeric", "numeric",
"numeric", "numeric",
"numeric", "numeric",
"numeric", "numeric",
"numeric", "numeric",
"numeric", "numeric",
"numeric", "numeric",
"numeric", "numeric",
"numeric", "numeric",
"numeric", "numeric",
"numeric", "numeric",
"numeric", "numeric",
"numeric", "numeric",
"numeric", "numeric",
"numeric", "numeric",
"numeric", "numeric",
"numeric", "numeric",
"numeric", "numeric",
"numeric", "numeric",
"numeric", "numeric",
"numeric", "numeric",
"numeric",
"numeric",
"numeric",
"numeric",
"numeric",
"numeric",
"numeric",
"numeric",
"numeric",
"numeric",
"numeric",
"numeric",
"numeric",
"numeric",
"numeric",
"numeric",
"numeric",
"numeric",
"numeric",
"numeric",
"numeric",
"numeric",
"numeric",
"numeric",
"numeric",
"numeric",
"numeric",

"numeric", "numeric",
"numeric", "numeric",
"numeric", "numeric",
"numeric", "numeric",
"numeric", "numeric",
"numeric", "numeric",
"numeric", "numeric",
"numeric", "numeric",
"numeric", "numeric",
"numeric", "numeric",
"numeric", "numeric",
"numeric", "numeric",
"numeric", "numeric",
"numeric", "numeric",
"numeric", "numeric",
"numeric", "numeric",
"numeric", "numeric",
"numeric", "numeric",
"numeric", "numeric",
"numeric", "numeric",
"numeric", "numeric",
"numeric", "numeric",
"numeric", "numeric",
"numeric", "numeric",
"numeric", "numeric",
"numeric", "numeric",
"numeric", "numeric",
"numeric",
"numeric",
"numeric",
"numeric",
"numeric",
"numeric",
"numeric",
"numeric",
"numeric",
"numeric",
"numeric",
"numeric",
"numeric",
"numeric",
"numeric",
"numeric",
"numeric",
"numeric",
"numeric",
"numeric",
"numeric",
"numeric",
"numeric",
"numeric",
"numeric",
"numeric",
"numeric",

```
"numeric", "numeric",
"numeric", "numeric",
"numeric", "numeric",
"numeric", "numeric",
"numeric", "numeric",
"numeric", "numeric",
"numeric", "numeric",
"numeric", "numeric",
"numeric", "numeric",
"numeric", "numeric",
"numeric", "numeric",
"numeric", "numeric",
"numeric", "numeric",
"numeric", "numeric",
"numeric", "numeric",
"numeric", "numeric",
"numeric", "numeric",
"numeric", "numeric",
"numeric", "numeric",
"numeric", "numeric",
"numeric", "numeric",
"numeric", "numeric",
"numeric", "numeric",
"numeric", "numeric",
"numeric", "numeric",
"numeric", "numeric",
"numeric", "numeric",
"numeric",
"numeric",
"numeric",
"numeric",
"numeric",
"numeric",
"numeric",
"numeric",
"numeric",
"numeric",
"numeric",
"numeric",
"numeric",
"numeric",
"numeric",
"numeric",
"numeric",
"numeric",
"numeric",
"numeric",
"numeric",
"numeric",
"numeric",
"numeric",
"numeric",
"numeric",
"numeric",
```

```
"numeric", "numeric",
"numeric", "numeric",
"numeric", "numeric",
"numeric", "numeric",
"numeric", "numeric",
"numeric", "numeric",
"numeric", "numeric",
"numeric", "numeric",
"numeric", "numeric",
"numeric", "numeric",
"numeric", "numeric",
"numeric", "numeric",
"numeric", "numeric",
"numeric", "numeric",
"numeric", "numeric",
"numeric", "numeric",
"numeric", "numeric",
"numeric", "numeric",
"numeric", "numeric",
"numeric", "numeric",
"numeric", "numeric",
"numeric", "numeric",
"numeric", "numeric",
"numeric", "numeric",
"numeric", "numeric",
"numeric", "numeric",
"numeric", "numeric",
"numeric",
"numeric",
"numeric",
"numeric",
"numeric",
"numeric",
"numeric",
"numeric",
"numeric",
"numeric",
"numeric",
"numeric",
"numeric",
"numeric",
"numeric",
"numeric",
"numeric",
"numeric",
"numeric",
"numeric",
"numeric",
"numeric",
"numeric",
"numeric",
"numeric",
"numeric",
"numeric",
```

"numeric", "numeric",
"numeric", "numeric",
"numeric", "numeric",
"numeric", "numeric",
"numeric", "numeric",
"numeric", "numeric",
"numeric", "numeric",
"numeric", "numeric",
"numeric", "numeric",
"numeric", "numeric",
"numeric", "numeric",
"numeric", "numeric",
"numeric", "numeric",
"numeric", "numeric",
"numeric", "numeric",
"numeric", "numeric",
"numeric", "numeric",
"numeric", "numeric",
"numeric", "numeric",
"numeric", "numeric",
"numeric", "numeric",
"numeric", "numeric",
"numeric", "numeric",
"numeric", "numeric",
"numeric", "numeric",
"numeric", "numeric",
"numeric", "numeric",

"numeric",
"numeric",
"numeric",
"numeric",
"numeric",
"numeric",
"numeric",
"numeric",
"numeric",
"numeric",
"numeric",
"numeric",
"numeric",
"numeric",
"numeric",
"numeric",
"numeric",
"numeric",
"numeric",
"numeric",
"numeric",
"numeric",
"numeric",
"numeric",
"numeric",
"numeric",
"numeric",

"numeric", "numeric",
"numeric", "numeric",
"numeric", "numeric",
"numeric", "numeric",
"numeric", "numeric",
"numeric", "numeric",
"numeric", "numeric",
"numeric", "numeric",
"numeric", "numeric",
"numeric", "numeric",
"numeric", "numeric",
"numeric", "numeric",
"numeric", "numeric",
"numeric", "numeric",
"numeric", "numeric",
"numeric", "numeric",
"numeric", "numeric",
"numeric", "numeric",
"numeric", "numeric",
"numeric", "numeric",
"numeric", "numeric",
"numeric", "numeric",
"numeric", "numeric",
"numeric", "numeric",
"numeric", "numeric",
"numeric", "numeric",
"numeric", "numeric",
"numeric",
"numeric",
"numeric",
"numeric",
"numeric",
"numeric",
"numeric",
"numeric",
"numeric",
"numeric",
"numeric",
"numeric",
"numeric",
"numeric",
"numeric",
"numeric",
"numeric",
"numeric",
"numeric",
"numeric",
"numeric",
"numeric",
"numeric",
"numeric",
"numeric",
"numeric",
"numeric",

"numeric", "numeric",
"numeric", "numeric",
"numeric", "numeric",
"numeric", "numeric",
"numeric", "numeric",
"numeric", "numeric",
"numeric", "numeric",
"numeric", "numeric",
"numeric", "numeric",
"numeric", "numeric",
"numeric", "numeric",
"numeric", "numeric",
"numeric", "numeric",
"numeric", "numeric",
"numeric", "numeric",
"numeric", "numeric",
"numeric", "numeric",
"numeric", "numeric",
"numeric", "numeric",
"numeric", "numeric",
"numeric", "numeric",
"numeric", "numeric",
"numeric", "numeric",
"numeric", "numeric",
"numeric", "numeric",
"numeric", "numeric",
"numeric", "numeric",
"numeric",
"numeric",
"numeric",
"numeric",
"numeric",
"numeric",
"numeric",
"numeric",
"numeric",
"numeric",
"numeric",
"numeric",
"numeric",
"numeric",
"numeric",
"numeric",
"numeric",
"numeric",
"numeric",
"numeric",
"numeric",
"numeric",
"numeric",
"numeric",
"numeric",
"numeric",
"numeric",

"numeric",
"numeric", "numeric",
"numeric",
"numeric", "numeric",
"numeric",
"numeric", "numeric",
"numeric",
"numeric", "numeric",
"numeric",
"numeric", "numeric",
"numeric",
"numeric", "numeric",
"numeric",
"numeric", "numeric",
"numeric",
"numeric", "numeric",
"numeric",
"numeric", "numeric",
"numeric",
"numeric", "numeric",
"numeric",
"numeric", "numeric",
"numeric",
"numeric", "numeric",
"numeric",
"numeric", "numeric",
"numeric",
"numeric", "numeric",
"numeric",
"numeric", "numeric",
"numeric",
"numeric", "numeric",
"numeric",
"numeric", "numeric",
"numeric",
"numeric", "numeric",
"numeric",
"numeric", "numeric",
"numeric",
"numeric", "numeric",
"numeric",
"numeric", "numeric",
"numeric",
"numeric", "numeric",
"numeric",
"numeric", "numeric",
"numeric",
"numeric", "numeric",
"numeric",
"numeric", "numeric",
"numeric",
"numeric", "numeric",
"numeric",
"numeric", "numeric",

```
"numeric", "numeric",
"numeric", "numeric",
"numeric", "numeric",
"numeric", "numeric",
"numeric", "numeric",
"numeric", "numeric",
"numeric", "numeric",
"numeric", "numeric",
"numeric", "numeric",
"numeric", "numeric",
"numeric", "numeric",
"numeric", "numeric",
"numeric", "numeric",
"numeric", "numeric",
"numeric", "numeric",
"numeric", "numeric",
"numeric", "numeric",
"numeric", "numeric",
"numeric", "numeric",
"numeric", "numeric",
"numeric", "numeric",
"numeric", "numeric",
"numeric", "numeric",
"numeric", "numeric",
"numeric", "numeric",
"numeric", "numeric",
"numeric", "numeric",
```

```
"numeric",
"numeric",
"numeric",
"numeric",
"numeric",
"numeric",
"numeric",
"numeric",
"numeric",
"numeric",
"numeric",
"numeric",
"numeric",
"numeric",
"numeric",
"numeric",
"numeric",
"numeric",
"numeric",
"numeric",
"numeric",
"numeric",
"numeric",
"numeric",
"numeric",
"numeric",
"numeric",
```

"numeric",
"numeric", "numeric",
"numeric",
"numeric", "numeric",
"numeric",
"numeric", "numeric",
"numeric",
"numeric", "numeric",
"numeric",
"numeric", "numeric",
"numeric",
"numeric", "numeric",
"numeric",
"numeric", "numeric",
"numeric",
"numeric", "numeric",
"numeric",
"numeric", "numeric",
"numeric",
"numeric", "numeric",
"numeric",
"numeric", "numeric",
"numeric",
"numeric", "numeric",
"numeric",
"numeric", "numeric",
"numeric",
"numeric", "numeric",
"numeric",
"numeric", "numeric",
"numeric",
"numeric", "numeric",
"numeric",
"numeric", "numeric",
"numeric",
"numeric", "numeric",
"numeric",
"numeric", "numeric",
"numeric",
"numeric", "numeric",
"numeric",
"numeric", "numeric",
"numeric",
"numeric", "numeric",
"numeric",
"numeric", "numeric",
"numeric",
"numeric", "numeric",
"numeric",
"numeric", "numeric",
"numeric",
"numeric", "numeric",
"numeric",
"numeric", "numeric",

"numeric",
"numeric", "numeric",
"numeric",
"numeric", "numeric",
"numeric",
"numeric", "numeric",
"numeric",
"numeric", "numeric",
"numeric",
"numeric", "numeric",
"numeric",
"numeric", "numeric",
"numeric",
"numeric", "numeric",
"numeric",
"numeric", "numeric",
"numeric",
"numeric", "numeric",
"numeric",
"numeric", "numeric",
"numeric",
"numeric", "numeric",
"numeric",
"numeric", "numeric",
"numeric",
"numeric", "numeric",
"numeric",
"numeric", "numeric",
"numeric",
"numeric", "numeric",
"numeric",
"numeric", "numeric",
"numeric",
"numeric", "numeric",
"numeric",
"numeric", "numeric",
"numeric",
"numeric", "numeric",
"numeric",
"numeric", "numeric",
"numeric",
"numeric", "numeric",
"numeric",
"numeric", "numeric",
"numeric",
"numeric", "numeric",
"numeric",
"numeric", "numeric",
"numeric",
"numeric", "numeric",
"numeric",
"numeric", "numeric",
"numeric",
"numeric", "numeric",

"numeric", "numeric",
"numeric", "numeric",
"numeric", "numeric",
"numeric", "numeric",
"numeric", "numeric",
"numeric", "numeric",
"numeric", "numeric",
"numeric", "numeric",
"numeric", "numeric",
"numeric", "numeric",
"numeric", "numeric",
"numeric", "numeric",
"numeric", "numeric",
"numeric", "numeric",
"numeric", "numeric",
"numeric", "numeric",
"numeric", "numeric",
"numeric", "numeric",
"numeric", "numeric",
"numeric", "numeric",
"numeric", "numeric",
"numeric", "numeric",
"numeric", "numeric",
"numeric", "numeric",
"numeric", "numeric",
"numeric", "numeric",
"numeric", "numeric",
"numeric",
"numeric",
"numeric",
"numeric",
"numeric",
"numeric",
"numeric",
"numeric",
"numeric",
"numeric",
"numeric",
"numeric",
"numeric",
"numeric",
"numeric",
"numeric",
"numeric",
"numeric",
"numeric",
"numeric",
"numeric",
"numeric",
"numeric",
"numeric",
"numeric",
"numeric",
"numeric",

"numeric", "numeric",
"numeric", "numeric",
"numeric", "numeric",
"numeric", "numeric",
"numeric", "numeric",
"numeric", "numeric",
"numeric", "numeric",
"numeric", "numeric",
"numeric", "numeric",
"numeric", "numeric",
"numeric", "numeric",
"numeric", "numeric",
"numeric", "numeric",
"numeric", "numeric",
"numeric", "numeric",
"numeric", "numeric",
"numeric", "numeric",
"numeric", "numeric",
"numeric", "numeric",
"numeric", "numeric",
"numeric", "numeric",
"numeric", "numeric",
"numeric", "numeric",
"numeric", "numeric",
"numeric", "numeric",
"numeric", "numeric",
"numeric", "numeric",
"numeric",
"numeric",
"numeric",
"numeric",
"numeric",
"numeric",
"numeric",
"numeric",
"numeric",
"numeric",
"numeric",
"numeric",
"numeric",
"numeric",
"numeric",
"numeric",
"numeric",
"numeric",
"numeric",
"numeric",
"numeric",
"numeric",
"numeric",
"numeric",
"numeric",
"numeric",
"numeric",

"numeric", "numeric",
"numeric", "numeric",
"numeric", "numeric",
"numeric", "numeric",
"numeric", "numeric",
"numeric", "numeric",
"numeric", "numeric",
"numeric", "numeric",
"numeric", "numeric",
"numeric", "numeric",
"numeric", "numeric",
"numeric", "numeric",
"numeric", "numeric",
"numeric", "numeric",
"numeric", "numeric",
"numeric", "numeric",
"numeric", "numeric",
"numeric", "numeric",
"numeric", "numeric",
"numeric", "numeric",
"numeric", "numeric",
"numeric", "numeric",
"numeric", "numeric",
"numeric", "numeric",
"numeric", "numeric",
"numeric", "numeric",
"numeric", "numeric",
"numeric",
"numeric",
"numeric",
"numeric",
"numeric",
"numeric",
"numeric",
"numeric",
"numeric",
"numeric",
"numeric",
"numeric",
"numeric",
"numeric",
"numeric",
"numeric",
"numeric",
"numeric",
"numeric",
"numeric",
"numeric",
"numeric",
"numeric",
"numeric",
"numeric",
"numeric",
"numeric",

"numeric", "numeric",
"numeric", "numeric",
"numeric", "numeric",
"numeric", "numeric",
"numeric", "numeric",
"numeric", "numeric",
"numeric", "numeric",
"numeric", "numeric",
"numeric", "numeric",
"numeric", "numeric",
"numeric", "numeric",
"numeric", "numeric",
"numeric", "numeric",
"numeric", "numeric",
"numeric", "numeric",
"numeric", "numeric",
"numeric", "numeric",
"numeric", "numeric",
"numeric", "numeric",
"numeric", "numeric",
"numeric", "numeric",
"numeric", "numeric",
"numeric", "numeric",
"numeric", "numeric",
"numeric", "numeric",
"numeric", "numeric",
"numeric", "numeric",
"numeric",
"numeric",
"numeric",
"numeric",
"numeric",
"numeric",
"numeric",
"numeric",
"numeric",
"numeric",
"numeric",
"numeric",
"numeric",
"numeric",
"numeric",
"numeric",
"numeric",
"numeric",
"numeric",
"numeric",
"numeric",
"numeric",
"numeric",
"numeric",
"numeric",
"numeric",
"numeric",

```
"numeric", "numeric",
"numeric", "numeric",
"numeric", "numeric",
"numeric", "numeric",
"numeric", "numeric",
"numeric", "numeric",
"numeric", "numeric",
"numeric", "numeric",
"numeric", "numeric",
"numeric", "numeric",
"numeric", "numeric",
"numeric", "numeric",
"numeric", "numeric",
"numeric", "numeric",
"numeric", "numeric",
"numeric", "numeric",
"numeric", "numeric",
"numeric", "numeric",
"numeric", "numeric",
"numeric", "numeric",
"numeric", "numeric",
"numeric", "numeric",
"numeric", "numeric",
"numeric", "numeric",
"numeric", "numeric",
"numeric", "numeric",
"numeric", "numeric",
```

```
"numeric",
"numeric",
"numeric",
"numeric",
"numeric",
"numeric",
"numeric",
"numeric",
"numeric",
"numeric",
"numeric",
"numeric",
"numeric",
"numeric",
"numeric",
"numeric",
"numeric",
"numeric",
"numeric",
"numeric",
"numeric",
"numeric",
"numeric",
"numeric",
"numeric",
"numeric",
"numeric",
```

"numeric", "numeric",
"numeric", "numeric",
"numeric", "numeric",
"numeric", "numeric",
"numeric", "numeric",
"numeric", "numeric",
"numeric", "numeric",
"numeric", "numeric",
"numeric", "numeric",
"numeric", "numeric",
"numeric", "numeric",
"numeric", "numeric",
"numeric", "numeric",
"numeric", "numeric",
"numeric", "numeric",
"numeric", "numeric",
"numeric", "numeric",
"numeric", "numeric",
"numeric", "numeric",
"numeric", "numeric",
"numeric", "numeric",
"numeric", "numeric",
"numeric", "numeric",
"numeric", "numeric",
"numeric", "numeric",
"numeric", "numeric",
"numeric", "numeric",
"numeric",
"numeric",
"numeric",
"numeric",
"numeric",
"numeric",
"numeric",
"numeric",
"numeric",
"numeric",
"numeric",
"numeric",
"numeric",
"numeric",
"numeric",
"numeric",
"numeric",
"numeric",
"numeric",
"numeric",
"numeric",
"numeric",
"numeric",
"numeric",
"numeric",
"numeric",
"numeric",

"numeric", "numeric",
"numeric", "numeric",
"numeric", "numeric",
"numeric", "numeric",
"numeric", "numeric",
"numeric", "numeric",
"numeric", "numeric",
"numeric", "numeric",
"numeric", "numeric",
"numeric", "numeric",
"numeric", "numeric",
"numeric", "numeric",
"numeric", "numeric",
"numeric", "numeric",
"numeric", "numeric",
"numeric", "numeric",
"numeric", "numeric",
"numeric", "numeric",
"numeric", "numeric",
"numeric", "numeric",
"numeric", "numeric",
"numeric", "numeric",
"numeric", "numeric",
"numeric", "numeric",
"numeric", "numeric",
"numeric", "numeric",
"numeric", "numeric",
"numeric",
"numeric",
"numeric",
"numeric",
"numeric",
"numeric",
"numeric",
"numeric",
"numeric",
"numeric",
"numeric",
"numeric",
"numeric",
"numeric",
"numeric",
"numeric",
"numeric",
"numeric",
"numeric",
"numeric",
"numeric",
"numeric",
"numeric",
"numeric",
"numeric",
"numeric",
"numeric",

"numeric", "numeric",
"numeric", "numeric",
"numeric", "numeric",
"numeric", "numeric",
"numeric", "numeric",
"numeric", "numeric",
"numeric", "numeric",
"numeric", "numeric",
"numeric", "numeric",
"numeric", "numeric",
"numeric", "numeric",
"numeric", "numeric",
"numeric", "numeric",
"numeric", "numeric",
"numeric", "numeric",
"numeric", "numeric",
"numeric", "numeric",
"numeric", "numeric",
"numeric", "numeric",
"numeric", "numeric",
"numeric", "numeric",
"numeric", "numeric",
"numeric", "numeric",
"numeric", "numeric",
"numeric", "numeric",
"numeric", "numeric",
"numeric", "numeric",
"numeric",
"numeric",
"numeric",
"numeric",
"numeric",
"numeric",
"numeric",
"numeric",
"numeric",
"numeric",
"numeric",
"numeric",
"numeric",
"numeric",
"numeric",
"numeric",
"numeric",
"numeric",
"numeric",
"numeric",
"numeric",
"numeric",
"numeric",
"numeric",
"numeric",
"numeric",
"numeric",

```
                                                                "numeric",
"numeric", "numeric",
                                                                "numeric",
"numeric", "numeric",
                                                                "numeric",
"numeric", "numeric",
                                                                "numeric",
"numeric", "numeric",
                                                                "numeric",
"numeric", "numeric",
                                                                "numeric",
"numeric", "numeric",
                                                                "numeric",
"numeric", "numeric",
                                                                "numeric",
"numeric", "numeric",
                                                                "numeric",
"numeric", "numeric",
                                                                "numeric",
"numeric", "numeric",
                                                                "numeric",
"numeric", "numeric",
                                                                "numeric",
"numeric", "numeric",
                                                                "numeric",
"numeric", "numeric",
                                                                "numeric",
"numeric", "numeric",
                                                                "numeric",
"numeric", "numeric",
                                                                "numeric",
"numeric", "numeric",
                                                                "numeric",
"numeric", "numeric",
                                                                "numeric",
"numeric", "numeric",
                                                                "numeric"))

View(pca_log_woprot_onlytap8DAI)
pca_log_woprot_onlytap8DAI
pca_log_woprot_onlytap8DAI_matrix<-
as.matrix(pca_log_woprot_onlytap8DAI)
#pca <- prcomp((pca_log_woprot), scale=TRUE)
pca_log_woprot_onlytap8DAI_matrix



pca_log_woprot_onlytap8DAI_new <- pca_log_woprot_onlytap8DAI[ ,
unlist(lapply(pca_log_woprot_onlytap8DAI,             # Remove non-
numeric columns

is.numeric))]
pca_log_woprot_onlytap8DAI_new
pca_03012023_onlytap8DAI <- prcomp(pca_log_woprot_onlytap8DAI_new)
```

```
#worked!!!!! (https://statisticsglobe.com/r-error-colmeans-x-must-
be-numeric)
plot(pca_03012023_onlytap8DAI$x[,1], pca_03012023_onlytap8DAI$x[,2])
#worked!!!!   https://www.youtube.com/watch?v=0Jp4gsfOLMs&t=153s
pca_03012023_onlytap8DAI.var <-pca_03012023_onlytap8DAI$sdev^2
pca_03012023_onlytap8DAI.var.per <-
round(pca_03012023_onlytap8DAI.var/
sum(pca_03012023_onlytap8DAI.var)*100,1)
barplot(pca_03012023_onlytap8DAI.var.per, main="Scree Plot",
xlab="Principal Component", ylab="Percent Variation") # i have PCs
for every sample....!

pca_03012023_onlytap8DAI.var.per

#pca plot only tap



group_onlytap8DAI <- c("8DAI_tap_DR" ,
                        "8DAI_tap_DR",   "8DAI_tap_DR",
"8DAI_tap_DR",   "8DAI_tap_DS",
                        "8DAI_tap_DS",   "8DAI_tap_DS" ,
"8DAI_tap_DS",   "8DAI_tap_LR",   "8DAI_tap_LR",   "8DAI_tap_LR",
                        "8DAI_tap_LR",   "8DAI_tap_LS",
"8DAI_tap_LS",   "8DAI_tap_LS",   "8DAI_tap_LS")
group_onlytap8DAI


plot(pca_03012023_onlytap8DAI$x[,1],pca_03012023_onlytap8DAI$x[,2],
     col=factor(group_onlytap8DAI),main="PCA plot 8DAI tap - light -
dark",
     pch=19, xlab="PC1 (68.7%)",
     ylab="PC2 (17.6%)")

library(ggfortify)
autoplot(pca_03012023_onlytap8DAI, data =
pca_log_woprot_onlytap8DAI_new, main="PCA plot 8DAI tap - light -
dark", shape = FALSE, label.size = 3)# hier andere dastellweise mit
Zahlen als beschriftung... 1-53

#die top 10 und top 100 proteine...
# für die x achse... durck nach root oder shoot...

loading_scores_tap8DAI <- pca_03012023_onlytap8DAI$rotation[,1]
protein_scores_tap8DAI <- abs(loading_scores_tap8DAI) #proteins that
push samples to the left side of the graph will have a large
negative values and proteins that push samples to
# the right will have a large positive values.
# since we are interested in both sets, of proteins, we will use the
abs() function to sort based on the
# numbers magnitude rather from high to low
protein_score_ranked_tap8DAI <- sort(protein_scores_tap8DAI,
decreasing=TRUE) #here we sort the magnitudes of the loading scores
from high to low.
```

```
top_10_proteins_tap8DAI <- names(protein_score_ranked_tap8DAI[1:10]) #now we get the names of the top 10 proteins with the largest loading score magnitudes.
top_10_proteins_tap8DAI

pca_03012023_onlytap8DAI$rotation[top_10_proteins_tap8DAI,1] ## show the scores (and +/- sign)

#top_10_proteins_tap8DAI
#[1] "...2021" "...513"  "...1505" "...1714" "...1770" "...1644" "...1929" "...1629" "...1824" "...1550"
#> pca_03012023_onlytap8DAI$rotation[top_10_proteins_tap8DAI,1] ## show the scores (and +/- sign)
#...2021      ...513     ...1505     ...1714     ...1770     ...1644 ...1929     ...1629     ...1824     ...1550
#0.05740382  0.05549062  0.05512716  0.05460796 -0.05445144 0.05346398  0.05332088  0.05328472  0.05306358  0.05297635



#---

top_100_proteins_tap8DAI <- names(protein_score_ranked_tap8DAI[1:100]) #now we get the names of the top 10 proteins with the largest loading score magnitudes.
top_100_proteins_tap8DAI

pca_03012023_onlytap8DAI$rotation[top_100_proteins_tap8DAI,1] ## show the scores (and +/- sign)



#top_100_proteins_tap8DAI
#[1] "...2021" "...513"  "...1505" "...1714" "...1770" "...1644" "...1929" "...1629" "...1824" "...1550" "...1233" "...1611" "...1463"
#[14] "...1763" "...1587" "...829"  "...514"  "...506"  "...919" "...82"   "...876"  "...295"  "...1100" "...1341" "...319"  "...238"
#[27] "...923"  "...1621" "...718"  "...805"  "...1289" "...236" "...1938" "...801"  "...277"  "...661"  "...1835" "...316"  "...840"
#[40] "...60"   "...1122" "...286"  "...62"   "...2158" "...1859" "...1489" "...61"   "...366"  "...1827" "...1567" "...2141" "...2140"
#[53] "...784"  "...374"  "...47"   "...969"  "...2000" "...1969" "...913"  "...697"  "...2153" "...1133" "...200"  "...2027" "...434"
#[66] "...31"   "...502"  "...5"    "...1183" "...105"  "...135" "...853"  "...57"   "...1447" "...1172" "...1003" "...1563" "...630"
#[79] "...487"  "...1823" "...1320" "...208"  "...1574" "...999" "...553"  "...289"  "...1727" "...1690" "...217"  "...1909" "...1846"
#[92] "...149"  "...1955" "...739"  "...414"  "...642"  "...1803" "...1575" "...1786" "...326"
#> pca_03012023_onlytap8DAI$rotation[top_100_proteins_tap8DAI,1] ## show the scores (and +/- sign)
```

```
#...2021      ...513     ...1505     ...1714     ...1770     ...1644
...1929     ...1629     ...1824     ...1550     ...1233
#0.05740382  0.05549062  0.05512716  0.05460796 -0.05445144
0.05346398  0.05332088  0.05328472  0.05306358  0.05297635
0.05273295
#...1611     ...1463     ...1763     ...1587      ...829      ...514
...506      ...919       ...82      ...876      ...295
#0.05272251  0.05264980  0.05249269  0.05201395  0.05196803
0.05183077  0.05170252  0.05159657  0.05146126  0.05141456
0.05124710
#...1100     ...1341      ...319      ...238      ...923     ...1621
...718      ...805     ...1289      ...236     ...1938
#0.05116487  0.05112944  0.05109164  0.05104413  0.05095518
0.05093180 -0.05070970  0.05067074 -0.05045281 -0.05031228
-0.05029921
#...801      ...277      ...661     ...1835      ...316      ...840
...60     ...1122      ...286       ...62     ...2158
#0.05027059  0.05024243  0.05024123  0.05014251 -0.05014067
0.05007813 -0.04993609  0.04989180  0.04985187  0.04977995
0.04977732
#...1859     ...1489       ...61      ...366     ...1827     ...1567
...2141     ...2140      ...784      ...374       ...47
#0.04977059  0.04969830  0.04965274  0.04960880  0.04958311
0.04954813 -0.04945700  0.04928549  0.04924733  0.04922396
0.04919448
#...969     ...2000     ...1969      ...913      ...697     ...2153
...1133      ...200     ...2027      ...434       ...31
#0.04918103  0.04917146  0.04909808  0.04909015  0.04898713
0.04889140  0.04883946 -0.04882690 -0.04876626  0.04864258
0.04858612
#...502        ...5     ...1183      ...105      ...135      ...853
...57     ...1447     ...1172     ...1003     ...1563
#-0.04853084  0.04851549  0.04848372  0.04847461  0.04840045
0.04838237 -0.04828591  0.04824848  0.04817237  0.04816256
0.04805171
#...630      ...487     ...1823     ...1320      ...208     ...1574
...999      ...553      ...289     ...1727     ...1690
#0.04801013  0.04799238  0.04793838  0.04785041  0.04773291
0.04771680 -0.04769876  0.04768054  0.04766868  0.04764168
0.04759434
#...217     ...1909     ...1846      ...149     ...1955      ...739
...414      ...642     ...1803     ...1575     ...1786
#0.04757967 -0.04749470  0.04742629  0.04739418  0.04738262
0.04737696  0.04736829  0.04735970  0.04735433  0.04734358
0.04732578
#...326
#0.04730142


#---



#die top 10 und top 100 proteine...
```

```
# für die y achse... durck nach light  oder dark...


loading_scores_tap8DAI_y <- pca_03012023_onlytap8DAI$rotation[,2]
protein_scores_tap8DAI_y <- abs(loading_scores_tap8DAI_y) #proteins
that push samples to the left side of the graph will have a large
negative values and proteins that push samples to
# the right will have a large positive values.
# since we are interested in both sets, of proteins, we will use the
abs() function to sort based on the
# numbers magnitude rather from high to low
protein_score_ranked_tap8DAI_y <- sort(protein_scores_tap8DAI_y,
decreasing=TRUE) #here we sort the magnitudes of the loading scores
from high to low.
top_10_proteins_tap8DAI_y <-
names(protein_score_ranked_tap8DAI_y[1:10]) #now we get the names of
the top 10 proteins with the largest loading score magnitudes.
top_10_proteins_tap8DAI_y

pca_03012023_onlytap8DAI$rotation[top_10_proteins_tap8DAI_y,2] ##
show the scores (and +/- sign)

#top_10_proteins_tap8DAI_y
#[1] "...1962" "...1265" "...747"  "...296"  "...905"  "...723"
"...686"  "...1044" "...546"  "...1952"
#> pca_03012023_onlytap8DAI$rotation[top_10_proteins_tap8DAI_y,2] ##
show the scores (and +/- sign)
#...1962     ...1265      ...747      ...296      ...905      ...723
...686     ...1044      ...546     ...1952
#-0.09161793  0.09073965  0.09021984  0.09015242  0.09006822
0.09006420  0.08953397  0.08920216  0.08878779  0.08857695



top_100_proteins_tap8DAI_y <-
names(protein_score_ranked_tap8DAI_y[1:100]) #now we get the names
of the top 10 proteins with the largest loading score magnitudes.
top_100_proteins_tap8DAI_y

pca_03012023_onlytap8DAI$rotation[top_100_proteins_tap8DAI_y,2] ##
show the scores (and +/- sign)


#top_100_proteins_tap8DAI_y
#[1] "...1962" "...1265" "...747"  "...296"  "...905"  "...723"
"...686"  "...1044" "...546"  "...1952" "...1059" "...930"
"...1532"
#[14] "...1203" "...1581" "...483"  "...2100" "...1596" "...1890"
"...275"  "...2127" "...352"  "...1754" "...1224" "...1693"
"...1779"
#[27] "...1758" "...2067" "...653"  "...1713" "...860"  "...680"
"...323"  "...1178" "...842"  "...1506" "...34"   "...2015" "...750"
#[40] "...1881" "...25"   "...738"  "...1145" "...186"  "...824"
"...1388" "...1478" "...1732" "...1837" "...1641" "...1804"
```

```
"...1848"
#[53] "...1066" "...463"  "...1164" "...941"  "...1521" "...712"
"...1071" "...1119" "...401"  "...1321" "...1024" "...1858"
"...1460"
#[66] "...675"  "...1752" "...1025" "...1413" "...1462" "...636"
"...1418" "...2149" "...963"  "...2113" "...1879" "...1085" "...823"
#[79] "...1402" "...799"  "...863"  "...2069" "...411"  "...699"
"...1046" "...157"  "...1313" "...2104" "...1627" "...706"  "...618"
#[92] "...1935" "...1307" "...1314" "...2053" "...559"  "...161"
"...1975" "...752"  "...1102"
#> pca_03012023_onlytap8DAI$rotation[top_100_proteins_tap8DAI_y,2]
## show the scores (and +/- sign)
#...1962     ...1265      ...747      ...296      ...905      ...723
...686     ...1044      ...546     ...1952     ...1059
#-0.09161793  0.09073965  0.09021984  0.09015242  0.09006822
0.09006420  0.08953397  0.08920216  0.08878779  0.08857695
-0.08829680
#...930     ...1532     ...1203     ...1581      ...483     ...2100
...1596     ...1890      ...275     ...2127      ...352
#0.08679327 -0.08668126 -0.08660276  0.08647333 -0.08605620
0.08603516  0.08583577  0.08558922  0.08550318  0.08541406
0.08531038
#...1754     ...1224     ...1693     ...1779     ...1758     ...2067
...653     ...1713     ...860      ...680      ...323
#0.08494560  0.08435024 -0.08407164 -0.08404719 -0.08374122
-0.08363361  0.08362175  0.08315919 -0.08225822 -0.08209071
-0.08013663
#...1178      ...842     ...1506       ...34     ...2015      ...750
...1881       ...25      ...738     ...1145      ...186
#-0.07965774  0.07923495 -0.07882837 -0.07845208  0.07641887
-0.06419439 -0.06380849  0.06084324  0.05979855 -0.05971185
-0.05878776
#...824     ...1388     ...1478     ...1732     ...1837     ...1641
...1804     ...1848     ...1066      ...463     ...1164
#0.05840019  0.05824051 -0.05814090 -0.05780082  0.05735974
-0.05715122  0.05699927  0.05691188  0.05679282  0.05652887
0.05642007
#...941     ...1521      ...712     ...1071     ...1119      ...401
...1321     ...1024     ...1858     ...1460      ...675
#-0.05620413  0.05603051  0.05592962 -0.05591948 -0.05591636
0.05577489 -0.05561888  0.05560624 -0.05541919  0.05539565
0.05538074
#...1752     ...1025     ...1413     ...1462      ...636     ...1418
...2149      ...963     ...2113     ...1879     ...1085
#0.05535481  0.05527011  0.05526383 -0.05519335  0.05512009
0.05480681  0.05478102  0.05475300  0.05464457 -0.05440009
0.05439384
#...823     ...1402      ...799      ...863     ...2069      ...411
...699    ...1046      ...157     ...1313     ...2104
#-0.05421908  0.05416847 -0.05408502 -0.05390399 -0.05383717
0.05374097  0.05373247  0.05371360 -0.05370592  0.05367055
0.05360285
#...1627      ...706      ...618     ...1935     ...1307     ...1314
...2053      ...559      ...161     ...1975      ...752
```

```
#0.05358741 -0.05352899 -0.05345137  0.05344175  0.05341420
-0.05330230  0.05326695 -0.05322482  0.05317446 -0.05315029
-0.05307914
#...1102
#-0.05307517

#-----------
#---------------------------------------
#--------------------------------------

#04.01.2023 + 12.01.2023
#Try to make loadings plots... with the arrows

install.packages("RColorBrewer")
library(RColorBrewer)
install.packages("autoplot")
library(autoplot)

#only tap
library(ggfortify)
autoplot(pca02012023_onlytap, data = pca_log_woprot_onlytap_new,
main="PCA plot tap - light - dark", loadings = TRUE,
loadings.colour='magenta')
autoplot(pca02012023_onlytap, data = pca_log_woprot_onlytap_new,
main="PCA plot tap - light - dark", loadings = TRUE,
loadings.colour='darkorchid4',loadings.label=TRUE,
loadings.label.colour='blue',loadings.label.size=3)#with numbers at
arrows


#only tap 16DAI-...


library(ggfortify)
autoplot(pca03.01.2023_extract, data = pca_log_woprot_new[16:29,],
main="PCA plot 16DAI tap - light - dark", loadings = TRUE,
loadings.colour='magenta')

#for 8DAI

autoplot(pca_03012023_onlytap8DAI, data =
pca_log_woprot_onlytap8DAI_new, main="PCA plot 8DAI tap - light -
dark", loadings = TRUE, loadings.colour='magenta')

#---------------------------
#----------------------------




#-------------------------------
#13.01.2023
#Contributions blots...
```

```
#http://www.sthda.com/english/wiki/fviz-contrib-quick-visualization-of-row-column-contributions-r-software-and-data-mining

install.packages("ggplot2")
library(ggplot2)

install.packages("devtools")
library(devtools)
devtools::install_github("kassambara/factoextra")
 install.packages("factoextra")
library(factoextra)



 #only tap
 library(ggfortify)

 fviz_contrib(pca02012023_onlytap, choice="var", axes = 1, color = "steelblue") #all
 fviz_contrib(pca02012023_onlytap, choice="var", axes = 1, color = "steelblue", top=30) #top30

 fviz_contrib(pca02012023_onlytap, choice="var", axes = 2, color = "steelblue") #all
 fviz_contrib(pca02012023_onlytap, choice="var", axes = 2, color = "steelblue", top=30)  #top30
```
